# Plant peptides – redefining an area of ribosomally synthesized and post-translationally modified peptides

**DOI:** 10.1039/d3np00042g

**Published:** 2024-07-17

**Authors:** Jonathan R. Chekan, Lisa S. Mydy, Michael A. Pasquale, Roland D. Kersten

**Affiliations:** a Department of Chemistry and Biochemistry, University of North Carolina at Greensboro, Greensboro, NC, USA; b Department of Medicinal Chemistry, University of Michigan, Ann Arbor, MI, USA

## Abstract

Covering 1965 to February 2024

Plants are prolific peptide chemists and are known to make thousands of different peptidic molecules. These peptides vary dramatically in their size, chemistry, and bioactivity. Despite their differences, all plant peptides to date are biosynthesized as ribosomally synthesized and post-translationally modified peptides (RiPPs). Decades of research in plant RiPP biosynthesis have extended the definition and scope of RiPPs from microbial sources, establishing paradigms and discovering new families of biosynthetic enzymes. The discovery and elucidation of plant peptide pathways is challenging due to repurposing and evolution of housekeeping genes as both precursor peptides and biosynthetic enzymes and due to the low rates of gene clustering in plants. In this review, we highlight the chemistry, biosynthesis, and function of the known RiPP classes from plants and recommend a nomenclature for the recent addition of BURP-domain-derived RiPPs termed burpitides. Burpitides are an emerging family of cyclic plant RiPPs characterized by macrocyclic crosslinks between tyrosine or tryptophan side chains and other amino acid side chains or their peptide backbone that are formed by copper-dependent BURP-domain-containing proteins termed burpitide cyclases. Finally, we review the discovery of plant RiPPs through bioactivity-guided, structure-guided, and gene-guided approaches.

## 1. Introduction

**Fig. 1:**
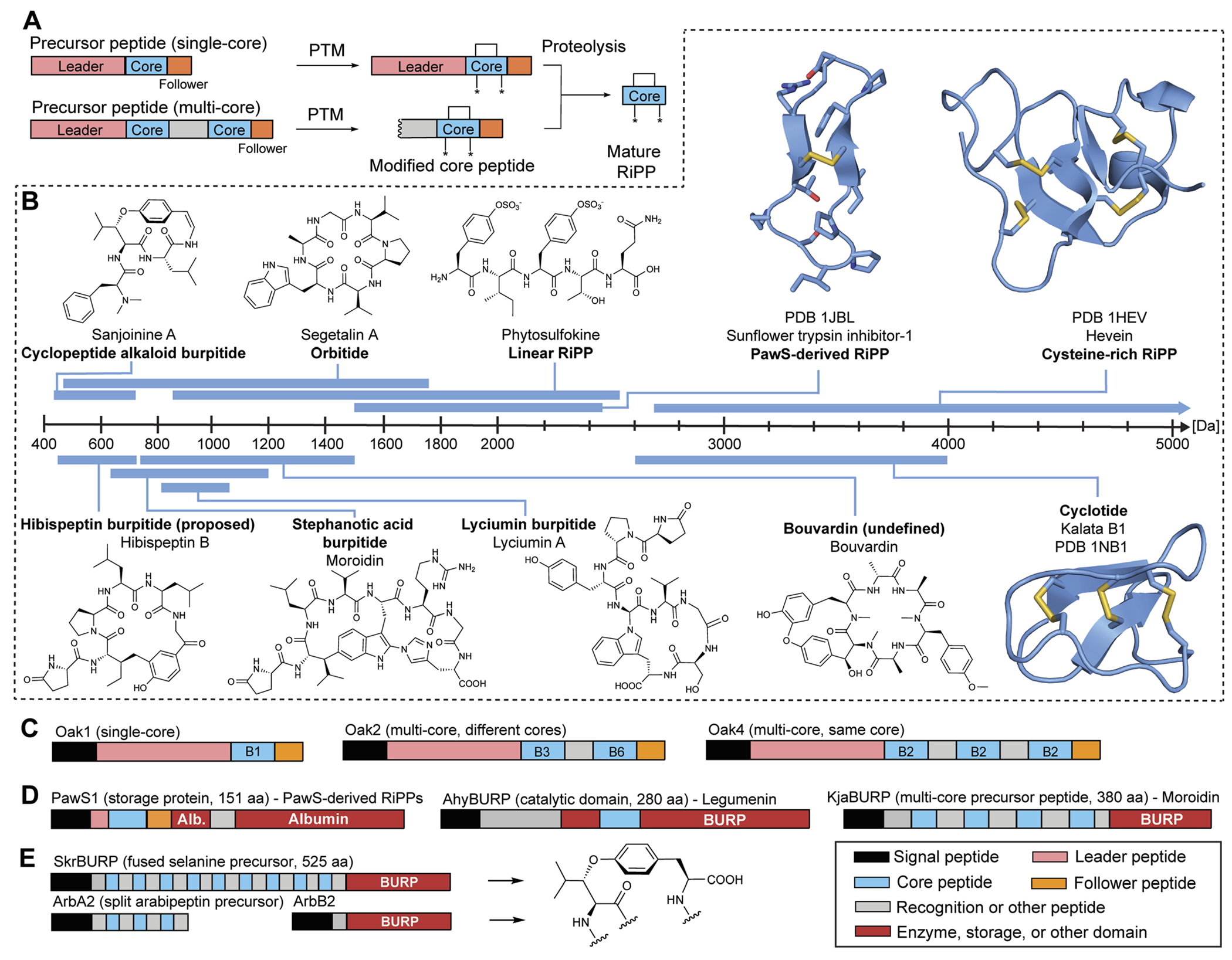
Plant RiPP chemistry and biosynthesis. (A) RiPP biosynthetic dogma.^1,2^ (B) Representative structures of plant RiPPs and their corresponding size ranges. Peptide mass ranges were determined based on plant peptide databases and studies.^18,26,27^ (C) Single-core and multi-core precursor peptides of kalata cyclotides. B1 represents kalata B1 core peptide. (D) Precursor proteins with storage, catalytic, and multi-core domains. (E) Fused and split precursor peptides in cyclopeptide alkaloid RiPP biosynthesis. Disulfide bond formation and pyroglutamate formation can occur enzymatically or spontaneously. Abbreviations: PTM – post-translational modification, alb. – albumin, core – core peptide, Leader – leader peptide, Follower – follower peptide.

RiPPs are a large, diverse group of natural products which are produced by bacteria, archaea, fungi, animals, and plants. The defining RiPP biosynthetic feature is the ribosomal generation of a precursor peptide which is post-translationally modified (PTM) and proteolytically cleaved to yield a mature RiPP natural product^1,2^ (Fig. 1A). Plants are a prolific source of peptide chemistry^3^ and the investigation of underlying peptide biosynthetic genes has revealed that plants produce peptide natural products exclusively *via* the ribosomal pathway based on current knowledge.^4–21^ Plant RiPPs have yielded sustainable pest control agents in agriculture such as Sero-X,^22,23^ and they have emerged as promising lead compounds for medicinal applications exemplified by the clinical lead immunosuppressant T20K.^24^ With growing plant genetic resources^25^ and recent advances in identification of precursor peptides and biosynthetic enzymes involved in plant RiPP biosynthesis, the field of plant RiPPs is primed for an expansion of plant RiPP chemistry and enzymology that will further advance research about their agricultural and therapeutic applications. This review aims to give an overview of plant RiPP chemistry, biosynthesis, and bioactivity while also recommending nomenclature for the recent discovery of side-chain-macrocyclic RiPPs produced by BURP-domain peptide cyclases.

In this review, we will first introduce plant peptide chemistry to highlight characterized ribosomal peptides with post-translational modifications. We will then give insights into plant RiPP biosynthetic trends based on current biochemical knowledge (Section 2). Next, plant RiPP classes will be reviewed in structure, biosynthesis, and bioactivity (Section 3). We summarize the distribution of these plant RiPPs across vascular plants in Section 5. Finally, discovery approaches for plant RiPPs are described (Section 6) and we look ahead in future research directions of plant RiPPs (Section 7). We also review biosynthetically undefined peptides derived from plants (Section 4). This review has a particular focus on BURP-domain-derived RiPPs due to their recent biosynthetic discovery. For the other plant peptide classes, we further recommend excellent reviews discussed in their respective sections.

## 2. Plant peptide chemistry and biosynthesis

To date, over 1500 modified peptides have been isolated from plants.^3,18,26,27^ Detailed biosynthetic studies have firmly established the biosynthetic basis for many of these peptides, allowing for classification based on both structure and construction (Fig. 1B). The plant itself is typically responsible for biosynthesis of the RiPP, which is contrary with other sessile organisms like sponges that rely on a complex microbiome to produce the observed bioactive metabolite.^28–30^ There are notable exceptions to this trend in plants, such as the macrocyclic depsipeptide FR900359,^31^ which is produced by the endosymbiotic bacterium *Candidatus Burkholderia crenata* in the leaves of the plant *Ardisia crenata*. FR900359 is a potent inhibitor of the Gq subfamily of G protein signaling, a novel therapeutic target for treating asthma, inflammation, and cancer.^32–35^ Other peptidic natural products derived from plant sources such as the bouvardins still await biosynthetic classification (Fig. 1B and Section 4).

Plant RiPPs have a wide range in size, from four amino acids (cyclopeptide alkaloids)^36^ up to 66 amino acids (cysteine-rich peptides)^37^ or 430 Da to 7 kDa (Fig. 1B). A common structural feature of plant peptide natural products is macrocyclization. Macrocyclic plant RiPPs can be constructed in a variety of ways: head-to-tail (cyclotide), side-chain-to-side-chain (cyclopeptide alkaloids) or side-chain-to-backbone (lyciumins). Below we describe important features of plant RiPP pathways following general RiPP biosynthetic order of precursor peptide formation, post-translational modification, and proteolysis.

### 2.1. Precursor peptides

As characteristic of RiPPs, genetically encoded precursor peptides serve as the substrate for the biosynthetic pathways. Precursor peptide sequences can often be subdivided by their functional role. Since many contrasting definitions have been used for plant precursor peptide organization over the past decades, we propose the following in addition to accepted RiPP precursor peptide definitions:^1,2^

- Leader (peptide) is the non-repeating N-terminal portion of the precursor peptide following the signal sequence (if a signal peptide is present) before the first N-terminal core peptide.
- Core (peptide) is a sequence of the precursor peptide which is transformed to the RiPP natural product.
- Recognition sequence is the repeating unit demarcating core sequences in multi-core precursor peptides.
- Follower (peptide) is the non-repeating C-terminal portion of the peptide following the core sequence.

Leader, follower and recognition sequences often function as regions for interactions with PTM enzymes or domains and proteases in RiPP pathways.^1,2^ The presence of leader peptide, follower peptides, or recognition sequences is not a requirement for plant RiPP precursor peptides.

Plant RiPPs can be derived from single-core and multi-core precursor peptides (Fig. 1C). Most plant RiPP classes have precursor peptides that have been characterized to include more than one core peptide in sequence repeats, known as multi-core precursor peptides (Table 1).^38^ For example, two of the originally reported cyclotide precursor peptides (Oak2, Oak4) from *Oldenlandia affinis* include 2 or 3 core peptides, respectively, but precursor peptides with only one cyclotide core peptide (Oak1, Oak3) were reported from the same plant.^4^ Other multi-core precursor peptides have been described for orbitides,^39^ linear RiPPs,^11^ several cysteine-rich peptides,^40–42^ lyciumins,^17^ moroidins^19^ and cyclopeptide alkaloids^18,20^ (Table 1). Multi-core precursor peptides are also in biosynthetic pathways of fungal RiPPs such as dikaritins^43–46^ and borosins,^47,48^ and cyanobacterial RiPPs such as cyanobactins^49,50^ and microviridins.^51^ A multi-core precursor in plant RiPPs could have the advantage over a single-core precursor in that RiPP production could be increased faster and more efficient, *i.e.* transcription and translation of one precursor gene can yield multiple plant RiPP products. The evolution of multi-core precursors has been hypothesized to occur through internal gene duplication and neofunctionalization in the form of core peptide diversification, as observed for squash trypsin inhibitor cyclotides.^5^ Many precursor peptides from plant RiPP pathways are above 100 amino acids in length (Table 1 and Fig. 1D) and therefore larger than typical precursor peptides from bacterial RiPPs.^1,2^ The large size of plant precursor peptides arises for several reasons: (a) plant RiPP precursor peptides often include signal peptides for processing through the secretory pathway in plant cells, (b) they can include multiple core peptides,^4,5,17^ (c) core peptides have evolved into storage proteins^22,52,53^ and (d) precursor peptides can be fused to catalytic domains.^18,54^ In some cases, the large size of plant RiPP precursors has been hypothesized to be a result of cyclic peptide motifs ‘hijacking’ plant proteins and their processing enzymes for biosynthesis.^52^ Such cyclic peptide motifs can function as stand-alone inhibitor protein loops like in Bowman–Birk-inhibitors^55^ or serve as RiPP core peptides for post-translational modification and proteolysis during cyclic peptide biosynthesis.^6^

### 2.2. Post-translational modification

**Fig. 2:**
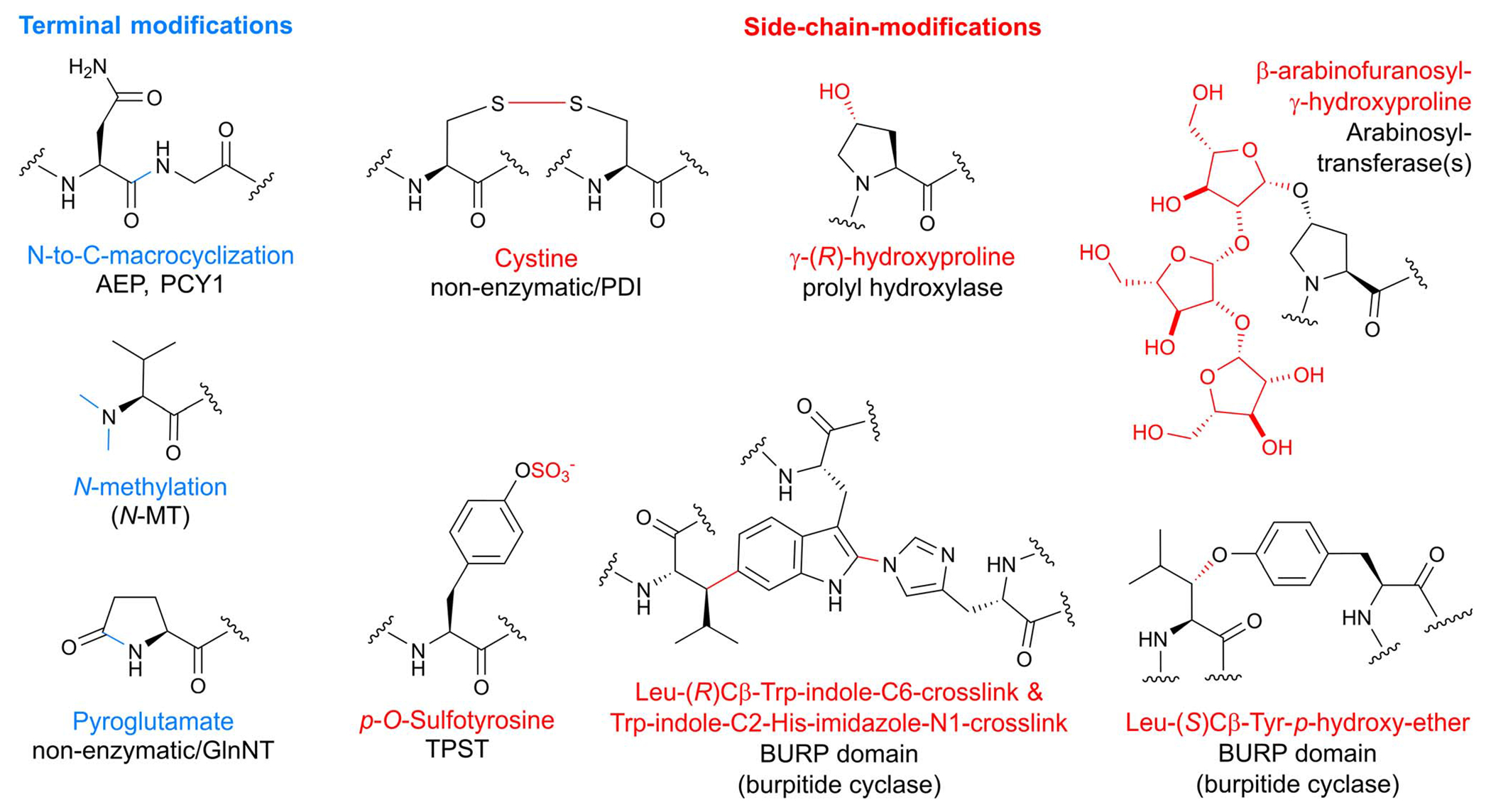
Post-translational modifications in plant RiPPs. Terminal modifications are in blue and side-chain-modifications are shown in red. Abbreviations: AEP – asparaginyl endopeptidase, PCY1 – peptide cyclase 1, *N*-MT – *N*-methyltransferase, GlnNT – glutamine aminotransferase, PDI – protein disulfide isomerase, TPST – tyrosylprotein sulfotransferase.

Bacterial RiPPs are often heavily modified to contain many non-proteogenic amino acids, whereas plant RiPPs are generally comprised of proteinogenic amino acids with only a limited number of post-translationally modified amino acids. Plant RiPP PTMs can be differentiated into terminal modifications such as N-to-C-macrocyclization or pyroglutamate formation and into side-chain-modifications such as crosslinking of tryptophan and tyrosine side chains with other amino acids (Fig. 2). Multiple plant RiPP classes feature PTMs common to eukaryotic proteins, including cystines, pyroglutamate, tyrosine sulfation, and proline hydroxylation.^56^ We rationalize the inclusion of common protein PTMs in plant RiPPs because of the general definition of RiPPs as ribosomal peptides with a post-translational modification and an altered structure–activity-relationship (*e.g.* a change in biological target affinity, stability, conformation, charge state) of the unmodified ribosomal peptide through the PTM. For example, cystines were not detailed in seminal RiPP reviews as PTMs defining a ribosomal peptide as post-translationally modified, but one of these reviews mentions ribosomal peptides with disulfide bonds, so called cysteine-rich peptides (CRPs), following RiPP biosynthetic logic.^1,2^ We suggest including cystines as RiPP-defining PTMs given their nature as a covalent precursor peptide modification and their impact on peptide SAR (Section 3.4). Plant RiPPs with cystines are cyclotides, PawS-derived peptides, and CRPs. In contrast to established RiPP-defining PTMs which are only formed enzymatically, cystines can form non-enzymatically through oxidative folding^57,58^ or enzymatically by protein disulfide isomerases.^59^

Plant RiPP biosynthesis can occur through a split pathway or an autocatalytic (fused) pathway (Fig. 1E). Most plant RiPPs such as cyclotides, orbitides, PawS-derived peptides and cyclopeptide alkaloids are derived from precursor peptides, which are expressed as separate proteins from their PTM enzymes (Table 1). Recently, several BURP-domain-containing proteins (Section 3.6) have been characterized which can catalyze macrocyclic PTMs in core peptides within the same protein as the cyclase domain. This discovery showed that plant RiPP biosynthesis can involve autocatalysis,^18^ a biochemical mechanism first observed for peptide-*N*-methyltransferases of fungal RiPP pathways in borosin biosynthesis.^60,61^

### 2.3. Proteolysis

Plant RiPP biosynthesis utilizes protein processing enzymes found in secretory pathways. For example, the biosynthesis of cyclotides involves asparaginyl endopeptidases (AEP), proteolytic enzymes often involved in processing vacuolar proteins like vacuolar storage protein albumins.^62,63^ Precursor peptides with albumin domains are indeed observed for head-to-tail-macrocyclic plant RiPPs PawS-derived peptides, PawL-type orbitides, and cyclotides. The genetic, structural, and biochemical connection of plant RiPP chemistry to larger plant proteins indicates evolutionary roots of some plant RiPPs from functional motifs in such proteins.^52,55^

## 3. Plant RiPP classes

### 3.1. Cyclotides

**Fig. 3:**
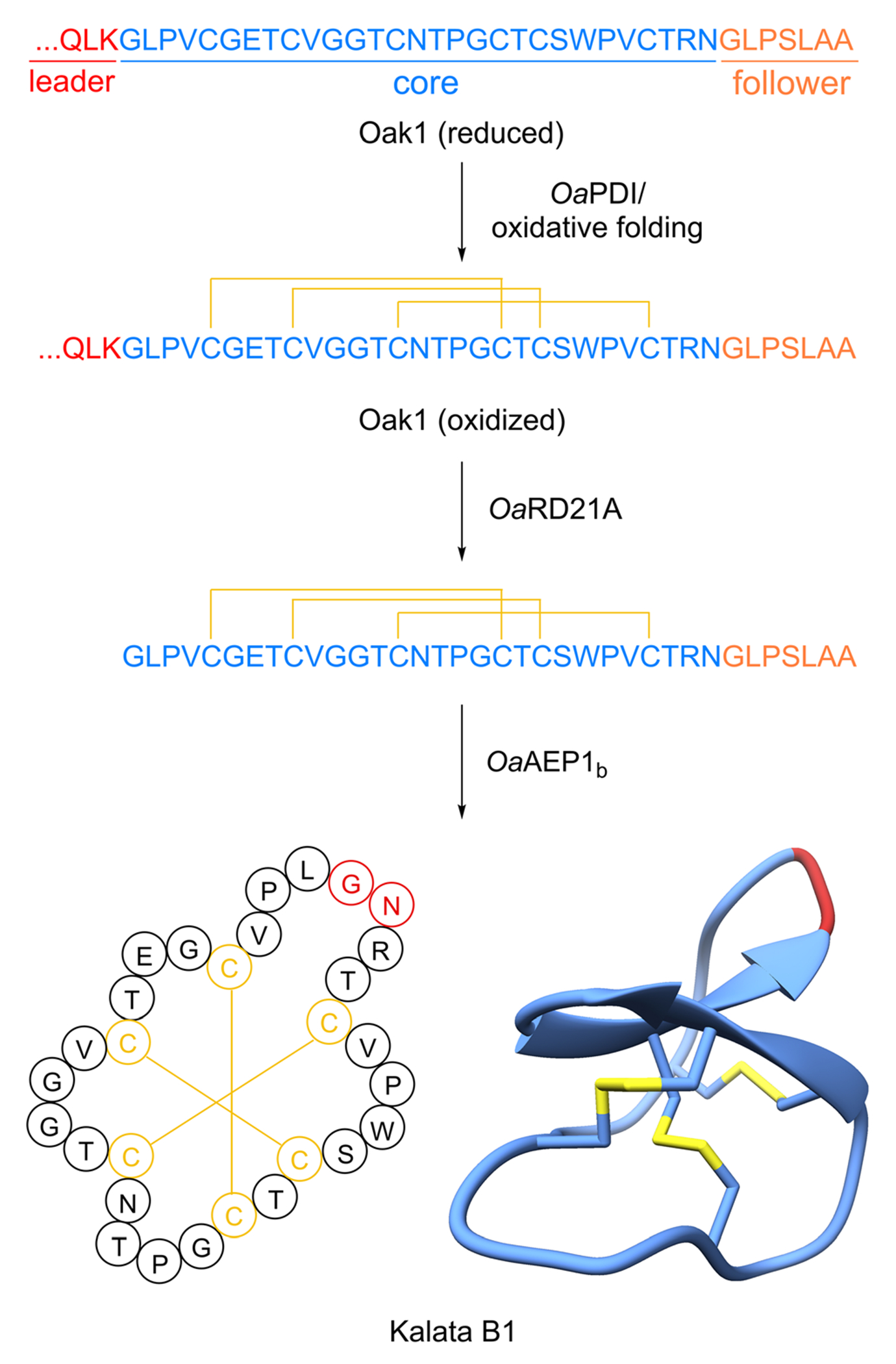
Biosynthesis of cyclotide kalata B1. PDB accession: 1NB1.

Cyclotides are defined by a head-to-tail-macrocyclization and the presence of a cystine knot motif in their structure (Fig. 3). The founding member kalata B1 was discovered as an oxytocic agent in extracts of the Congolese plant *Oldenlandia affinis*, which is used as an herbal medicine to facilitate child birth during labor.^80–83^ Since the discovery of *O. affinis* cyclotides, many other cyclotides have been characterized with 761 cyclotides documented in the cyclic plant peptide database CyBase^26,84^ that establishes cyclotides as the largest plant RiPP family to date. For more details about cyclotides, we direct readers to several dedicated reviews.^85,86^.

#### 3.1.1 Structure.

Cyclotides are 28–37 amino acid-long, head-to-tail-macrocyclic plant peptides with a ‘cyclic cystine knot’-motif formed by three disulfide bonds^87^ (Fig. 3). The characteristic macrocyclic structure of kalata B1 was confirmed by NMR analysis in 1995 ^88^ and the name cyclotide and cyclic cystine knot were subsequently introduced.^87^ Kalata B1 and other cyclotide structures were further confirmed *via* synthetic studies.^89^ Notably, the cystine knot of cyclotides was previously characterized in knottin plant peptides which have no head-to-tail-macrocyclization.^90^ Cyclotides have high stability against heat and enzymatic and chemical degradation.^91^ Three subgroups of cyclotides have been defined based on primary and secondary structure: bracelet cyclotides have an all-*trans* backbone, Möbius cyclotides have a *cis*-proline residue, and trypsin inhibitor-type cyclotides have high sequence similarity to knottin-type trypsin inhibitors. An additional proposed subgroup are lysine-rich cyclotides.^86,87,92^

#### 3.1.2 Biosynthesis.

The ribosomal biosynthesis of cyclotides was first established in kalata B1.^4^ In 2001, the Craik and Anderson labs examined the mRNA of *Oldenlandia affinis* and identified a cDNA termed *Oak1* that contained a core peptide sequence that perfectly matched kalata B1 (Fig. 3). The clone possessed an N-terminal endoplasmic reticulum signal sequence followed by an N-terminal leader peptide. Three additional clones, termed *Oak2–4*, were also found to code for alternative cyclotides. Notably, *Oak2* and *Oak4* possessed two and three separate core peptides, respectively (Fig. 1C). Each core was separated by a conserved N-terminal recognition sequence. *Oak1–4* also contains a conserved C-terminal follower peptide after the core peptide sequence essential for processing. Shortly thereafter, additional cyclotide precursor peptides were discovered from different plants, each displaying a similar arrangement of conserved N-terminal leader peptide or recognition sequence, core peptide, and short C-terminal follower peptide.^93–95^ Cyclotide precursor peptides range from one to seven core peptide sequences.^5^ The core peptides usually start with a glycine at the N-terminus, end with an asparagine or aspartate at the C-terminus, and are flanked by N-terminal and C-terminal recognition sequences.^4,86,93^ Cyclotide precursor peptides are mostly stand-alone precursor peptides, however a precursor peptide from *Clitoria ternatea* (Fabaceae) includes a proalbumin subunit.^96,97^

Once the signal sequence is cleaved, it is thought the disulfide bond formation is the first step of the biosynthesis. The cyclic cystine knot is proposed to be formed by a protein disulfide isomerase in the secretory pathway^59^ (Fig. 3). Oxidative folding studies on kalata B1 core peptide determined that the cystine knot can also form correctly *via* a non-enzymatic pathway in the presence of reducing agents such as glutahione.^57^ Next, the precursor peptides need to be processed to expose the primary amine of the N-terminal amino acid, which is typically glycine.^26^ The Craik and Durek Labs used an activity-based isolation approach to identify a papain-like cysteine protease (*Nb*CysP6) from *Nicotiana benthamiana* that could hydrolyze a short kalata B1 precursor peptide (LQLK-kB1) to acyclic kalata B1.^76^ A homologous enzyme identified in the transcriptome of *O. affinis* named *Oa*RD21A was also able to process the kalata B1 precursor peptide. Notably, the kalata B1 core sequence needed to be properly folded with the requisite three disulfide bonds to prevent further degradation by *Oa*RD21A. These experiments with *Oa*RD21A support that the cyclic cystine knot forms first in the pathway.

The final step of the biosynthesis is the N- to C-terminus macrocyclization. The first cyclase was identified by focusing on the cyclotide producer *Clitoria ternatea*. Tam and colleagues conducted an activity-guided fractionation and isolation of an enzyme that could cyclize a short model peptide substrate to produce kalata B1.^74^ Notably, this is not the native cyclotide produced by *C. ternatea*. Using this approach, they identified an asparaginyl endopeptidase (AEP) they called butelase 1 that efficiently cyclized a wide range of peptide substrates to make cyclotides, PawS-derived peptides, and peptides from animals. Detailed substrate scope assays showed this cysteine protease requires a C-terminal NHV motif to cyclize with an N-terminal glycine. Additional cyclizing AEPs were later screened and characterized from other plants. From the kalata B1 producer *Oldenlandia affinis*, the Anderson and Craik labs identified *Oa*AEP1_b_ (Fig. 3).^75^ This enzyme was successfully heterogeneously expressed in *E. coli* and its activity was reconstituted *in vitro*. *Oa*AEP1_b_ efficiently cyclized the kalata B1 precursor peptide along with a variant that lacked the characteristic disulfide bonds of cyclotides. To understand the structural basis that differentiates a normal AEP from a cyclase, detailed structural and mutagenesis studies were undertaken. A crystal structure of *Oa*AEP1 identified a mutation of a single Cys “Gatekeeping” residue to Ala that improves the cyclization *k*_cat_ by 160 fold.^98^ Further studies in AEPs from the garden petunia (*Petunia* × *hybrida*) demonstrated that this Gatekeeping Cys mutation can be combined with swapping a short “marker of ligase activity” (MLA) loop from a robust AEP cyclase into a weak AEP cyclase to dramatically increase cyclase activity.^99^ Moreover, this MLA region can be used diagnostically to predict if a given AEP is a cyclase.

Instead of separate enzymes for N-terminal processing and cyclizations, some cyclotides may employ a bifunctional enzyme. For example, examination of trypsin inhibitor cyclotides from *Momordica cochinchinensis* (MCoTI-I and MCoTI-II) by the Craik and Durek Labs identified an AEP named MCoAEP2.^100^ This enzyme was able to efficiently both cleave the N-terminus of the MCoTI-II precursor peptide to expose the conserved Gly and then cyclize to generate MCoTI-II. These two reactions were pH dependent, so it was hypothesized the cellular trafficking of the precursor peptide into compartments with different pHs may be important in the maturation process.

#### 3.1.3 Bioactivity.

Insecticidal activities of cyclotides have been well characterized and indicate that these plant natural products function endogenously as defense compounds in source plants.^4^ A common mode of action for insecticidal cyclotide activity is the disruption of plasma membranes in insect gut epithelial cells^101^ or in insect cell culture.^102^ In addition, cyclotides have shown molluscicidal activity against the rice pest golden apple snail.^103^ The insecticidal activities of cyclotides have led to development and approval of a cyclotide-based insecticide Sero-X that includes a mixture of cyclotides from the seeds of the legume *Clitoria ternatea*.^22,23^ Sero-X is a sustainable agrochemical pest control agent as it does not show cytotoxicity against pollinators. This success demonstrates the potential of cyclic plant peptides for sustainable agriculture.

Besides agricultural applicability, cyclotides have a broad range of medicinal activities with several pre-clinical and clinical lead structures to treat human diseases. Cyclotides have anthelmintic activity against gastrointestinal nematodes of sheep^104^ and humans.^105^ In addition, cytotoxicity of multiple cyclotides against cancer cells and in *in vivo* cancer models has been reported.^92,106–114^ Several cyclotides have *in vitro* antibacterial activity, yet less activity in mouse models.^89,115–118^ Antiviral activities, such as anti-HIV function, was determined early in the medicinal exploration of cyclotides^119^ and a recent kalata B1 analog, T20K, has reached clinical trials as a immunosuppressive drug candidate for the treatment of multiple sclerosis (MS) based on promising immune modulatory activity in MS mouse models.^24^

The mode of action of cyclotides is based on their stabilized cyclic cystine knot structure and conserved residues that promote binding to therapeutic targets. The conserved residues found in most cyclotides are a positively charged residue and a glutamate. The positively charged residue can promote binding to target proteases, such as trypsin.^120^

### 3.2. Orbitides

**Fig. 4:**
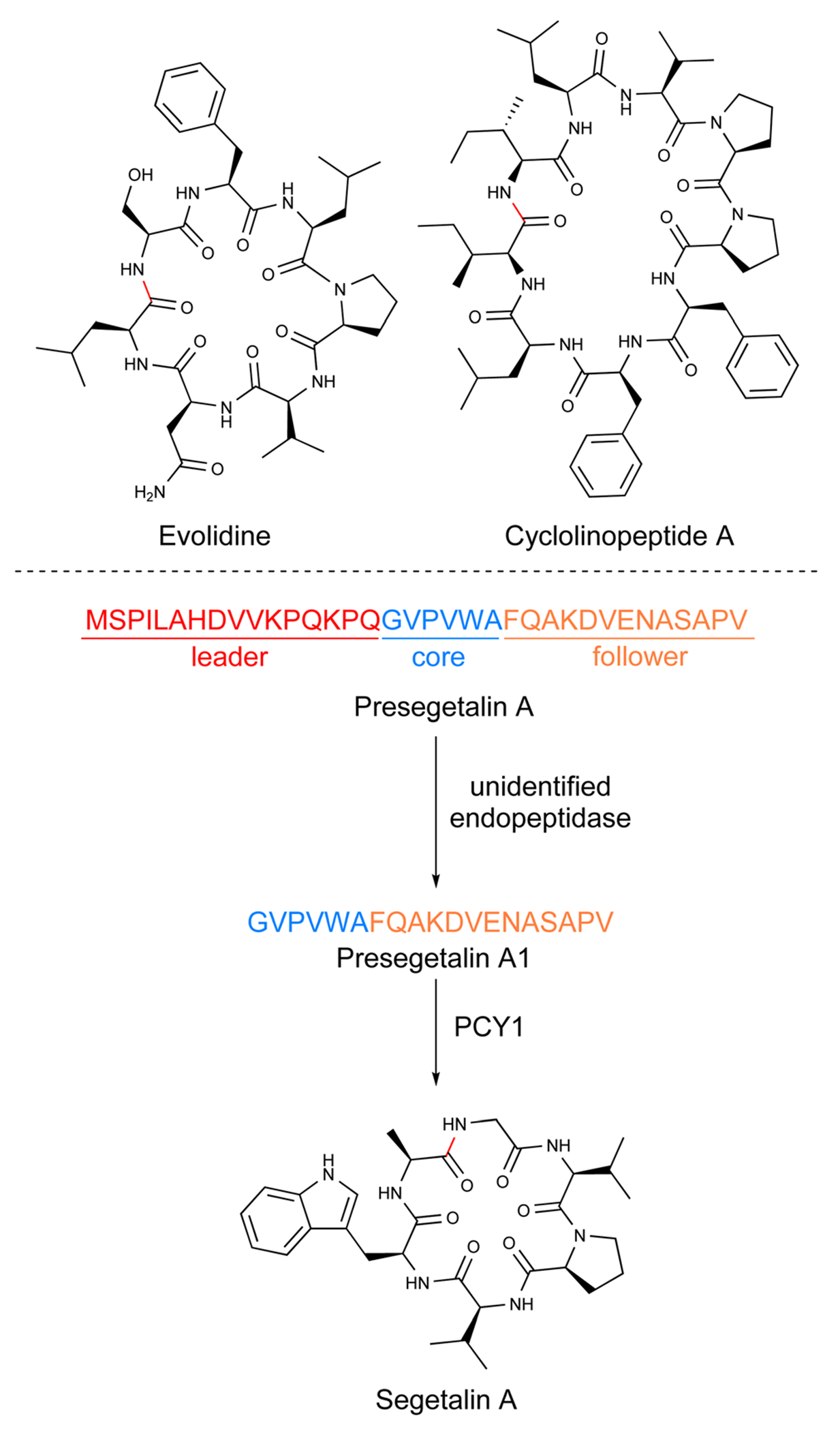
Structures of iconic orbitides and proposed biosynthetic route for segetalin A.

Orbitides are small head-to-tail-macrocyclic RiPPs with no disulfide bond (Fig. 4). They were originally described as Caryophyllaceae-type cyclic peptides because some founding members of these compounds, such as nonapeptide cyclolinopeptide A from flax (*Linum usitatissimum*), were discovered from plants in the pink family.^121^ A wider taxonomic distribution of these compounds was revealed in the same and following decades^122–124^ and the precursor genes of several orbitides were sequenced and confirmed recently which defined them as RiPPs.^6,7,39,77,123,125,126^ In 2013, the name orbitides was proposed for these head-to-tail-cyclic RiPPs without disulfide bonds.^1^ To date, nearly 200 orbitides have been deposited to CyBase^26,84^ and are broadly distributed across plants as they are found in the Asterales, Caryophyllales, Lamiales, Magnoliales, Malpighiales, and Sapindales orders.

#### 3.2.1 Structure.

Orbitides are head-to-tail cyclized peptides between 5 and 16 amino acids in length.^127^ They lack disulfide bonds and are largely devoid of additional post-translational modifications outside a few examples including isoleucine hydroxylation^128^ and *N*-methylation.^129^ The core sequences are highly enriched in uncharged amino acids, with Gly and Pro particularly well represented. Glycine often serves as the N-terminus of the core peptide and, as such, is an important determinant in the macrocyclization reaction.

#### 3.2.2 Biosynthesis.

As orbitides are defined based on structural features and not biosynthetic enzymes, multiple distinct pathways appear to have evolved in plants. The biosynthesis for the Caryophyllaceae-type orbitides is the most well characterized with multiple detailed studies of the orbitide cyclase available. The PawL-type appears to mirror the PawS-derived peptide biosynthesis pathway (Section 3.3), however specific identification of biosynthetic enzymes remains to be completed. Finally, other orbitides may be synthesized by undiscovered pathways.

##### 3.2.2.1 Caryophyllaceae-type.

Based on the early work on cyclotides, it was hypothesized that orbitides would also be produced from a linear precursor peptide. To investigate this, the Covello lab focused on *Vaccaria hispanica* (*Saponaria vaccaria*), a well-known and prolific producer of orbitides.^7^ They examined a cDNA-derived expressed sequence tags library for the presence of core sequences that directly mapped onto the structures of segetalins A through H. A total of 12 core peptide-containing precursor peptides about 35 amino acids in length were discovered. Each precursor contained conserved leader and follower regions flanking the variable core (Fig. 4). While the segetalins are found in precursors with only a single core, other orbitides such as those from *Annona muricata* (soursop),^39^ appear to be biosynthesized from precursor peptides with multiple cores. The segetalin A precursor peptide was expressed in *Vaccaria hispanica* root cultures and detected by LC-MS to confirm the authentic precursor with multiple core peptides. The root extracts additionally processed the linear presegetalin A precursor peptide into the cyclic segetalin A product.^7^

The identification of biosynthetic enzymes of orbitides was done by the Covello lab, who used activity guided fractionation of *V. hispanica* seeds to look for processing of presegetalin A.^77^ Their approach led to two enzyme activities: (1) cleavage of the conserved N-terminus by an unidentified oligopeptidase 1 (OLP1) and (2) cyclization of the presegetalin A1 by peptide cyclase 1 (PCY1)^130^ (Fig. 4). *In vitro* assays of heterologously expressed and purified PCY1 validated that this enzyme was responsible for cyclization to form the final segetalin A product. PCY1 could process a diverse array of orbitide precursor peptides into the cyclic products and was tolerant to mutations in the core region.

While the identity of OLP1 has not been firmly established, PCY1 has been extensively studied. Crystallization by the Nair group revealed PCY1 to be a member of the prolyl oligopeptidase (POP) family of serine enzymes.^130^ Co-crystal structures of PCY1 with the presegetalin A1 substrate along with mutagenesis studies demonstrated that the conserved C-terminal follower peptide was important for recognition by PCY1.^130^ They hypothesized that PCY1 cleaves the core peptide from the follower to form an acyl-enzyme intermediate to facilitate macrocyclization with the N-terminal amine of the core peptide to form the final product. Further crystallographic and mechanistic studies by the Naismith group gave detailed insights into substrate recognition by PCY1.^131^ Notably, their work has characterized substrate tolerance for PCY1 to function as a versatile peptide cyclase.

##### 3.2.2.2 PawL-type.

**Fig. 5:**
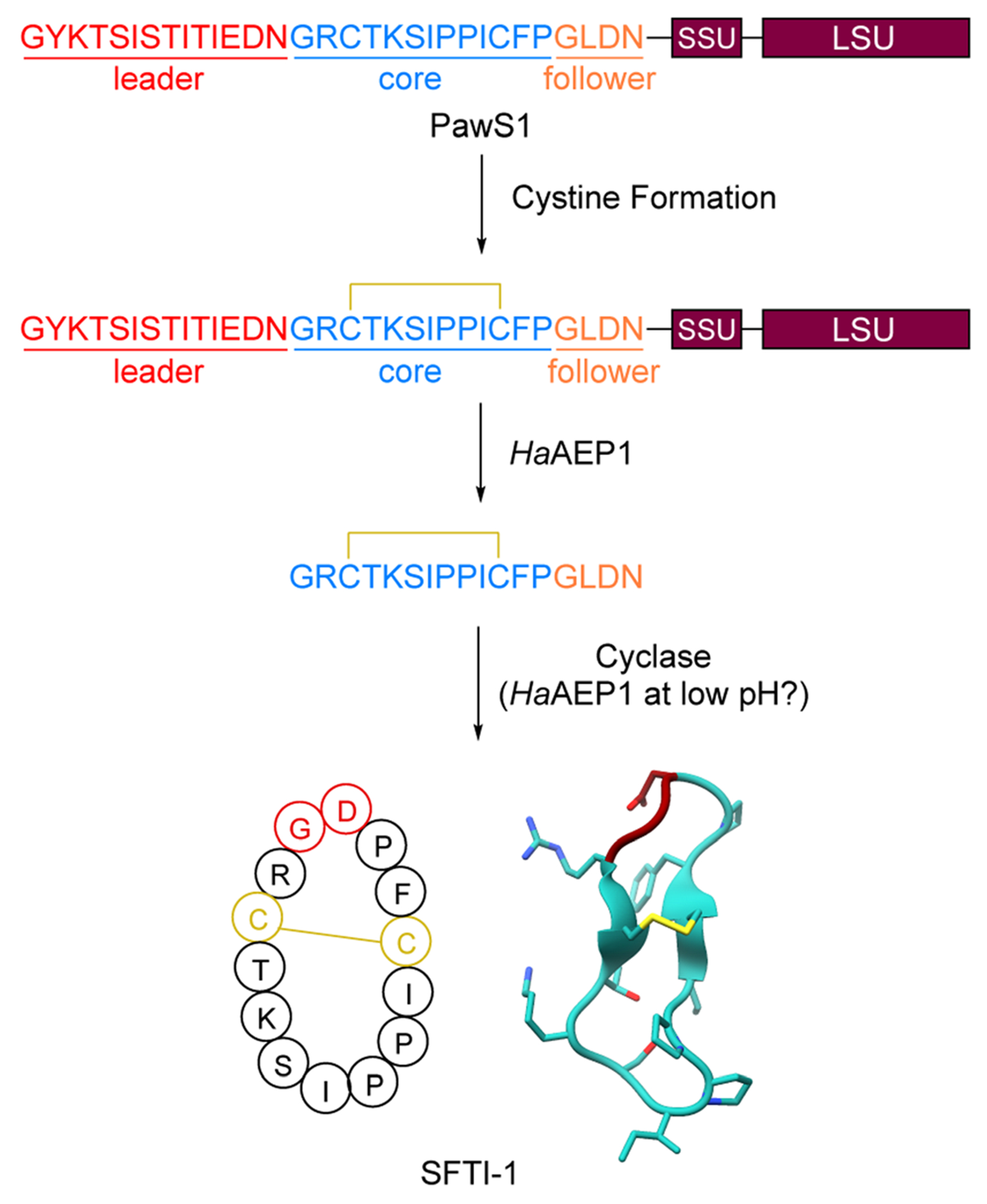
Biosynthesis of SFTI-1. SSU and LSU are small and large subunits, respectively, of albumin domain. PDB accession: 1JBL.

The PawL-type orbitides were discovered using a bioinformatic approach. While characterizing the evolution of the PawS core sequence (Section 3.3), the Mylne group identified the PawS-Like genes (PawL).^124^ Similar to PawS, the PawL core peptide is contained within a two-subunit seed storage albumin (Fig. 5). Post-translational processing by asparaginyl endopeptidases separate the seed storage albumin proteins and cyclize these core sequences. A combination of both transcriptomics and metabolomics validated that these PawL core peptides were processed into cyclic peptides.^123^ The biosynthesis is distinct from the Caryophyllaceae-type orbitides, yet the N- to C- macrocyclic nature and lack of disulfide bonds define PawL-derived peptides as orbitides.

##### 3.2.2.3 Others.

While biosynthetic enzymes have been demonstrated or proposed for the maturation of Caryophyllaceae- and PawL-type orbitides, other orbitides do not have a clearly defined pathway. For example, transcriptomics-based analysis of *Melicope xanthoxyloides* by the Mylne group enabled the discovery of the evolidine precursor peptide.^122^ Characteristic of orbitides, evolidine is a small N- to C-macrocyclic peptide without any disulfide bonds. Like many of the Caryophyllaceae-type orbitides, the evolidine is biosynthesized from a stand-alone precursor peptide with a single core sequence homologous to *Citrus clementina*. However, a lack of conserved proline/alanine in the core region along with differences in the C-termini compared to the precursor peptides from *V. hispanica* led the authors to suggest that a PCY1 homolog is likely not responsible for cyclization of the core sequences, and the cyclase for evolidine remains to be determined.^122^

Similarly, the precursor peptides in *Linum usitatissimum* (flax) are distinct from those processed by PCY1. *L. usitatissimum* is a prolific producer of orbitides, specifically cyclolinopeptides/linusorbs. Efforts from the Reaney group to link the isolated orbitides to precursor peptides have identified genes that encode for multiple core sequences flanked by conserved recognition sequences.^125,132,133^ A recent bioinformatic study has explored these results to identify five separate precursors peptides from *L. usitatissimum*, along with 25 additional linusorb-like domains, that have features consistent with orbitide precursor peptides;^8^ a biosynthetic route remains to be elucidated for these orbitides.

#### 3.2.3 Bioactivity.

The physiological function of orbitides in plants is unknown, yet they have demonstrated a wide range of biological activities. In the 1990s, studies completed with cyclolinopeptide A ([1–9-NαC]-linusorb B3) to investigate its immunosuppressive activity found it was comparable to the clinically approved cyclosporine A in mouse models.^134^ Detailed mechanistic studies later indicated that cyclolinopeptide A inhibits peptidyl-prolyl *cis–trans* isomerase (PPIase) that has the downstream effect of blocking the transcription of interleukin-2 and other cytokines.^135,136^ Other orbitides, hymenistatin I^137^ and cycloleonurinin,^138^ were also found to be immunosuppressive, with the latter demonstrating inhibition of human peripheral-blood lymphocytes response (IC_50_ of 28 ng mL^−1^).

The segetalin series of orbitides were originally discovered based on their estrogen-like bioactivity. The seeds from *V. hispanica* (*Vaccaria segetalis*) have been traditionally used to activate blood flow, promote milk secretion, and treat amenorrhea, and breast infections. Using these purported effects as a guide, segetalin A and B were isolated.^139,140^ Subsequent bioactivity testing of these two orbitides demonstrated a positive effect on rat uterine weight and contraction strength, further supporting their estrogen-like effects. Another study in 1997 found that two new segetalins (G and H) also isolated from *V. hispanica* possessed estrogen-like activity due to their ability to increase uterus growth in rats.^141^

Numerous orbitides have been shown to have anti-inflammatory activity.^142,143^ Of particular note are the cyclomontanin series with cyclomontanin A active in anti-inflammatory activity assays at 1 and 3 μg mL^−1^.^143^ Another recent study on linusorbs (flaxseed orbitides) showed that these orbitides can inhibit the NF-κB pathway and production of pro-inflammatory cytokines at concentrations ranging from 1–4 μM.^144^

Orbitides have also been investigated for anticancer properties. Glaucacyclopeptide B and cherimolacyclopeptide G, were found to be cytotoxic against a nasopharyngeal carcinoma cell line with IC_50_ values of 16.5 μM and 0.52 μM, respectively.^145^ Further examples of cytotoxic orbitides including yunnanin A and yunnanin C showed cytotoxicity with IC_50_ values of 2.1 and 2.2 μg mL^−1^, respectively.^146^ Other orbitides such as [1–9-NαC]-linusorb (LO) B2 and [1–9-NαC]-linusorb B3 (cyclolinopeptide A) demonstrated anti-proliferative properties by the reduction of cancer cell viability and increased apoptosis, and induction of cell cycle arrest.^147,148^ A later study found that linusorb B3 modulated the expression of apoptosis related genes and suppression of cell motility by inhibiting actin polymerization in glioblastoma cells.^149^ Numerous other bioactivity studies have been completed with orbitides and variety of properties relevant to human health have been observed including vasorelaxant activity (segetalin F–H),^150^ moderate antibacterial activity (evolidine),^142^ and weak antiplasmodial activity (ribifolin).^151^

### 3.3. PawS-derived peptides

PawS-derived peptides (PDPs) are head-to-tail-macrocyclic RiPPs with one disulfide bond. The founding member of the PDPs, sunflower trypsin inhibitor (SFTI-1 Fig. 5), was discovered in 1999 from sunflower seeds (*Helianthus annus*).^152^ Additional isolation and genetic mining efforts have revealed around 20 total PDPs.^52,124,153^ These natural products do not appear to be widespread in plants and are largely localized around species phylogenetically related to *H. annus*.^124^ We recommend a review that focuses on SFTI-1 with many details on the extensive engineering efforts of SFTI-1 ^154^ and another review that discusses PDPs more broadly^53^ for further reading.

#### 3.3.1 Structure.

PDPs are head-to-tail-macrocyclized peptides containing a single disulfide bond. All cyclic PDPs to date are formed between an N-terminal Gly and a C-terminal Asp (Fig. 5). While they share conceptual similarities to cyclotides, they are distinct in that they are smaller than cyclotides (12–18 amino acids) and contain only one disulfide bond compared to the three found in cyclotides. Many PDPs are rich in proline and contain a tandem *cis* and *trans* proline pair. PDPs often possess a Bowman–Birk inhibitor^155^ sequence loop mimic that is responsible for the observed trypsin inhibition bioactivity.^124^

#### 3.3.2 Biosynthesis.

By searching for the amino acid sequence of SFTI-1 within *H. annus* cDNA-derived expression sequence tags, Mylne *et al.* were able to identify a region within a protein they termed PawS1 (preproalbumin with SFTI-1) that was an exact match to the SFTI-1 sequence.^52^ PawS1 is a small 151-amino acid protein that contains three features: an ER signal peptide, the SFTI-1 precursor peptide, and a 2S seed albumin (Fig. 5). Subsequent NMR studies on PawS1 confirmed this structural organization.^156^ Seed albumins are used as a nutrient source during development.^157,158^ Bioinformatic searching revealed a second gene termed *pawS2* with the same structure that also matched the same tripartite organization. Mass spectrometric analysis confirmed that the SFTI-Like1 precursor peptide of PawS2 matured into a cyclic peptide like SFTI-1. Using an *A. thaliana* expression system, the authors demonstrated that asparaginyl endopeptidases mature PawS1 into both albumin and SFTI-1. Notably, the native *A. thaliana* AEPs could accomplish the SFTI-1 production and a specialized AEP is not essential.

Additional work from the Mylne and Craik labs further dissected the maturation process of SFTI-1.^159^ Examination of the *H. annus* transcriptome revealed at least five AEPs, and one was particularly upregulated, *Ha*AEP1. *In vitro* reconstitution of *Ha*AEP1 showed hydrolysis after Asn residues to generate a SFTI-1+Follower peptide, but no cyclization (Fig. 5). While the AEP responsible for cyclizing SFTI-1 remained elusive in *H. annus*, the authors also screened other known AEPs from different organisms. Notably, the AEP from *Canavalia ensiformis* (jack bean) termed *Ce*AEP1 was able to fully process a 25-amino acid peptide into cyclic SFTI-1. *Ce*AEP1 acts first as a typical AEP peptidase to trim the peptide to reveal the N-terminal Gly and then as a macrocyclase to form the Gly1-Asp14 peptide bond. While *Ce*AEP1 only forms the cyclic SFTI-1 product 15% of the time, the acyclic SFTI-1 is rapidly degraded *in vivo*. It remains unclear whether the authentic *H. annus* cyclase would have similar cyclization efficiency.

Later studies examined processing of the full length PawS1 protein.^156^ As before, *Ha*AEP1 was able to function as a protease and cleave both the proalbumin and liberate a linear SFTI-1 containing peptide. More recently, a crystal structure of *Ha*AEP1 and subsequent characterization revealed that the maturation process of *Ha*AEP1 and assay conditions strongly influence cyclization activity.^160^ When *Ha*AEP1 is purified as a zymogen, matured by autoactivation at pH 4.0, and assayed at pH 6.5, it is able to catalyze the formation of cyclic SFTI-1 (Fig. 5).^160^ This pH dependent cyclization appears to be a conserved feature of AEPs as it was observed in AEPs from other organisms.

#### 3.3.3. Bioactivity.

SFTI-1 is a potent trypsin inhibitor with a *K_i_* of 0.1 nM.^152^ Initial isolation and co-crystallization with bovine trypsin revealed binding in the active site similar to the Bowman–Birk inhibitor family of natural products.^152^ Specifically, the lysine of SFTI-1 fits into the P1 site of bovine trypsin and adopts a conformation favorable for hydrolysis. Upon cleavage of the labile Lys5-Ser6 bond of SFTI-1 by trypsin, cyclization of the acyl-enzyme intermediate to re-form intact SFTI-1 outcompetes hydrolysis.^154,161^ To evaluate the physiological function of PDPs, bioactivity assays of SFTI-1 with gut extracts of the cotton bollworm (*Helicoverpa armigera*) also showed inhibition indicating that these natural products may serve to protect the plant from insect predation.^124^

### 3.4. Cysteine-rich peptides

**Fig. 6:**
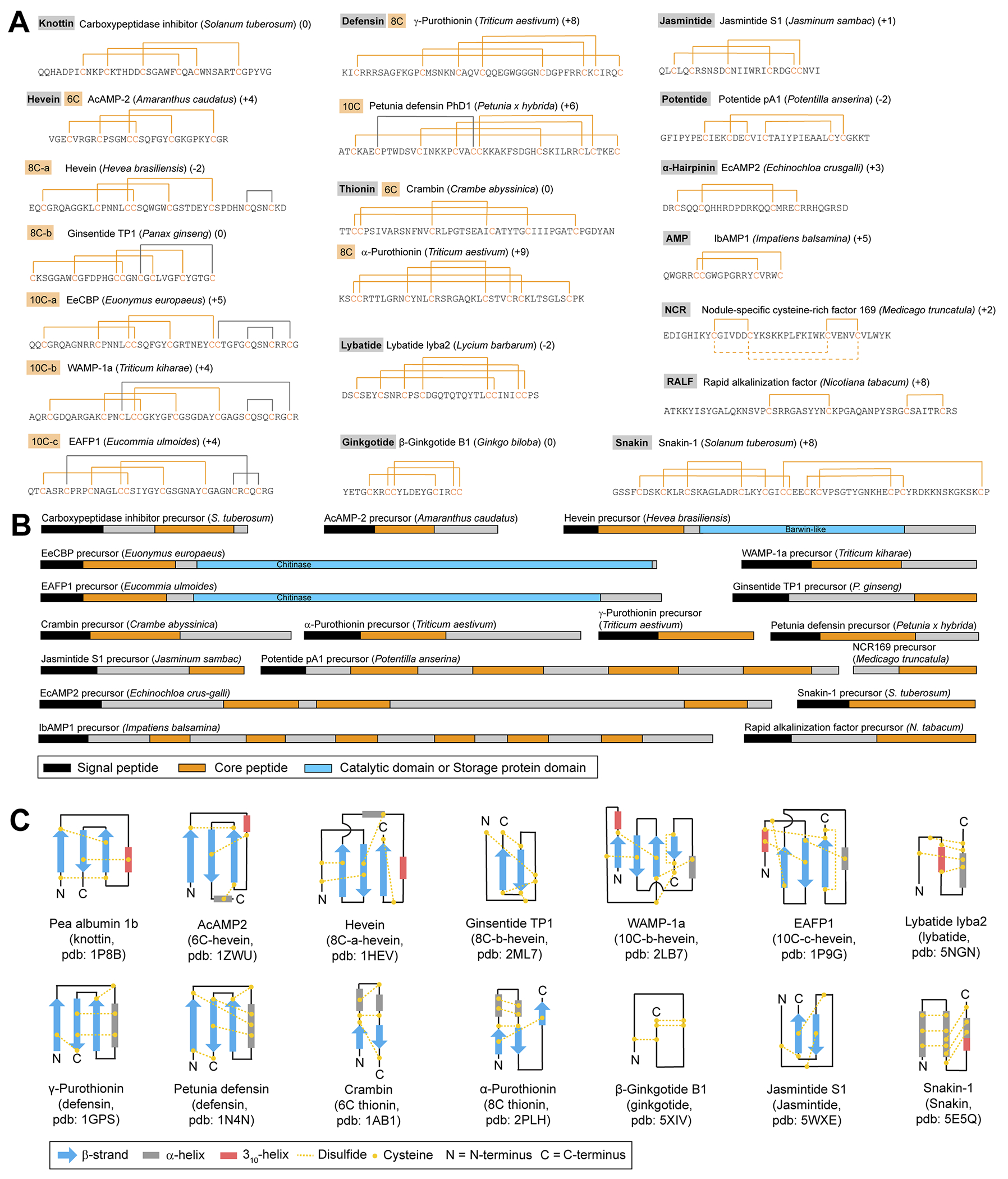
Chemical structures and precursor peptides of cysteine-rich plant peptides. (A) Sequences and disulfide bond patterns of representatives of CRP subclasses. Class-defining DSBs and cysteines are highlighted in orange. Solid lines in gray are non-class-defining DSBs to the CRP. Peptide net charges are shown behind species names. (B) Precursor peptides of CRP subclass representatives. (C) Topology diagrams of CRP subclass representatives based on experimentally determined structures.

In addition to cyclotides, plants produce many peptides without head-to-tail-macrocyclization, but with multiple cysteines that engage in macrocyclization *via* disulfide bonds. The classification of cysteine-rich peptides (CRPs) is historically based on activity (antimicrobial peptides, defensins, rapid alkalinization factors), structure (knottins, thionins, snakins), or chemotaxonomy of founding members (heveins). Given current knowledge of cysteine-rich peptide (CRP) chemistry and biosynthesis, the most applicable classification scheme for CRPs is the number and pattern of disulfide bonds, which translates into distinct structures (Fig. 6). CRP precursor peptide sequences can differ significantly in size and domain structure within individual classes so that core peptide sequence and its cysteine pattern appear to be the best features for CRP prediction (Fig. 6). In addition, the net charge of a core peptide sequence is often considered for CRP characterization (Fig. 6A). Multiple excellent reviews have been published for CRPs.^162–164^

#### 3.4.1 Structure.

The number of disulfide bonds in plant CRPs ranges from two (rapid alkalinization factors) to six (snakins), and from 18 to >50 amino acids total. CRPs discussed in this review include the following subclasses: knottins,^90,165,166^ thionins,^167^ defensins,^67,168^ snakins,^37,169^ heveins,^170,171^ α-hairpinins,^172^ potentides,^40^ β-ginkgotides,^173^ jasmintides,^174^ lybatides,^175^ impatiens AMPs,^42^ nodule cysteine-rich peptides,^176^ and rapid alkalinization factors.^73^ Another antimicrobial peptide class which is often included in CRP classification is lipid-transfer proteins.^162,177^ Lipid-transfer proteins are 9–10.5 kDa and more appropriately classified as a post-translationally modified protein, as they exceed the upper limit of 10 kDa for RiPPs.^1^

Knottins feature a cystine knot motif, also called inhibitor cystine knot^178^ or knottin fold,^166^ based on three cystines in cyclotides: two disulfide bonds form a loop and the third one passes through the first two disulfides, producing a knot with five loops between the disulfides in the RiPP.^90^ The core peptide cysteine pattern of knottins is C(1)X_3–6_C(2)X_5_C(3)X_3–5_C(4)X_1–2_C(5)X_5–6_C(6), where the numbers in parentheses indicate the order of cysteines in the core and X_*n*_ between Cys indicates n non-cysteine residue(s) between the next cysteine based on characterized CRP structures. The knottin cysteine pattern yields disulfide bonds between C(1)–C(4), C(2)–C(5) and C(3)–C(6) (Fig. 6A). Knottins are 26 to 37 amino acids long (~2.7 to 5 kDa). The founding member of the knottins is the potato carboxypeptidase inhibitor which yielded the first structure of a cyclic cystine knot peptide.^90,179^ The name ‘knottin’ was introduced in a study of a microprotein with knotted cystine topology from *Ecballium elaterium* seeds.^166,180^ Knottins such as trypsin inhibitors from *Momordica cochinchinensis* (MCoTI-3/5/6) can have an N-terminal pyroglutamate derived from an N-terminal glutamine^5^ and they are generally neutral or positively charged. Knottin structures such as pea albumin 1b knottin consist of three antiparallel β-strands and one 3_10_-helix crosslinked by the three disulfide bonds^181^ (Fig. 6C). Knottins have been characterized from Solanaceae,^90,182^ Fabaceae,^181,183,184^ Cucurbitaceae,^120^ Apocynaceae,^185^ Rubiaceae,^186,187^ and Poaceae.^188^

Thionins belong to a group of CRPs termed antimicrobial peptides (AMPs) that are characterized by positive charge and antimicrobial activity due to interaction with negatively charged bacterial membranes.^189^ Thionins are a plant AMP class with three to four disulfide bonds. The core cysteine pattern of thionins with three disulfide bonds (DSBs) is C(1)C(2)X_11_C(3)X_9_C(4)X_5_C(5)X_7_C(6) with DSB between C(1)–C(6), C(2)–C(5) and C(3)–C(4). The core cysteine pattern of thionins with four DSBs is C(1)C(2)X_7_C(3)X_3_C(4)X_8_C(5)X_3_C(6)X_1_C(7)X_7_C(8) with DSBs between C(1)–C(8), C(2)–C(7), C(3)–C(6) and C(4)–C(5) (Fig. 6A). Thionins are 44 to 47 amino acids long (4.7 to 5.3 kDa). In addition, several variants have been identified or predicted that have uneven numbers of cysteines in the core peptides, and it remains unclear how the structure would be affected in this case.^190^ Thionins are generally positively charged with few neutral exceptions.^191^ The first characterized plant thionins were α/β-purothionin isolated from wheat (Poaceae) as agents toxic to baker’s yeast^192,193^ with several others identified from Brassicaceae,^194–196^ Liliaceae,^197^ Papaveraceae,^190^ Ranunculaceae,^198^ and Santalaceae.^199–202^ Thionins share a fold consisting of two antiparallel β-strands and two α-helices stabilized by disulfide bonds (Fig. 6C).^167,193^

Defensins are host defense peptides with four disulfide bonds and general positive charge which have also been called ‘γ-thionins’ in the literature.^67^ Plant defensins belong to the *cis*-defensin class which has two conserved disulfides crosslinking a central β-strand to an α-helix^203^ (Fig. 6C). Plant defensins usually have four DSBs with a cysteine pattern C(1)X_10–11_C(2)X_3–5_C(3)X_3_C(4)X_9–11_C(5)X_4–9_C(6)X_1_C(7)X_2–3_C(8) with DSBs between C(1)–C(8), C(2)–C(5), C(3)–C(6) and C(4)–C(7). PhD1, a *Petunia* defensin, has an additional DSB and a cysteine pattern of C(1)X_3_C(2)X_6_C(3)X_5_C(4)X_2_C(5)C(6)X_9_C(7)X_6_C(8)X_1_C(9)X_3_C(10) with DSBs between C(1)–C(10), C(2)–C(5), C(3)–C(7), C(4)–C(8) and C(6)–C(9) (Fig. 6A). The general size of plant defensins is 45 to 54 amino acids but several representatives with up to 71 amino acids are known.^204,205^ The plant defensin fold contains a β-sheet and an α-helix and is also referred to as a cystine-stabilized αβ-motif^203,206^ (Fig. 6B). The first plant RiPPs recognized as plant defensins were two γ-purothionins that were initially classified as thionins and later structurally differentiated as defensins.^67,168^ To date, plant defensins were characterized from Asteraceae,^207^ Brassicaceae,^208–212^ Caryophyllaceae,^213^ Fabaceae,^213–218^ Oleaceae,^219^ Pentadiplandraceae,^204^ Pinaceae,^220^ Poaceae,^67,168,205,221–223^ Ranunculaceae,^224^ Sapindaceae,^225^ Saxifragaceae,^226^ Solanaceae,^227,228^ and Vitaceae.^229^

Heveins are a diverse class of CRPs which derive their name from the founding member hevein, a wound-induced antifungal peptide from the rubber tree (*Hevea brasiliensis*).^170^ Heveins have three to five cystines with a foundational cysteine pattern C(1)X_4–8_C(2)X_4–6_C(3)C(4)X_4–5_C(5)X_4–7_C(6), connected by the three DSBs *via* C(1)–C(4), C(2)–C(5) and C(3)–C(6). Heveins have a general size of 29 to 45 amino acids (3 to 5 kDa) and are usually positively charged except for a few neutral representatives. Two subclasses of heveins, hevein-type heveins and ginsentides, have eight cysteines (8C-hevein) in the core peptide and thus an additional DSB with variable positioning to the core DSB motif (Fig. 6A). An additional three subclasses of heveins are represented by EeCBP from *Euonymus europaeus*, WAMP-1a from *Triticum kiharae*, and EAFP1 from *Eucommia ulmoides*, which all have ten cysteines (10C-heveins) in their core peptides and form five DSBs. The additional cysteines in 8C- and 10C-heveins are generally located C-terminally of the core hevein cysteine pattern (Fig. 6A). Heveins typically contain an antiparallel β-sheet, a 3_10_-helix, and an α-helix, whereas ginsentides thus far lack helices (Fig. 6C). Hevein CRPs have been characterized from Amaranthaceae,^230–235^ Araliaceae,^65^ Asteraceae,^236^ Cactaceae,^237^ Caryophyllaceae,^238,239^ Celastraceae,^240^ Convolvulaceae,^241^ Cycadaceae,^242^ Eucommiaceae,^54,243^ Ginkgoaceae,^173^ Malvaceae,^244,245^ Nyctaginaceae,^246^ Phytolaccaceae,^247^ Poaceae,^248–250^ and Polygonaceae.^251^

Several CRP subclasses with unique cysteine patterns of three or four DSBs were discovered recently in plants. Lybatides are 32 to 33 amino acid long CRPs (~3.5 kDa) with an 8-cysteine pattern of C(1)X_3_C(2)X_3_C(3)X_2_C(4)X_10_C(5)C(6)X_3_C(7)C(8) forming the four DSBs C(1)–C(6), C(2)–C(8), C(3)–C(7) and C(4)–C(5)^175^ (Fig. 6A). Lybatides were discovered from *Lycium barbarum* (Solanaceae). The structure of lybatide lyba2 revealed a fold with disulfide-stapled helices (Fig. 6C). β-Ginkgotides are 18–20 amino acid-long CRPs (~2.5 kDa) from *Ginkgo biloba* (Ginkgoaceae) with three DSBs of C(1)–C(4), C(2)–C(6) and C(3)–C(5) derived from the cysteine pattern C(1)X_2_C(2)C(3)X_6_C(4)X_2_C(5)C(6) (Fig. 6A). The NMR structure of β-ginkgotide B1 did not show any helices or β-strands^252^ (Fig. 6C). Jasmintides are 27 amino acid long CRPs (3.1 kDa) isolated from *Jasminum sambac* (Oleaceae), with six cysteines and three disulfides between C(1)–C(5), C(2)–C(4) and C(3)–C(6) from cysteine pattern C(1)X_2_C(2) X_5_C(3)X_6_C(4)X_3_C(5)C(6) (Fig. 6A) with more predicted variations.^174^ Two jasmintides have been elucidated by solution NMR that are characterized by two antiparallel β-strands^71,174^ (Fig. 6C). Ajasmintide from *Achyranthes bidentata* was identified with an unusual N-terminal fructosylation and three antiparallel β-strands.^253^ Lastly, a subclass of CRPs from the roots of *Potentilla anserina* has three DSBs called potentides,^40^ characterized by C(1)–C(3), C(2)–C(6), and C(4)–C(5) DSBs in a C(1)X_3_C(2)X_2_C(3)X_2_C(4)X_10_C(5)X_1_C(6) cysteine-pattern (Fig. 6A). Potentides are 35 amino acid long and to date no structure has been determined. While lybatides and β-gingkotides are slightly acidic, jasmintides and potentides are neutral to slightly basic.

Snakins are a CRP subclass that was discovered in the late 1990s from potato. Snakins have 12 cysteines and six disulfide bonds (C(1)–C(7), C(2)–C(5), C(3)–C(4), C(6)–C(12), C(8)–C(11), C(9)–C(10)) in the cysteine pattern C(1)X_3_C(2)X_3_C(3)X_8_C(4)X_3_C(5)X_2_C(6)C(7)X_2_C(8)X_1_C(9)X_11_C(10)X_2_C(11)X_12_C(12).^37^ Snakins are large peptides of 63 to 66 amino acids and are positively charged. The first characterized snakin CRP was snakin-1, with antifungal properties. The structure revealed a fold with multiple DSB-stapled helices^169^ (Fig. 6C).

Finally, plants produce several RiPPs with two DSBs. α-Hairpinins and impatiens AMPs have both two DSBs with the pattern C(1)–C(4), C(2)–C(3) and C(1)–C(3), C(2)–C(4), respectively, from cysteine patterns C(1)X_3_C(2)X_11_C(3)X_3_C(4) and C(1) C(2)X_8_C(3)X_3_C(4), respectively (Fig. 6A)^42,172^ with more predicted variations for α-hairpinins.^254^ α-Hairpinins such as EcAMP2 were discovered from *Zea mays* (Poaceae),^255^ later described from Proteaceae,^254^ are 26–67 amino acids long, and generally positively charged with a few negatively charged exceptions. AMPs from *Impatiens balsamina* are 20 amino acid long, positively charged, and have a C-terminal glutamine-derived pyroglutamate.^42^ Another plant CRP subclass with two DSBs are rapid alkalinization factors (RALFs) (Fig. 6A). RALF from *Nicotiana tabacum* was characterized to have a C(1)–C(2) and C(3)–C(4) DSB pattern in a C(1)X_9_C(2)X_12_C(3)X_5_C(4) cysteine pattern.^73^ RALF peptides are important signaling peptides, and it was discovered based on its activity to alkalinize tobacco cell suspension cultures and inhibit root growth.^73,256^ Nodule-specific cysteine-rich peptides (NCR) of 36–58 amino acids have been characterized from Fabaceae such as *Medicago truncatula* and *Pisum sativum*.^72^ The oxidative folding and solution NMR analysis of the RiPP NCR169 showed that two DSB patterns can form, *i.e.* C(1)–C(2), C(3)–C(4) or C(1)–C(3), C(2)–C(4), from the C(1)X_5_C(2)X_12_C(3)X_4_C(4) cysteine pattern *in vitro*, where the former DSB pattern was found only in the presence of reduced glutathione^176^ (Fig. 6A, solid lines).

#### 3.4.2 Biosynthesis.

Most precursor peptides of CRPs are single-core precursors (Fig. 6B and Table 1) and have an N-terminal signal peptide indicating processing through the secretory pathway. The multi-core exceptions are precursor peptides of potentides, α-hairpinins and AMPs from *Impatiens balsamina*. Several hevein precursor peptides include additional protein domains: the hevein precursor includes a C-terminal Barwin-like domain,^14,257^ whereas the precursor peptides of 10C-heveins from *Eucommia ulmoides* and *Euonymus europaeus* include C-terminal chitinase domains.^16,54^ Thionin precursor peptides contain a C-terminal acidic domain without a known function.^190^ Some CRPs, including several knottins, heveins, and jasmintides, possess an N-terminal pyroglutamate which is derived from an N-terminal core glutamine.

The PTM event in CRPs is the formation of disulfide bonds during the folding process of a core peptide, whereby the peptide sequence and cellular environment dictates folding and disulfide formation. DSBs can form non-enzymatically under increasingly oxidizing conditions of the secretory pathway in plants and enzymatically by protein disulfide isomerases (PDI), which can reshuffle established disulfides, typically from incorrectly formed DSBs to the native DSBs in the peptide. The improvement of CRP disulfide formation was shown *in vitro* on a core peptide substrate of *Amaranthus* α-amylase knottin-type inhibitor (AAI) by PDI DsbC. As for kalata B1 disulfide formation by *Oa*PDI, the folding process towards α-amylase knottin-type inhibitor does not require a PDI, however its inclusion dramatically increases forming a correctly folded RiPP.^58,59^ Transformation of a linear reduced CRP core towards the most thermodynamically stable oxidized (native) structure is generally catalyzed by the presence of a redox pair, such as reduced and oxidized glutathione or cysteine and cystine.^258,259^ Two general oxidative folding pathways have been described for CRPs: scrambled and native folding. In the scrambled pathway, intermediates with non-native DSBs are formed and reshuffled to native DSBs of the native structure. Examples for the scrambled pathway are knottins such as *Amaranthus* α-amylase inhibitor and potato carboxypeptidase inhibitor.^58,259^ In the native folding pathway, intermediates with native DSBs are formed without the need for reshuffling. Examples include *Ecballium elaterium* trypsin inhibitor II (EETI II)^260^ and Momordica charantia trypsin inhibitor MCh-I.^261^ In the knottin EETI-II folding pathway, two intermediates were characterized which were derived through sequential formation of the C(3)–C(6) and then C(2)–C(5) disulfide bridges before the final formation of the C(1)–C(4) disulfide. The investigation of such CRP folding pathways can inform their synthesis from synthetic linear peptide substrates with protection strategies mimicking the native folding pathway of a CRP sub-class.^262^

Another important step in CRP biosynthesis is proteolytic processing of precursor peptides. It is hypothesized that proteolysis occurs after disulfide formation by proteases in the secretory pathway for most of the CRPs. An example is the characterization of a thionin-processing proteinase from barley leaf. The protease responsible for cleavage of an oxidized core peptide in a precursor peptide *in vitro* to release a mature thionin is a subtilisin-like vacuolar protease. The corresponding studies revealed that the thionin precursor was processed in the vacuoles of barley leaves from which the precursor processing protease was isolated.^263,264^ Mature barley thionins have been localized in vacuoles and in cell walls of leaf cells.^265,266^

#### 3.4.3 Bioactivity.

Many cysteine-rich plant peptides are part of the innate immune response of plants against fungal and bacterial pathogens and insect attacks. Knottins are potent inhibitors of fungal and insect proteases,.^267–270^ Several heveins have inhibitory activity against phytopathogenic fungi as well. For instance, hevein has a chitin-binding domain, which can interact with chitin of pathogenic fungi, and further inhibit fungal metalloproteases like chitinases that target plant defense proteins.^232,271^ Thionins also have inhibitory activity against phytopathogenic bacteria and fungi,^266,272,273^ which was first realized when α/β-purothionins were discovered as inhibitory agents of baker’s yeast.^274^ Thionin is hypothesized to target and disrupt negatively charged phospholipids in cell membranes as mode of action.^275^ Plant defensins exhibit antifungal, antibacterial and insecticidal activity,^276–278^ and can inhibit insect α-amylase *in vitro*.^279^ Jasmintides have antifeedant activity against mealworm *Tenebrio molitor*,^174^ and maize α-hairpinins and snakins also showed antibacterial and antifungal activity against phytopathogens.^37,255,280^ The main mode-of-action hypothesis for snakins is non-specific pore-forming activity in tested bacterial and fungal membranes.^281^ This mode-of-action is supported by perforation of fungal biomembranes in the presence of snakin-2.^282^ Full antifungal bioactivity is achieved by enantiomer d-snakin-1 compared to l-snakin-1 indicating a non-chiral mode-of-action such as interaction with negatively charged membrane lipids.^169^ The snakin target interaction for membrane pore formation remains to be determined. Several nodule-specific cysteine-rich peptides function to control bacterial differentiation during symbiosis of nitrogen-fixing bacteria in legume nodules for nitrogen fixation^283–285^ despite having *in vitro* antibacterial activity. For example, NCR247 from *Medicago truncatula* was characterized to have nanomolar affinity to haem and therefore iron sequestration activity in root nodules which triggered uptake of iron in nodule symbiont *Sinorhizobium meliloti*, a cofactor for its nitrogen fixation.^286^ RALF peptides are involved in regulation of plant development and have several cellular effects, including MAP kinase activation and blockage of a proton pump, the latter implicating RALF as an alkalinization factor in tobacco cells. As an alkalinization factor, RALF has been observed to increase the pH that causes an observed stop in root growth.^73^

The endogenous bioactivity against phytopathogens and herbivores from CRP subclasses such as heveins, snakins, knottins, and defensins, has been successfully applied in transgenic plants to generate more disease- and pest-resistant plants.^276,287–290^ In addition, CRPs such as knottins have potential as therapeutics, drug delivery agents, and diagnostics. For example, knottin–drug conjugates targeting integrins have shown potent inhibition of tumor cell proliferation^291^ and knottins have inhibitory activity against cancer-relevant proteases such as matripase-1.^292^ Recently, the knottin excelsatoxin A from nettle *Dendrocnide excelsa* was described as a pain-causing agent. Excelsatoxin A targets a transmembrane protein, TMEM233, that modulates the voltage-gated sodium channel Na_V_1.7 involved in pain sensation.^293,294^ Pain sensations of *Dendrocnide* plants have been described as long-lasting and intense; excelsatoxin A represents a major pain-causing agent from these plants with potential as an analgesic lead structure. Lastly, a mutant form of knottin can be labeled with ^18^F and used in Positron Emission Topography (PET) imaging studies by binding to integrin positive tumors with high affinity.^295^

### 3.5. Linear plant RiPPs

In addition to cyclic RiPPs, multiple linear plant peptides with post-translational modifications are produced by plants. Many of these linear RiPPs have roles in plant development and growth and are often secreted. The most common post-translational modifications in linear plant RiPPs are known from protein biosynthesis:^56^ tyrosine sulfation,^296^ proline hydroxylation, and hydroxyproline arabinosylation (Fig. 2).^297^ We direct readers to dedicated reviews for additional details^296,298,299^ as we discuss them only briefly.

#### 3.5.1 Structure.

**Fig. 7:**
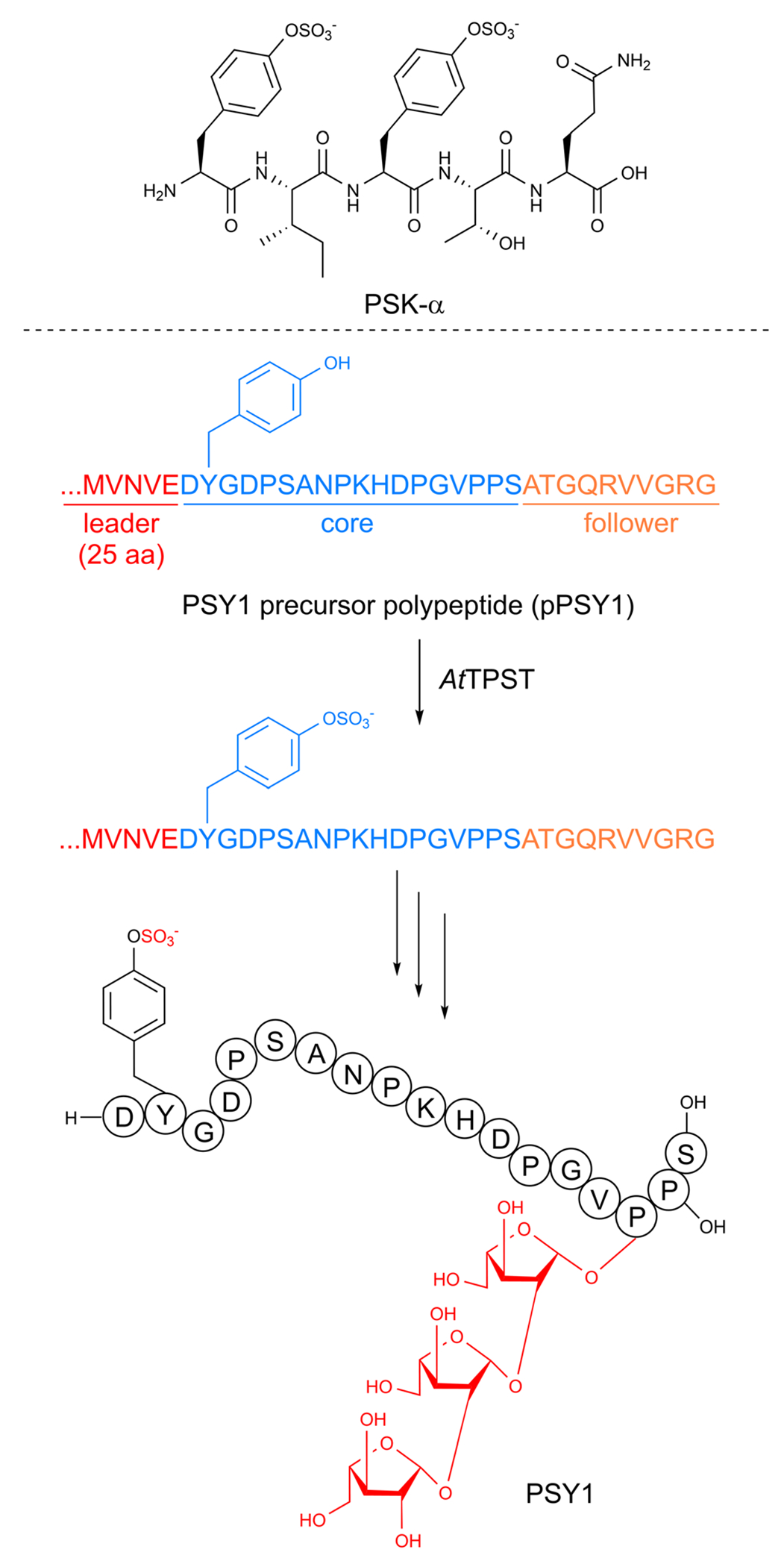
Biosynthesis of linear RiPP PSY1.

The discovery of the first linear plant RiPP was based on a desire to understand the molecular components responsible for cellular division in the conditioned growth media for plant cell culture. The Matsubayashi group conducted activity-guided fractionation from the condition media of *Asparagus officinalis* and identified a five amino acid peptide they named phytosulfokine-α (PSK) as a growth-promoting signaling molecule for *in vitro* asparagus cell culture.^9^ Two of the tyrosines in the peptide were sulfated (Fig. 7) based on sequencing and mass spectrometry analysis. The authors validated PSK as a potent mitogen, stimulating cell growth at nanomolar concentrations, when compared to a synthetic standard. PSK was later isolated from *Oryza sativa*^300^ with its single-core-containing precursor peptide identified shortly thereafter.^301^ Other notable examples of sulfotyrosine-containing linear peptides include Casparian strip integrity factor 1 (CIF1)^302,303^ and root meristem growth factors (RGFs).^304^ Linear plant peptide hormones called systemins were characterized in tobacco and featured a hydroxyproline PTM and *O*-arabinosylation on the hydroxyproline hydroxyl group.^11^

#### 3.5.2 Biosynthesis.

The precursor peptides responsible for linear plant RiPPs are typically around 80–150 amino acids in length and commonly contain only a single core,^301,305,306^ although multi-core examples do exist.^11^ Elucidating the maturation process of these linear RiPPs is challenging as the observed modifications overlap with typical protein PTMs. However, some of the biosynthetic enzymes have been clearly delineated.

While the sulfotransferase reaction is well known to be catalyzed by tyrosylprotein sulfotransferases (TPST) in mice and humans, no enzyme with a high sequence similarity to TPST has been identified in plants.^78^ Therefore, the Matsubayashi group used an enzyme activity-based fractionation approach with the precursor peptide for plant peptide containing sulfated tyrosines 1 (PSY1) (discussed below) as a substrate.^78^ They identified a membrane protein they named *At*TPST that catalyzed the anticipated tyrosine sulfation reaction using 3′-phosphoadenosine 5′-phosphosulfate (PAPS) as the sulfate source (Fig. 7). Subsequent heterologous expression and *in vitro* assays demonstrated that *At*TPST could also utilize the PSK precursor peptide, indicating it may be responsible for the *in vivo* maturation of diverse linear plant RiPPs.

The formation of linear RiPP hydroxyproline and the subsequent addition of three arabinose residues (Fig. 7) has been the focus of recent studies.^79^ The initial hydroxylation is predicted to be installed by a prolyl-4-hydroxylase. Many members of this enzyme family are found in plants but one directly responsible for the hydroxylation in these plant RiPPs has not been demonstrated.^307^ If present, the three arabinoses are likely added in a stepwise order by one enzyme that catalyzes the initial attachment to hydroxyproline and a second that sequentially adds two more.^79^ A candidate for the first step has been identified, but the following arabinose additions have not been defined.

Steps involved in the proteolytic maturation of the core peptide have also been elucidated in several systems. CLE40 is a 12 or 13 amino acid peptide with a single hydroxyproline. Differential expression analysis and *in vitro* assays identified the three subtilisin-like proteases that were able to cleave C-terminal to the core sequence of the precursor peptide.^308^ Notably, the presence of the hydroxyproline residue prevented an internal cleavage of the core sequence. Subtilisin-like proteases were also found to be involved in the maturation of PSK by cleaving three amino acids N-terminal and directly C-terminal to the core peptide. Similar proteases are implicated in production of several other linear plant peptides.

#### 3.5.3 Bioactivity.

In general, these linear plant RiPPs function as important signaling molecules or hormones involved in plant growth and cellular differentiation. PSK was discovered as a component responsible for the conditioned growth media needed for plant cell culture. Using a synthetic standard, the Matsubayashi group validated that PSK was a potent mitogen, simulating cell growth at nanomolar concentrations.^9^ PSK was later isolated from *Oryza sativa* and a similar activity was observed.^300^ A related approach was used to find PSY1, which also promotes cellular growth.^305^ CLV3 suppresses shoot apical meristem cell development and instead upregulates cellular division.^10^ CIF1 peptides are necessary for the formation of the Casparian strip in *Arabidopsis* roots, a structure important for ion homeostasis.^302^ Other peptides are involved in responses to stress such as dehydration,^309^ osmotic stress,^310^ and nitrogen starvation.^311^ Notably, the fundamental importance of these plant peptides has been targeted by pathogens. The sulfated tyrosine containing bacterial RiPP RaxX is thought to mimic PSY1 and serve as a virulence factor.^312^

### 3.6. BURP-domain-derived RiPPs (burpitides)

**Fig. 8:**
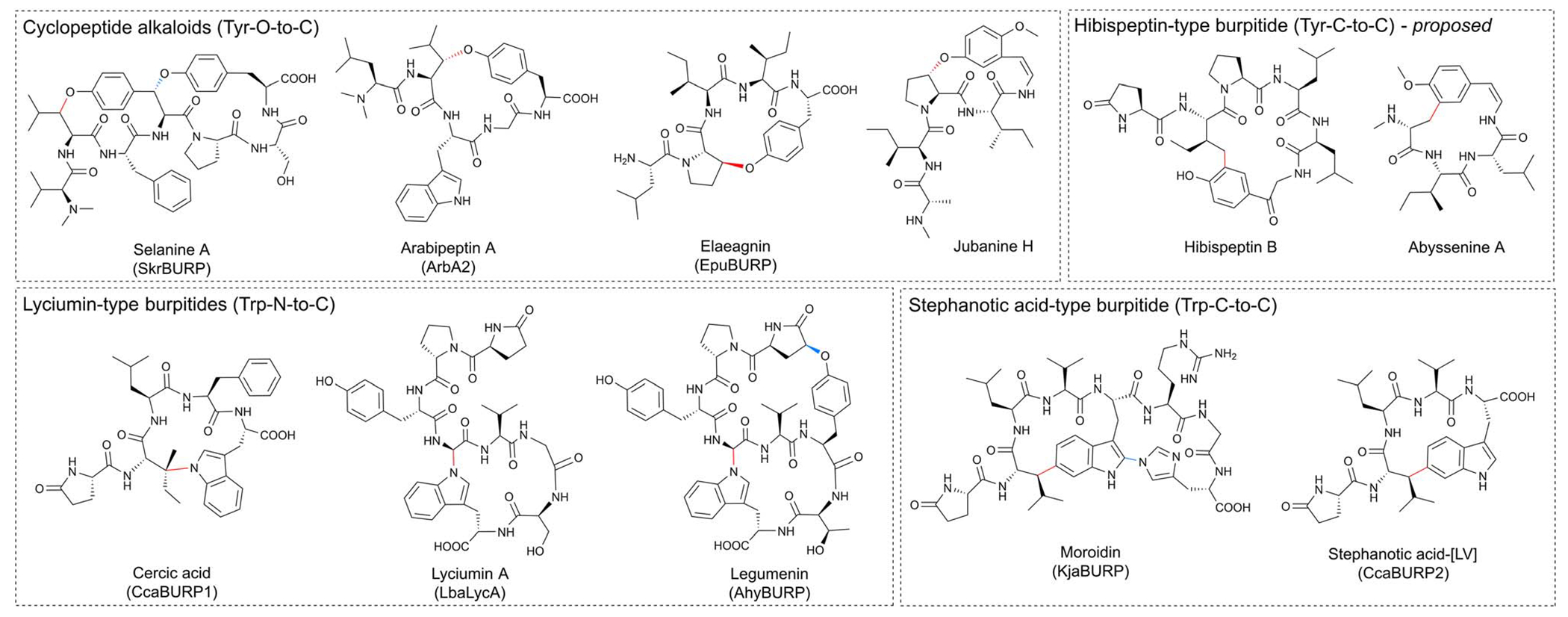
Proposed burpitide classification. Representative precursor peptide names are in brackets. Class-defining bonds are highlighted in red, other burpitide cyclase-derived PTMs are highlighted in blue.

BURP-domain-containing proteins were characterized as precursor peptides for lyciumins and therefore for plant RiPP biosynthesis in 2018.^17^ Subsequently, several additional RiPP classes with new macrocyclic PTMs were revealed as BURP-domain-derived peptides (Fig. 8).^18–20^ Given the rapid expansion of new RiPP classes derived from the BURP domain,^313^ we propose the name of burpitide for this family of peptide natural products biosynthesized using a BURP-domain-containing protein.

The BURP-domain-containing protein family was defined by Hattori and co-workers based on a conserved CHX_10_CHX_25–27_CHX_25–26_CH-motif^313^ in four founding member plant proteins: microspore-derived embryo protein BNM2 from *Brassica napus*,^314^ an unidentified seed protein (USP) from *Vicia faba*,^315^ drought-responsive protein RD22 from *Arabidopsis thaliana*^316^ and the β-subunit of polygalacturonase 1 (PG1β) involved in fruit ripening in *Solanum lycopersicum*.^317^ Bioinformatic analyses of plant genomes show an abundance of BURP-domain genes with diverse primary structures and on average twelve BURP-domain genes per plant genome, ranging from one (*Marchantia polymorpha*) to 53 (*Coffea arabica*).^318^ BURP-domain protein expression is mainly associated with abiotic stress responses in plants.^319,320^ Examples are the expression of Sali3-2 in soybean roots during acidic soil stress.^319^ Some BURP domains are potentially involved in biotic plant stress responses, for instance, a BURP-domain gene is up-regulated in a virus-resistant soybean^321^ and a BURP-domain gene locus mapped to bruchid resistance in mung beans.^322^

In 2022, several BURP-domain-containing precursor peptides were reported as copper-dependent peptide cyclases, which catalyze macrocyclic bond formations in RiPP biosynthesis and define the autocatalytic or fused burpitide pathway.^18^ In 2023, a BURP-domain peptide cyclase with a stand-alone precursor peptide was reported defining the split burpitide pathway.^20^

#### 3.6.1 General recommendations for burpitide nomenclature

##### 3.6.1.1 Macrocyclic bond.

We propose that a macrocyclic bond derived from a BURP-domain-PTM is the primary class-defining feature of a burpitide. Since BURP domains can crosslink aromatic amino acid side chains with many different amino acids *via* diverse chemical bonds, we recommend a burpitide classification based on bond chemistry (C–O, C–N, C–C) and on crosslinked aromatic residues for a given BURP-PTM. For example, cyclopeptide alkaloids would be defined as a burpitide class with C(sp^3^)-O-phenol-ether-crosslinks with the C(sp^3^)-macrocyclization site being any amino acid and the phenol being a tyrosine or similar residue. This classification (Table 2) is inspired by fungal dikaritins RiPPs that are defined by an ether-crosslink between a tyrosine to any amino acid side chain.^2,43,46^

##### 3.6.1.2 Ring size.

Ring size should not be a class-defining feature of a burpitide class. Traditional classification defines cyclopeptide alkaloids as 13-, 14- or 15-membered peptides. However, recent discoveries of 17-membered arabipeptins^20^ and elaeagnins^21^ with cyclopeptide alkaloid-characteristic crosslinks indicate that larger versions of these macrocycles exist in nature. Similarly, ring size is not applied for any RiPP class with a macrocyclic bond as a class-defining feature.^1,2^

##### 3.6.1.3 Ring number and mixed modifications.

Several burpitides have two macrocyclic bonds.^18,19^ Some identified bicyclic burpitides to date have two different types of macrocyclic bonds, which poses the question as to which PTM should be considered class-defining. In this case, we recommend assigning the macrocyclic bond that is formed first during biosynthesis as the class-defining feature. For example, in legumenin biosynthesis the C-terminal lyciumin-bond is formed first,^18^ which defines legumenin as a lyciumin-type burpitide. In moroidin biosynthesis, the N-terminal Leu-Trp-crosslink of the stephanotic acid ring is formed first.^19^ Following the logic of legumenin as lyciumin-type, moroidin is classified as stephanotic-acids type.

##### 3.6.1.4 Terminal modification.

We recommend that terminal modifications should not be class-defining features if a macrocyclic BURP-PTM is present in a burpitide. Common N-terminal modifications of burpitides are *N*-methylation (cyclopeptide alkaloids) and pyroglutamate formation. They are present in multiple burpitides with different macrocyclic bonds (stephanotic acid-type, lyciumin-type, and hibispeptins).

##### 3.6.1.5 Biosynthesis.

We recommend that BURP domains involved in a crosslinking biosynthetic step should be called burpitide cyclases to differentiate them from BURP domains for which no function is known. We also recommend that a split and fused burpitide nomenclature be used to define the biosynthetic route. In the split burpitide pathway, the precursor peptide and burpitide cyclase are encoded for by separate genes,^20^ whereas in the fused burpitide pathway the core peptides are encoded within the same gene as the burpitide cyclase.^18^ To create consistency in split pathways, the burpitide cyclase should be designated with a B (*e.g.* ArbB2) and the precursor peptide should be designated with an A (*e.g.* ArbA2). For fused burpitide cyclases, we recommend naming the enzyme with three letters referring to genus (1 letter) and species (2 letters) and ‘BURP’ (*e.g.* SkrBURP for *Selaginella kraussiana* burpitide cyclase).

##### 3.6.2 Lyciumin-type peptides (Trp-indole-N-to-C).

Lyciumins are originally described as monocyclic octapeptides isolated from *Lycium* plants.^17^ Lyciumin A and B were isolated by a bioactivity-guided approach from root extract of gojiberry (*Lycium barbarum*), which is used in Chinese herbal medicine to treat hypertension.^323^ Since this initial study, an additional 19 lyciumins have been reported including 17 lyciumins from gene-guided discovery approaches.^17,21^ Lyciumins have been detected from Fabaceae, Solanaceae, Berberidaceae, and Amaranthaceae. We recommend the macrocyclic bond definition below of lyciumins that includes cercic acid and legumenin into this burpitide class.

##### 3.6.2.1 Macrocyclic bond.

Lyciumin-type burpitides are defined by a C(sp^3^)-N-macrocyclic bond between a tryptophan-indole-N and the C(sp^3^)-carbon of another amino acid (Fig. 8).

##### 3.6.2.2 Structure.

Lyciumin-type burpitides can be side-chain-to-backbone-macrocyclic or side-chain-to-side-chain-macrocyclic peptides with a class-defining C(sp^3^)-N-macrocyclic bond between the indole-nitrogen of a C-terminal tryptophan to an unactivated carbon of another amino acid (Fig. 8). Known side-chain-to-backbone-macrocyclic lyciumins include founding members lyciumin A and B, which are monocyclic octapeptides and have a C(sp^3^)-N-crosslink between the tryptophan-indole-nitrogen to the Cα of a glycine at the fourth position of the core peptide. The stereochemistry of the macrocyclic bond in lyciumin A has been characterized as *R* by DFT calculations and comparison to experimental data.^324^ Cercic acid is a side-chain-to-side-chain-macrocyclic pentapeptide with a C(sp^3^)-N-crosslink between the tryptophan-indole-nitrogen to the Cβ of an isoleucine at the second position. The stereochemistry of the Ile-Cβ has been determined as *R* based on stereoisomer DFT-calculations and NOE NMR analysis of the purified natural product.^18^ The N-terminus of lyciumins is a pyroglutamate, whereas the C-terminus of most lyciumins is unmodified. Exceptions are lyciumins A and C methylates isolated from *Celosia argentea* with a C-terminal methyl ester group at the C-terminal tryptophan carboxyl group.^325^

A bicyclic lyciumin-type burpitide called legumenin was isolated from alfalfa seeds (*Medicago sativa*) based on gene-guided discovery approach. Legumenin is an octapeptide derived from the core peptide sequence QPYGVYTW that has a lyciumin-bond between a C-terminal tryptophan and a glycine at the fourth position and an ether bond between the sixth position Tyr-phenol-OH and the first position PyroGlu-Cγ. The stereochemistry of the PyroGlu-Cγ was determined as *S* based on stereoisomer DFT-calculations and comparison to NOE NMR analytical data of the isolated peptide.^18^

##### 3.6.2.3 Biosynthesis.

Lyciumins have been characterized as RiPPs by the discovery of precursor peptide gene *LbaLycA* from a root transcriptome of *Lycium barbarum*.^17^ LbaLycA has twelve core peptides encoding for three lyciumins (A, B, D) in a highly repetitive N-terminal domain and a C-terminal BURP domain. The heterologous expression of *LbaLycA* in *Nicotiana benthamiana via Agrobacterium tumefaciens* infiltration and the pEAQ-HT expression system resulted in the formation of the lyciumins corresponding to LbaLycA-core peptides in transgenic tobacco leaves after six days. In addition, a BURP-domain-containing protein with an internal lyciumin core peptide called Sali3-2 was identified by bioinformatic searches for BURP-domain lyciumin precursor peptides. Sali3-2 is a homolog of the founding member USP from soybean, comprised of the only one lyciumin I core peptide QPYGVYTW, and has been implicated in increased abiotic tolerance in plants grown in metal-contaminated soil.^326^ Lyciumin I was detected in the root and seed pods of soybean plants and in the leaf extracts of transgenic tobacco expressing *Sali3-2*.^17^ In addition, bicyclic legumenin was detected in transient gene expression experiments of Sali3-2 homolog AhyBURP with the same core peptide.^18^ Transient tobacco expression confirmed a lyciumin precursor peptide from the potato, StuBURP, which is connected to production of lyciumin J (core: QPYGVFAW).^17^ Lastly, transient expression of the cercic acid precursor CcaBURP1 in tobacco resulted in the biosynthesis of cercic acid.

**Fig. 9:**
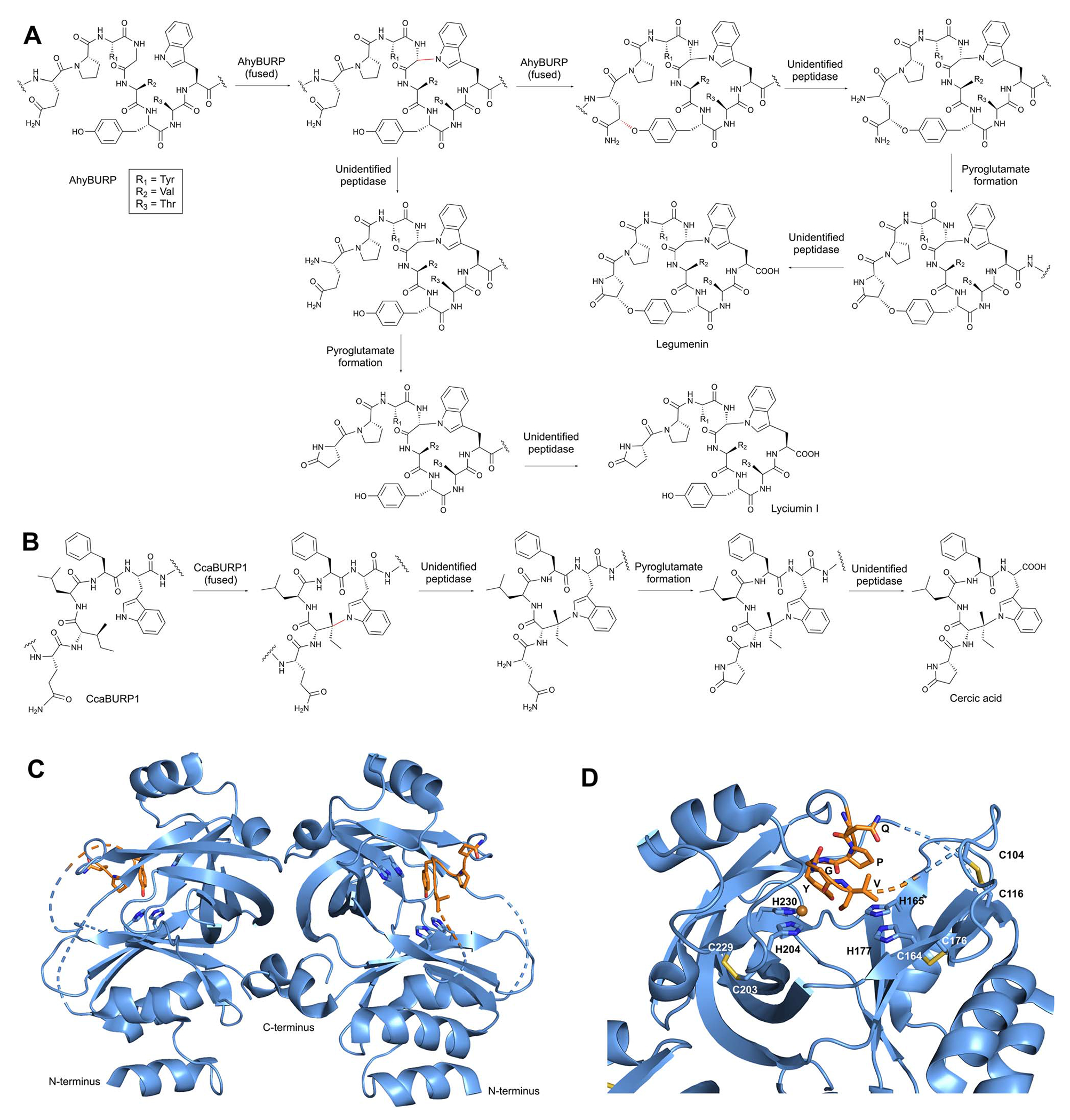
Biosynthetic proposal of lyciumin-type peptides. (A) Proposed formation of lyciumin I and legumenin from AhyBURP (B) Proposed formation of cercic acid from CcaBURP1. (C) The homodimer of AhyBURP, PDB ID 8SY2. Each subunit is in blue, and the core peptides are in orange and colored according to element (oxygen, red; nitrogen, blue). The core peptide and conserved His of BURP-domain-containing proteins are shown as sticks. Main chain atoms are omitted for clarity. (D) Copper-bound structure of AhyBURP, PDB ID 8SY3. The core peptide and conserved Cys and His residues of BURP-domain proteins are shown as sticks (sulfur, yellow). Main chain atoms for Cys-His are omitted for clarity. Dashed lines in (C) and (D) represent disordered regions in the crystal structure.

A Sali3-2 homolog from peanut called AhyBURP with the same legumenin core peptide was further characterized as a copper-dependent peptide cyclase by bottom-up proteomic analysis. Herein, a mass loss matching a legumenin macrocyclization in the core peptide region was detected. A lyciumin-type bond formation was detected in the modified bicyclic core peptide after exopeptidase incubation of the purified core peptide and comparison to legumenin. AhyBURP *in vitro* reconstitution further established that the lyciumin bond is formed before the PyroGlu1-Tyr6-crosslink is formed (Fig. 9A).^18^ A direct characterization of a lyciumin PTM in a modified core peptide was done in the cercic acid pathway from Eastern redbud (*Cercis canadensis*). A truncated single core peptide construct of cercic acid BURP-domain precursor CcaBURP1 was reconstituted *in vitro* with copper, treated with exopeptidases, and the resulting modified QILFW core peptide yielded an analyte matching a cercic acid standard^18^ (Fig. 9B).

The AhyBURP protein structure was recently determined by X-ray crystallography.^327^ AhyBURP is a homodimer in solution and in the crystal structure (Fig. 9C). The BURP-domain fold represented in the AhyBURP subunit is unique among experimentally determined protein folds. In each AhyBURP subunit, a cleft is found, composed of a β-barrel and a β-sheet that face each other, overlaid by the core peptide substrate. The four conserved Cys–His residues of BURP-domain proteins are in this cleft, which forms the active site of two Type II copper centers.^328^ Two conserved Cys residues form a disulfide staple and the sequential, conserved His residues extend into the cleft from the β-sheet, and the same arrangement is found on the facing β-barrel (Fig. 9D). The active sites of the dimer are separated by about 50 Å, and each core peptide is positioned to react in *cis* to its active site subunit. This is contrary to fungal autocatalytic peptide-*N*-methyltransferases, where the substrate core peptides react in *trans* across a dimer interface.^329^ In addition to the structural characterization of AhyBURP, functional studies were reported.^327^ AhyBURP can use Cu(i) or Cu(ii) to facilitate peptide macrocyclization, it requires dioxygen for activity and isotopic labeling with deuterated Gln in the core peptide demonstrated a loss of 1 D and 3 H in the presence of Cu(ii), suggesting a radical-based reaction. Isotopic labeling studies further supported an intramolecular reaction within the AhyBURP subunit and its core peptide. Lastly, tandem mass spectrometry identified two covalent radical trap adducts within the core peptide region, indicating that two radicals can be detected within the core peptide in the presence of Cu(ii). Many questions emerge from this work, including which steps in the mechanism use single electron transfer, how and when dioxygen is bound, and the copper coordination state throughout the reaction.

The N-terminal pyroglutamate is generated from glutamine, which can spontaneously cyclize to pyroglutamate after proteolytic cleavage at the N-terminus of a lyciumin core peptide. The involvement of glutamine cyclotransferase has been proposed based on co-localization of glutamine cyclotransferase genes with fused lyciumin precursor peptides in the beet genome.^17^ To date, all characterized lyciumins are derived through the fused burpitide pathway.

##### 3.6.2.4 Bioactivity.

Lyciumin A and B showed moderate inhibition of renin and angiotensin-converting enzyme.^323^ No endogenous functions of lyciumins have been described yet. They are detected in diverse plant tissues and developmental stages, including in storage tissues such as seeds (amaranth, soybean), roots (gojiberry), and sprouts (potato). The functions of legumenin and cercic acid are also unknown.

#### 3.6.3 Cyclopeptide alkaloids (Tyr-phenol-O-to-C).

Cyclopeptide alkaloids (CPA)^330^ are four to six amino acids in length and represent a large plant peptide class with >230 known compounds.^3,18,331^ The high number of cyclopeptide alkaloids is based on their diversity of macrocyclic bonds, and N- and C-terminal functionalities. Traditionally, cyclopeptide alkaloids have been defined by macrocyclization *via* a phenol group of a C-terminal hydroxystyrylamine, octopamine, or tyrosine residue to an unactivated carbon at an amino acid side chain two positions N-terminally from the phenol-bearing residue. In the traditional classification system, the phenol-crosslinks are usually ethers involving the phenolic hydroxy group with rare exceptions having C(sp^2^)-C(sp^3^)-crosslinks between the meta-carbon of a phenol to a different amino acid side chain. Furthermore, the previous classification limited cyclopeptide alkaloids to 13-, 14- and 15-membered macrocycles.^3,331,332^ As burpitides emerge within the biosynthetic description of cyclopeptide alkaloids,^18,20^ and their structural similarity to the fungal RiPP class dikaritins,^2,43–46^ we propose a revised classification of cyclopeptide alkaloids.

##### 3.6.3.1 Macrocyclic bond.

Cyclopeptide alkaloid-type burpitides are defined by phenol-ether-crosslinks formed by burpitide cyclases (Fig. 1B and 8).^333,334^ The phenol group-crosslinks in cyclopeptide alkaloid-type burpitides are derived from tyrosine residues, however the biosynthesis of cyclopeptide alkaloids containing hydroxystyrylamine and octopamine moieties is largely uncharacterized.^331^ Several cyclic plant peptides previously defined as cyclopeptide alkaloids contain C(sp^3^)-C(sp^2^)-crosslinks involving a phenolic carbon at the meta-position, for example, abyssenine A^335^ (Fig. 8). Due to the different chemistry of their macrocyclic bond, we suggest excluding these non-ether-crosslinked peptides from the nomenclature of cyclopeptide alkaloids and define them as a separate peptide class (Section 3.6.5).

##### 3.6.3.2 Ring size.

Cyclopeptide alkaloid rings range from 13–17 atoms, but other sizes may exist.

##### 3.6.3.3 Ring number.

Cyclopeptide alkaloids can have more than one macrocycle. In peptides with different macrocyclic bonds, the first macrocyclic bond formed defines the peptide class. For example, legumenin is not considered as a cyclopeptide alkaloid but a lyciumin-type peptide due to the first formed lyciumin-bond despite having a Tyr-O-PyroGlu-Cγ-crosslink (see Section 3.6.2). Since many cyclopeptide alkaloids await biosynthetic definition, this nomenclature is subject to refinement.

##### 3.6.3.4 Structure.

**Fig. 10:**
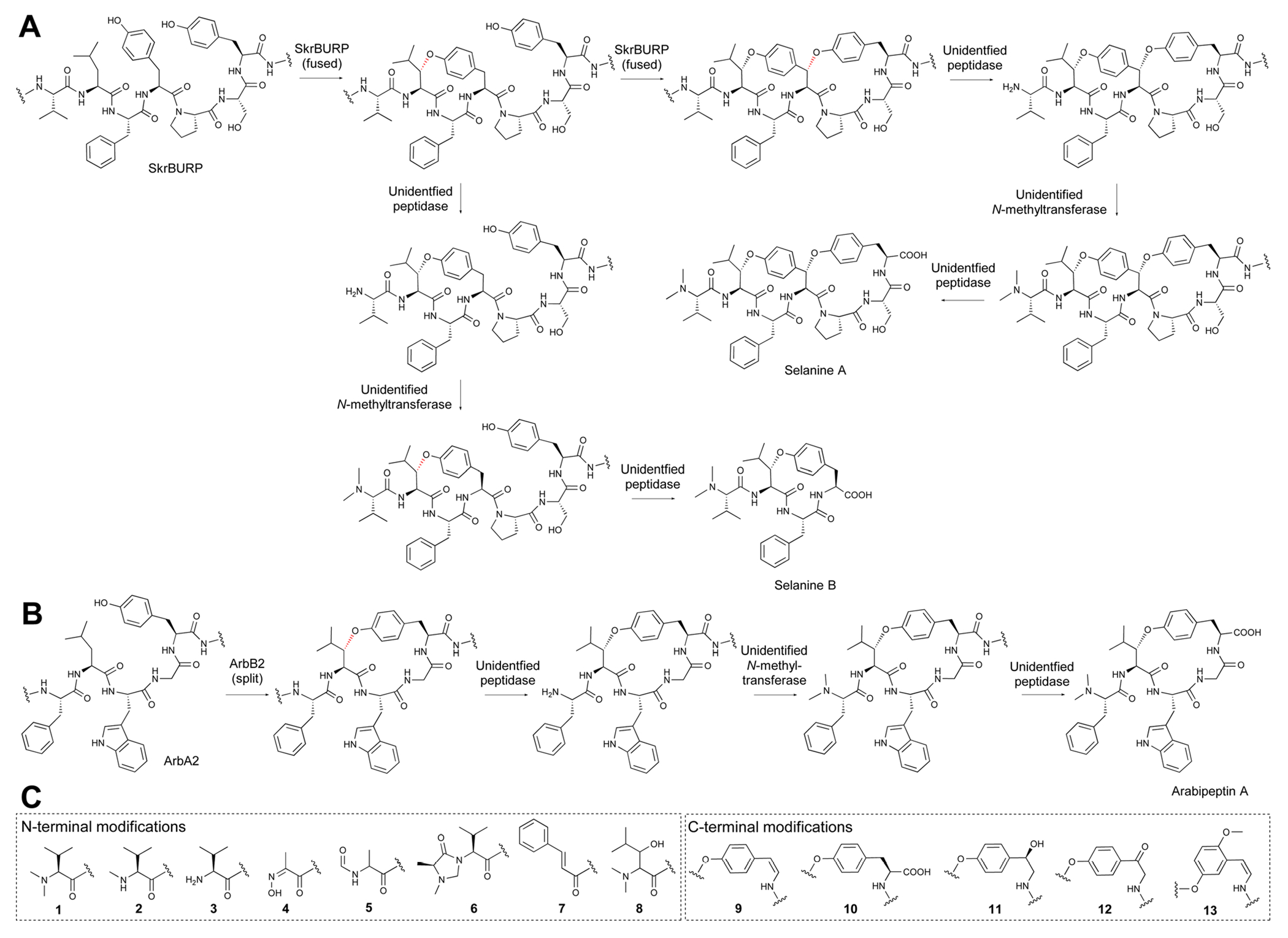
Biosynthetic proposal for cyclopeptide alkaloids. (A) Proposed formation of mono- and bicyclic peptide alkaloids from SkrBURP in a fused burpitide pathway. (B) The proposed split burpitide pathway for the production of arabipeptin A. (C) N-terminal modifications observed in cyclopeptide alkaloids: (1) *N*,*N*-dimethylation, (2) *N*-monomethylation, (3) unmodified, (4) *N*-oxime, (5) *N*-formylation, (6) cyclized *N*-methylation (imidizolidine-4-one), (7) deamination (cinnamic acid), (8) Cβ-hydroxylation, C-terminal modifications observed in cyclopeptide alkaloids: (9) *p*-hydroxy-styrylamine, (10) tyrosine, (11) octopamine, (12) 4-hydroxy-α-aminoacetophenone (13) *m*-hydroxy-*o*-methoxy-styrylamine.^3^

As recommended above, cyclopeptide alkaloids are defined as side-chain-to-side-chain-macrocyclic peptides with a phenol-derived ether bond. The ether bond can involve a C-terminal *meta*- or *para*-phenol hydroxy-group such as in jubanine H or sanjoinine A, respectively (Fig. 10). The C-terminal phenol-containing residue can be a tyrosine in vignatic acid A, a *p*-hydroxystyrylamine in sanjoinine A, a *m*-hydroxystyrylamine in jubanine H (Fig. 8), or an octopamine in pandamine.^336^ The residues *p*-hydroxystyrylamine or *m*-hydroxystyrylamine can have additional *o*-hydroxy or *o*-methoxy groups, as observed in ramosine C^337^ or in jubanine H,^333^ respectively. The cyclopeptide alkaloid encephanine has an *O*-glycosylation at the *m*-position of a *p*-hydroxylstyrylamine and represents the only glycosylated plant cyclopeptide to date.^18^

The double bond configuration of cyclopeptide alkaloid styrylamine is usually *Z*. The macrocyclization site in the other residue of the ether bridge is usually the β-carbon of a leucine, proline, phenylalanine, tyrosine, isoleucine, or valine.^3^ 94.1% (64/68) of reported cyclopeptide alkaloids structures have an *S*-configuration at this cyclization site.^3^ The N-terminus of cyclopeptide alkaloids is usually *N*-mono- or *N*,*N*-dimethylated. Additional N-terminal functional groups include cinnamic acid, deaminated leucine,^338^ 2-(hydroxyimino)propanoic acid, *N*-oximes, *N*-formyl groups and cyclized *N*-methylations such as imidizolidine-4-one (Fig. 10C).^3,339^ Almost all known cyclopeptide alkaloids are monocyclic, however bicyclic cyclopeptide alkaloids have been determined. Selanine A from African clubmoss has a di-ether-bridge *via* a Leu-Tyr-Tyr-crosslink.^18^ Selanine A is similar in overall macrocycle structure to fungal dikaritin RiPP asperipin-2a except for an *S*-configuration at both Cβ-cyclization sites in selanine A.^340^

##### 3.6.3.5 Biosynthesis.

Recently, a 14-membered cyclopeptide alkaloid with a Tyr-O-Leu-Cβ-crosslink called selanine A was characterized as a burpitide derived from a fused burpitide pathway in African clubmoss (*Selaginella kraussiana*)^18^ and a 17-membered cyclopeptide alkaloid with a Tyr-O-Leu-Cβ-crosslink named arabipeptin A was discovered as a burpitide derived from a split burpitide pathway in *Coffea arabica*.^20^ Furthermore, 17-membered burpitides with Tyr-O-Pro-crosslinks have been proposed and identified as fused burpitide pathway products in soybean and silverberry (*Elaeagnus pungens*), respectively.^18,21^

The fused cyclopeptide alkaloid pathway from African clubmoss starts with BURP-domain precursor peptide SkrBURP, which has four core peptide sequence repeats of VLFYPSY in the N-terminal domain. Transient expression of native SkrBURP and a truncated SkrBURP with only one core peptide (SkrBURP-1xVLFYPSY) in tobacco led to the production of desmethyl-selanine A and desmethyl-selanine B, which matched the purified selanine A and B after reductive *N,N*-dimethylation. This experiment showed that the clubmoss cyclopeptide alkaloids are burpitides. Furthermore, the formation of the cyclopeptide alkaloid bonds between the Tyr-O-Leu-Cβ in selanine A and between the Tyr-O-Tyr-Cβ was reconstituted *in vitro* from purified SkrBURP-1xVLFYPSY in the presence of copper and characterized by bottom-up proteomics of the core peptide, exopeptidase reaction, and comparison to desmethyl-selanine A and B analytes from SkrBURP tobacco expression experiment.^18^ This result showed that cyclopeptide alkaloid ether-bonds can be formed by fused burpitide cyclases. In an automated genome mining study, mono- and bicyclic cyclopeptide alkaloids analytes matching core peptides ILLYPSY and FLLYPY in SkrBURP could be detected in extracts of *S. kraussiana* and as corresponding analogs in transient tobacco extracts of SkrBURP expression.^21^ These observations indicated that burpitide cyclases such as SkrBURP enable the formation of both bi- and monocyclic cyclopeptide alkaloid diversification from a single fused BURP-domain gene (Fig. 10A).

Some of the most prolific producers of cyclopeptide alkaloids are in the buckthorn family (Rhamnaceae) with prominent source plants being New Jersey Tea (*Ceanothus americanus*) and jujube (*Ziziphus jujuba*). A recent study^20^ identified repetitive proteins encoded in transcripts of *C. americanus* that had short sequence motifs matching known cyclopeptide alkaloid structures from *C. americanus*. Homologs of these putative cyclopeptide alkaloids were further identified in the genome of *Z. jujuba* and matched jujube cyclopeptide alkaloid structures. Notably, they were co-localized with stand-alone BURP-domain genes in the jujube genome. This strongly suggested the presence of a split burpitide pathway wherein the precursor peptide and burpitide cyclase are translated as two separate polypeptides instead of a fused one. Multiple copies of similar split cyclopeptide alkaloid precursor peptides were found in the *Coffea arabica* genome. One of these hypothetical precursor peptides contained three copies of the core peptide FLWGY flanked by recognition sequences and was located adjacent to a putative burpitide cyclase gene. This observation guided the isolation of a new cyclopeptide alkaloid arabipeptin A. *In vitro* reconstitution of the corresponding burpitide cyclase ArbB2 with the split precursor peptide ArbA2 resulted in the detection of a mass shift matching the Tyr-O-Leu-Cβ macrocyclization observed in arabipeptin A and thus showed that cyclopeptide alkaloids can be biosynthesized through a split burpitide pathway (Fig. 10B).

Based on the reconstitution of multiple pathways, precursor peptides for cyclopeptide alkaloids typically contain multiple core sequences and are cyclized by both split and fused burpitide cyclases. However, the downstream biosynthetic enzymes involved in the N- and C-terminal modification of cyclopeptide alkaloids are mainly unknown. A cyclopeptide alkaloid-guided genome-wide-association-study in *Z. jujuba* identified a putative *N*-methyltransferase for the N-terminal dimethylation of sanjoinine A co-localized with a BURP-domain gene and belongs to an unknown plant SAM-dependent methyltransferase clade.^341^ Methylation activity in a cyclopeptide alkaloid analyte was proposed based on transient gene expression of the methyltransferase in white mature jujube fruits and detection of sanjoinine A formation compared to an empty vector control. The enzymatic basis for the formation of the C-terminal hydroxystyrylamine observed in many cyclopeptide alkaloids remains to be elucidated.

##### 3.6.3.6. Bioactivity.

Several cyclopeptide alkaloids have been studied for their anxiolytic and analgesic effects. Sanjoinine A is a bioactive ingredient of jujube used in herbal medicine to treat insomnia and further displays GABA-adrenergic activity to reduce insomnia.^342^ Studies in mouse models demonstrated that sanjoinine A also has anti-anxiety activity, likely mediated again through the GABA_A_ receptor.^343^ Studies with adouetine X^344^ and multiple cyclopeptide alkaloids from *Ziziphus oxyphylla*^345^ showed analgesic activity in multiple mouse pain models. Subsequent *in vitro* assays with adouetine X suggest that analgesic activity may be due to inhibition of Ca^2+^-ATPase and Na^+^/K^+^ ATPase. A similar inhibition was observed with sanjoinine A and sanjoinine F.^346^

Another jujube cyclopeptide alkaloid, jubanine H, was recently reported to have antiviral activity against a pig coronavirus and low mammalian cell cytotoxicity by *in vitro* viral infection assays.^333^ Numerous cyclopeptide alkaloids have been investigated for antiplasmodial activity and demonstrated single digit μM IC_50_ values, several such as nummularine R, spinanine B, and adouetine X.^347,348^ The cyclopeptide alkaloid vignatic acid A from mung bean has insecticidal activity against the azuki bean weevil (*Callosobruchus chinensis*)^349^ and it has been hypothesized to act as a resistance-conferring product of a mung bean beetle resistance gene. One of the hypothesized resistance genes is a SkrBURP homolog called resistant-specific protein 1(4) with core peptides matching vignatic acid A.^349^ It was shown that the presence of resistant-specific protein 1(4) does not fully confer resistance to *C. chinensis* suggesting that other genes might be involved in the resistance mechanism.^350^

#### 3.6.4 Stephanotic acid-type peptides (Trp-C-to-C).

Moroidin is a bicyclic peptide which was originally isolated from Australian stinging nettle *Dendrocnide moroides* and recently was characterized as a burpitide.^19^ The N-terminal macrocycle of moroidin is also found in the burpitide stephanotic acid, which was first isolated from *Stephanotis floribunda*^351^ and then biosynthetically characterized as a burpitide from *Cercis canadensis*.^18^ Given a structural overlap of moroidin and stephanotic acid, we include them as stephanotic acid-type burpitides and recommend the following nomenclature below for this RiPP class.

##### 3.6.4.1 Macrocyclic bond.

Stephanotic acid-type peptides are defined by a C(sp^3^)-C(sp^2^)-crosslinks between a carbon of a tryptophan-indole and a carbon of another amino acid.

##### 3.6.4.2 Structure.

Moroidins and stephanotic acids are side-chain-to-side-chain-macrocyclic peptides. Moroidin has a bicyclic core structure with a class-defining crosslink between the Leu2-Cβ and the Trp5-indole-C6 (stephanotic acid crosslink) and an additional crosslink between the Trp5-indole-C2 and a His8-imidazole-N1. The Leu-Cβ has *S*-stereochemistry as determined by X-ray crystallography.^352,353^ The Trp-His-bond is the only example to date of two crosslinked aromatic rings in a burpitide. Moroidin and ten moroidin analogs have been reported from *Celosia argentea*, named celogentin A–K.^353–355^ Several of the celogentins have extended linear C-termini. Another moroidin analog called moroidin-[QLLVWRSH] was also discovered from *Bauhinia tomentosa*.^19^ Three stephanotic acids have been characterized so far with core peptides QLIVW, QLLVW and QLKVW. Both moroidins and stephanotic acids share a l-pyroglutamate at the N-terminus.

##### 3.6.4.3 Biosynthesis.

**Fig. 11:**
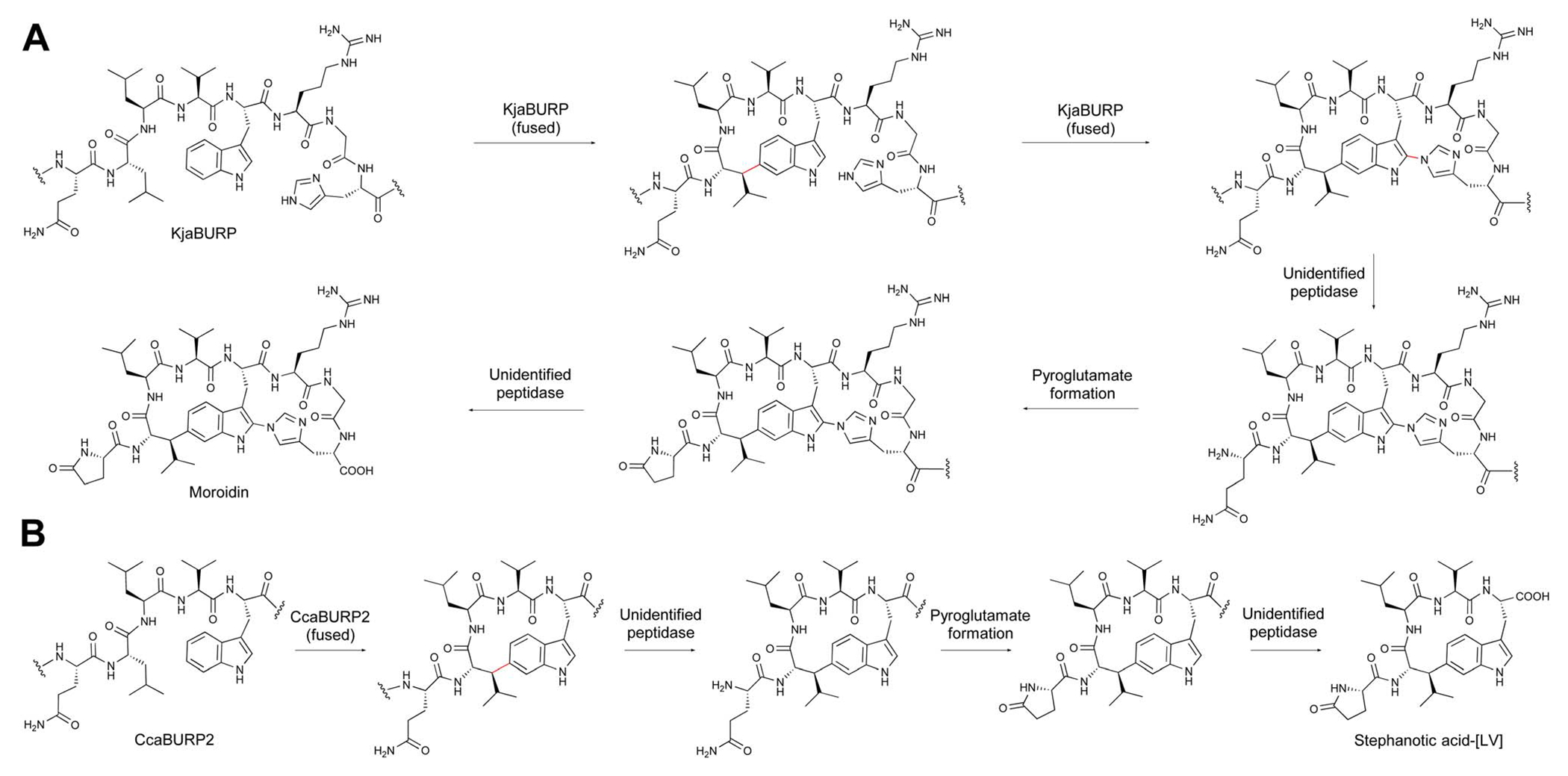
Biosynthetic proposal for stephanotic acid-type burpitides. (A) Proposed biosynthesis of moroidin in *Kerria japonica*. (B) Proposed biosynthesis of stephanotic acid-[LV] in *Cercis canadensis*.

Moroidin was characterized as a burpitide derived from a fused burpitide pathway from *Kerria japonica* by *in planta* and *in vitro* reconstitution of the moroidin precursor peptide KjaBURP (Fig. 11A).^19^ KjaBURP has four repeats in the N-terminal domain, including three moroidin core peptides (QLLVWRGH) and one moroidin-[QLLVWRAH] core peptide. Transient expression of *KjaBURP* in tobacco yielded moroidin and moroidin-[QLLVWRAH] analytes and *in vitro* reconstitution of KjaBURP-[1xQLLVWRGH] with copper and subsequent endo- and exoproteolytic digests also resulted in the formation of a moroidin analyte. *In planta* reconstitution showed that the Leu-Trp-crosslink forms first and the Trp-His-crosslink second. *In planta* and *in vitro* analysis resulted in the detection of stephanotic acid-[LV] and no detection of an analyte corresponding to the second ring. The pyroglutamate in moroidin can form spontaneously from glutamine after N-terminal cleavage of the Gln1. A similar biosynthetic pathway has been characterized for stephanotic acid-[LV] from *Cercis canadensis* burpitide cyclase CcaBURP1 (Fig. 11B).^18^

##### 3.6.4.4 Bioactivity.

Moroidin was discovered during a study for pain-causing agents from *D. moroides*. While pain-causing activity was reported for purified moroidin,^356^ no pharmacological studies exist about the pain-causing activity of moroidins. Moroidin and celogentin C have *in vitro* anti-tubulin polymerization activity and *in vitro* lung adenocarcinoma cell cytotoxicity with reported low micromolar IC_50_.^19,352^ Stephanotic acid-[LV] has no significant *in vitro* cytotoxicity against a tested lung cancer cell line indicating that the second Trp-His ring is important for cancer cell cytotoxicity.^18^ Endogenous functions of moroidins and stephanotic acids in source plants are not established.

#### 3.6.5 Hibispeptin-type peptides (Tyr-C-to-C).

The type defining molecules, hibispeptins A and B, are produced by the well-known shrub *Hibiscus syriacus* (Fig. 8).^357,358^ These molecules are cyclized through a carbon–carbon crosslink between the C-terminal 2-amino-3-(2-hydroxy-5-aminoacetylbenzyl) pentanoic acid (Ahabpa) and the γ-methyl of l-isoleucine. To date, no enzymatic or heterologous reconstitution has been completed for hibispeptins to prove that they are burpitides. However, precursor peptides that mimic the split precursor peptides of cyclopeptide alkaloids and include cores matching known hibispeptins along with the genomic co-localization of a BURP-domain-containing gene strongly suggests their biosynthetic classification as burpitides.^20^

##### 3.6.5.1 Macrocyclic bond.

Hibispeptin-type burpitides are characterized by a C(sp^3^)-C(sp^2^)-macrocyclic bond between a C-terminal phenolic amino acid, such as tyrosine or a tyrosine-derived residue, and the C(sp^3^)-carbon of another amino acid. This definition also encompasses a few molecules that were originally classified as cyclopeptide alkaloids, such as abyssenine A (Fig. 8).^335^

##### 3.6.5.2 Structure.

The original hibispeptin A and B peptides are six amino acids in length and the hypothesized core sequences are largely conserved by amino acid sequence as QIPLFY and QIPLLY, respectively.^357,358^ In each case, the N-terminal glutamine has been cyclized to pyroglutamate. In both hibispeptin A and B the cyclization occurs between the γ-methyl of l-isoleucine and the *meta* position of the tyrosine derived Ahabpa residue. As stated, we propose to expand the hibispeptin-type burpitides to include all burpitides with a C(sp^3^)-C(sp^2^)-macrocyclic bond from a C-terminal phenyl ring-containing amino acid. Natural products such as abyssenine A–C (Fig. 8) and mucronine A–H fall into this classification.^335^ They are each five amino acids in length with a terminal *p-O*-methoxy-styrylamine residue crosslinked between the *meta*-position to the beta-position of the first amino acid. The N-terminus is often methylated and an additional *o*-methoxy modification is often observed on the *p-O*-methoxy-styrylamine as in mucronine E.^3^

##### 3.6.5.3 Biosynthesis.

Recent work on *H. syriacus* has identified candidate split precursor peptides for both hibispeptin A and B.^20^ These potential precursor peptide genes for hibispeptin A and B were located adjacent to putative split burpitide cyclases, strongly implying that they are biosynthesized as part of a split burpitide pathway. The adjacent genes for hibispeptin A and B resemble the split burpitide biosynthetic pathway observed in arabipeptin A. Additional precursor peptides were identified containing core peptides that did not match either hibispeptin A or B, such as QVPLVY. Metabolomic analysis of *H. syriacus* root extract indicated a presence of a mass spectrometric feature and MS/MS fragmentation data supporting this new hibispeptin analogue.^20^

##### 3.6.5.4 Bioactivity.

To date, hibispeptin-type burpitides have minimal noted bioactivity. Hibispeptin A demonstrated weak inhibition of lipid peroxidation^358^ and abyssenine A and mucronine E showed weak cytotoxic activity.^359^ Antimicrobial tests demonstrated that mucronins E, G, and H and abyssenines A and C have weak antibacterial activity, whereas abyssenine A has some antifungal activity.^335^

## 4. Undefined plant-derived peptides

While the molecules discussed in Section 3 have been established as plant produced RiPP natural products, numerous other peptides have been isolated from plants for which little biosynthetic information is available. Moreover, the authentic producer may also be unclear. In many cases, plants harbor endophytic bacteria or fungi that are associated with a particular species or genus. These microorganisms have been shown to be responsible for the production of many peptidic natural products, further complicating biosynthetic elucidation of a target molecule.^31,360^ For example, the astin class of peptides was shown to be biosynthesized by an endophytic fungus.^361^ Below we discuss one prevalent class of biosynthetically undefined bioactive plant peptides, the bouvardins.

### 4.1. Bouvardins

**Fig. 12:**
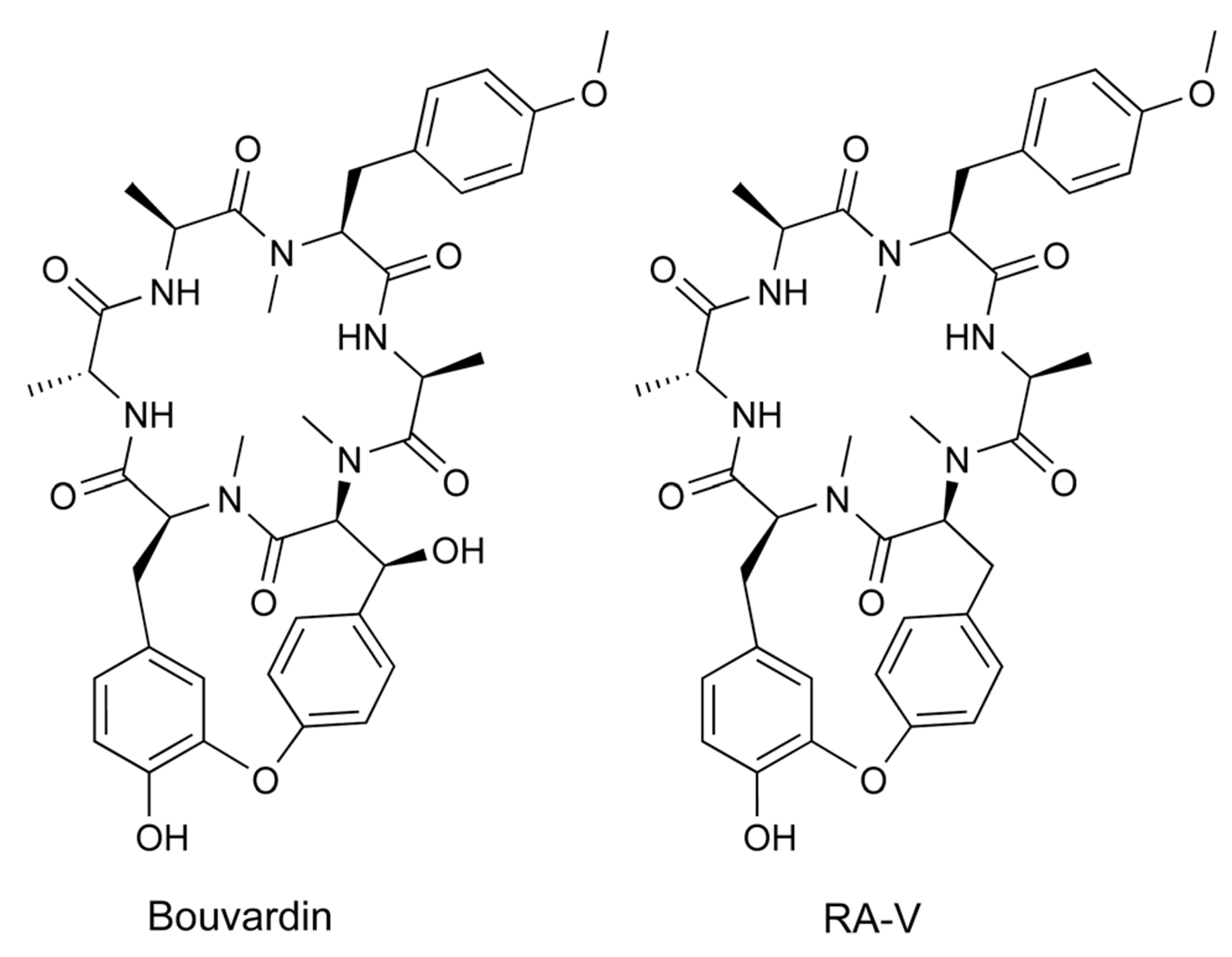
Bouvardin structures.

Bouvardins are a particularly noteworthy class of cyclic peptides isolated from plants for which both the true producer and biosynthetic route is unknown. Bouvardin (Fig. 12) was first isolated in 1977 from *Bouvardia ternifolia*.^362^ Since then, over 30 analogues have been found in members of the *Rubia* genus including the RA-series,^363,364^ the rubiyunnanins,^365,366^ and the rubicordins.^367^

#### 4.1.1 Structure.

Bouvardins are bicyclic peptides that are composed of both an N- to C-amide macrocycle and an ether bond formed from the phenolic oxygen of a tyrosine and the *meta* carbon of an adjacent tyrosine. They are six amino acids in length and are largely comprised of hydrophobic amino acids. Known bouvardins also contain three separate *N*-methylations of the amide. A d-Ala is found at the same position in all known bouvardins, and the β-carbon of the ether forming tyrosine is often hydroxylated. The free phenolic oxygens are often methylated or glycosylated.^3^

#### 4.1.2 Biosynthesis.

To date, details on the biosynthesis of bouvardins are scarce. Isolation of endophytes from RA-producing plants of the *Rubia* genus identified four stains that produced metabolites consistent with the RA-series of bouvardins by LC-MS/MS.^368^ Whether these molecules are produced by plants or an endophyte, the biosynthetic route remains to be elucidated.

#### 4.1.3 Bioactivity.

The bouvardin class of cyclic peptides has been investigated for antitumor properties. Bouvardin was shown to be active against leukemia and melanoma cells lines.^362^ Subsequent mechanistic studies found that bouvardin inhibits protein synthesis by binding to the 80S subunit of the ribosome,^369^ likely stabilizing the interaction between the 80S subunit and elongation factor 2.^370^ The RA-series of analogues are also cytotoxic. RA-IV, RA-V, and RA-VII were the first members of the RA-series isolated and each showed promising antitumor activity in mouse studies including leukemia, melanomas, carcinomas, and solid tumors.^371^ Later studies found that RA-V^372^ and RA-XII^373^ are also effective against breast cancer cell lines and appears to limit adhesion, motility, and invasion of the cancer cells, possibly through the PI3K/AKT signaling pathway. In the case of RA-V, the cytotoxic activity was linked to the activation of apoptosis through a mitochondrial pathway.^372^ In addition, the rubiyunnanins and the rubicordins^367^ have some cytotoxic bioactivity as well and members of the bouvardin class of cyclic peptides also demonstrate anti-angiogenesis,^373,374^ NO synthesis inhibition,^366^ anti-inflammatory,^375^ and NF-κB pathway modulation activities.^366,367,375^

## 5. Chemotaxonomy of plant peptides

We sought to understand the chemotaxonomic distribution of plant RiPPs to identify trends and hot spots where specific peptides may be found. To assemble this data, we identified the order responsible for producing every known molecule in the cyclotide, PawS-derived peptide, orbitide, cysteine rich peptide, and burpitide classes of RiPPs. The presence of these peptides was limited to vascularized plants (Tracheophyta). To visualize the distribution of the plant RiPPs in tracheophytes, we collected the orders within Tracheophyta from the NCBI taxonomy database and used a combination of phyloT and the ggtree R package to generate a cladogram.^376^ The cladogram was then annotated with the RiPP classes data. Our cladogram does not include RALF peptides.

**Fig. 13:**
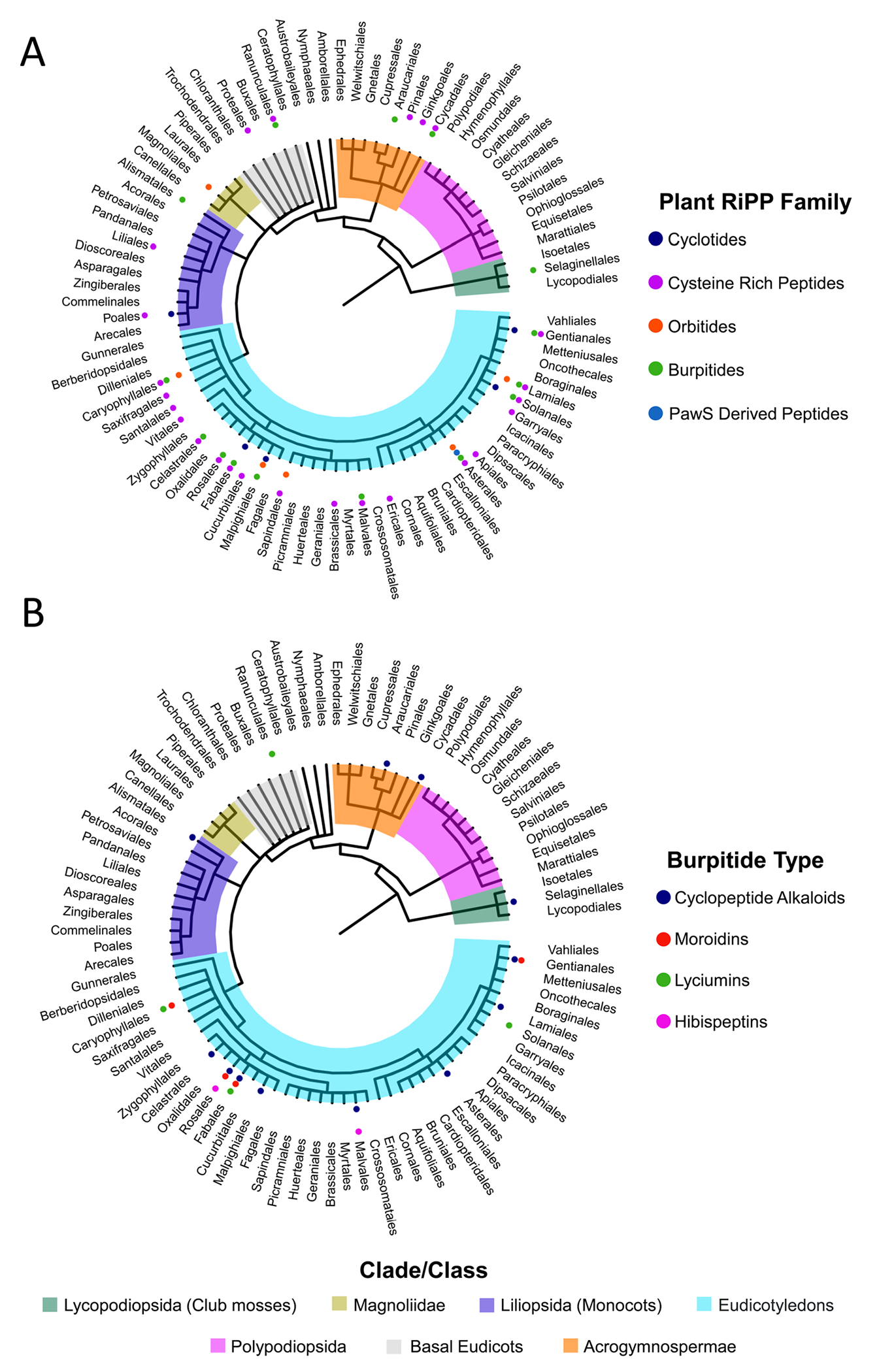
Chemtaxonomy of Plant RiPPs. Cladograms annotated with the detected presence of plant RiPPs (A) and specific burpitide types (B).

Fig. 13A shows the presence of the different RiPP classes found in plants. The results indicate that eudicots are the most prolific producers of RiPP natural products in plants, with representatives of all five types present. In particular, the rosid clade, including members such as Fabales and Malpighiales, appears to be a particular biosynthetic hot spot. Also of note is the Asterales order which has been shown to biosynthesize all five classes of plant RiPPs. Outside of eudicots, monocots also contain members that produce RiPPs, but to a lesser extent. The true ferns (Polyopsida) have had no RiPPs from these five classes identified. The cladogram also revealed a differential distribution of each plant RiPP. For example, PawS-derived peptides (PDPs) have only been found in the order Asterales. In contrast, cysteine-rich peptides and burpitides are more widely distributed across nearly all Tracheophyta clades.

We also generated a second cladogram focused on the distribution of the burpitides (Fig. 13B). Burpitide diversity seems to follow the same trend as all plant RiPP classes because the rosids have the largest diversity of isolated burpitides. Specifically, the Fabales and Rosales both contain three out of the four types of molecules contained within the burpitide class. This cladogram also highlights the fact that cyclopeptide alkaloids are the most widely distributed burpitide discovered to date.

It is important to note that while this figure may not show the authentic distribution of plant RiPPs in nature. As with all isolation experiments, the results can be biased by what specific molecules are being sought, what plant materials are available, and the isolation methodology. The increasing prevalence of plant genomes and transcriptomes is offering a way to target isolation in unexpected producers, such as selanine A in the African clubmoss of the Selaginellales order (see Section 3.6.3).

## 6. Discovery of plant RiPPs

Plant RiPPs have been discovered through bioactivity-guided, structure-guided, and gene-guided approaches that will be reviewed based on representative examples rather than a comprehensive list of discoveries below. A bioactivity-guided approach is less selective for compound classes, whereas structure-guided and gene-guided approaches can be effective in targeted discovery of RiPPs from plants.

### 6.1. Bioactivity-guided discovery

Multiple plant RiPPs have been discovered from source plants through their bioactivity as described in the respective bioactivity sections of Section 3. The choice of source plants is often inspired by application of its extracts in herbal medicine such as kalata B1 as an oxytocic agent, stimulation of pain such as excelsatoxin A as Na_V_ channel modulator, antimicrobial activity against plant pathogens such as α/β-purothionins, or plant physiological activities such as RALFs.

Challenges of bioactivity-guided discovery can be time-consuming fractionation and rediscovery of known bioactive natural products. To this end, a pipeline called PepSAVI (statistically-guided bioactive peptides prioritized *via* mass spectrometry) was developed to correlate bioactivity of prefractionated plant extracts with the presence of peptide mass signals in liquid-chromatography mass spectrometry datasets of the extracts. The masses of candidate bioactive peptides identified by this pipeline enable dereplication by MS1 and tandem MS approaches that was validated by the characterization of cyclotide cyO2 as an antibacterial and cancer cell cytotoxic lead compound from a *Viola odorata* extract fraction library.^377^ The assay further enabled discovery of candidate bioactive cyclotides from *V. odorata* by improved tandem mass spectrometry-based sequencing of target peptides *via* ultraviolet photodissociation (UVPD) tandem mass spectrometry.^378^ Pipelines such as PepSAVI that connect high-throughput bioactivity assay data with mass spectrometry can rapidly dereplicate and identify new bioactive plant RiPPs.^379^ MS-based approaches such as molecular networking^380^ or Dereplicator+^381^ can enable rapid dereplication^382^ and discovery^383^ of bioactive lead compounds *via* spectral library comparison, for example within the Global Natural Product Molecular Networking platform,^384^ and they can further assess structural novelty by spectral library comparison^385^ or *de novo* structure elucidation.^386,387^

### 6.2. Structure-guided discovery

Many plant RiPPs were discovered based on physicochemical properties during natural product isolation or their structural features revealed by analytical chemistry techniques such as mass spectrometry (MS) as an initial chemotyping step.

Heveins were isolated from a centrifugation fraction including lutoids, vacuoles in latex-producing cells of rubber tree,^388^ and cliotides were discovered from heat stable extract fractions as compounds with high heat stability, a well characterized feature of cyclotides and CRPs.^389^ Besides physico-chemical features, plant RiPPs have been effectively characterized *via* mass spectrometry approaches. Structural information can be derived from a peptide analyte *via* tandem mass spectrometry experiments; however it can be challenging as many plant RiPPs have macrocyclic PTMs, which often prevents sequencing of a given peptide. Therefore, cysteine-rich peptides usually require disulfide bond reduction and *S*-alkylation before tandem mass spectrometry analysis to characterize the complete core peptide sequence, analogous to proteomic protocols for MS-based protein sequencing.^390–392^ The characteristic mass shift of iodoacetamide-alkylation at reduced cysteines (+57.0215 *m/z*) has been used for discovery of disulfide-containing plant RiPPs in plant extracts.^40,65,71,173,175,184,252,389^ A plant extract can be analyzed for the presence of analytes in the mass range of CRPs and cyclotides (2–6 kDa), treated for disulfide reduction and cysteine alkylation, then analyzed for alkylation-specific mass shifts in candidate peptides by comparison to untreated samples. For cyclotides, an additional step of proteolysis is usually required after disulfide reduction and cysteine alkylation to linearize the head-to-tail-macrocycle prior to tandem MS analysis. Proteolysis often involves GluC due to the presence of a conserved Glu in cyclotide core peptides^22^ or trypsin which can yield multiple tryptic core peptide species.^97^ Despite the efficacy of chemical and proteolytic processing of cyclotides and CRPs prior to tandem MS sequencing, the main disadvantage of this sample preparation is higher sample requirement, which can exclude low abundance peptides from discovery. Recently, ultraviolet photodissociation (UVPD) tandem mass spectrometry has been introduced to yield more fragmentation and therefore peptide sequencing data from cyclotides and CRPs in underivatized plant extract samples.^378^ In addition, collision-induced dissociation, which is the predominant tandem MS method in metabolomics, was further developed for MS fingerprinting *via* short sequence tags enabling cyclotide dereplication and prioritization for discovery.^393^ For burpitides, tandem mass spectrometry (MS/MS) can be applied for characterization of structural features to reveal the presence of a peptidic analyte and specific amino acids within the peptide sequence.^18,394^ In addition, tandem mass spectral data can be used in comparison to spectra of known plant peptides in custom or public databases for peptide classification.^384,395–397^ All aspects of peptide identification *via* MS/MS can inform novelty of a putative plant peptide and guide further experiments. Predicted amino acids and the size of the target analyte can enable the connection of a core peptide in a BURP-domain precursor peptide to the target spectrum. This approach has been applied effectively for the discovery of lyciumin-type and stephanotic acid-type burpitides, and cyclopeptide alkaloids. It can also be applied without a precursor peptide sequence as exemplified by the discovery of the glycosylated cyclopeptide alkaloid encephanine.^18^ The connection of tandem mass spectra of burpitides to their corresponding precursor peptides sequences has been implemented in the RiPP discovery platform HypoRiPPAtlas, which enables prediction of RiPP structures from candidate core peptide sequences in BURP-domain-containing precursor peptides. Subsequently, an algorithm called Dereplicator+^381^ is used to generate theoretical tandem mass spectra of the predicted RiPP structures and compare them to experimental tandem MS data for RiPP discovery. The HypoRiPPAtlas was able to identify several cyclopeptide alkaloids including cyclopeptide alkaloid elaeagnin (Fig. 8) and lyciumins as a proof-of-concept.^21^ Orbitides can be characterized by tandem MS in a similar fashion by comparison of tandem mass spectra to predicted precursor peptides identified in plant transcriptomes or by *de novo* sequencing.^398^ While mass spectrometry provides new plant RiPP candidates in structure-guided discovery approaches, NMR or crystallography are usually applied on scaled peptide samples to determine new structures of candidate peptides.^88,90^ With growing plant metabolomic databases, mass spectrometry-guided approaches will further drive plant RiPP discovery.

### 6.3. Gene-guided discovery

The characterization of precursor peptide genes (Table 1) and the growth of plant genetic resources^25,399–401^ has enabled the discovery of plant RiPPs through bioinformatic prediction of peptide chemotypes from plant genes.

Cyclotides were discovered in *Petunia* plants by searching for cyclotide precursor peptides in the EST database of the NCBI.^182^ Many CRPs have been predicted in plant transcriptomes by bioinformatic analyses, for instance, identification of a new thionin from *Papaver somniferum*.^190,402^ Similarly, genome mining and transcriptome analysis for fused burpitide precursor peptides has led to the discovery of new lyciumins, cyclopeptide alkaloids, and moroidins^17,19^ and split burpitide precursor peptides that resulted in discovery of new cyclopeptide alkaloids and hibispeptins.^20^ The characterization of a precursor peptide in any gene-guided discovery experiment yields a candidate core peptide sequence which can facilitate chemical characterization, *e.g.* by providing the long sequence information of cyclotides and CRPs. In addition, precursor peptides sequences can provide information about their processing through the secretory pathway *via* signal peptide prediction with SignalP.^403^ A challenge for gene-guided discovery of plant RiPPs is the identification of new precursor peptides and class-defining PTM enzymes in genomes with less biosynthetic gene clustering than in bacteria. A general approach to overcome this challenge is to search for core peptide sequences in predicted ORFs of peptide source plants^4,5,7,12–14,17,52^ or identify candidate precursor peptides based on genomic co-localization with scaffold-generating RiPP PTM genes.^20^ Finally, tissue-specific paired transcriptomics and metabolomics has proven to be an essential approach for elucidating pathways for which biosynthetic enzymes are not co-localized.^404^

## 7. Future directions

Plant peptides offer exciting research questions in terms of their discovery and biosynthesis and opportunities for further biotechnological and medicinal applications. Like microbial RiPP classes, the field of plant RiPPs is experiencing a gold rush in peptide discovery fueled by increasing genetic resources such as the 1KP database,^400^ the 10KP project,^25^ Phytozome^401^ and metabolomic databases such as GNPS.^384^ While many RiPPs have been predicted based on bioinformatic studies, an important direction is the integration of omics-datasets for automated plant RiPP discovery as it has been organized for microbial natural products^405^ and chemical characterization of predicted RiPPs. In addition, discovery of plant RiPPs will depend on identification of new precursor peptides such as fused and split burpitide precursor peptides. Given the localization of some RiPP core peptides within larger plant proteins, this identification might require larger theoretical databases to match candidate peptide spectral data to core peptides within proteins encoded in a plant transcriptome or genome. Regarding sequencing data, it will be important to further improve *de novo* assembly for generation of repetitive precursor genes. Regarding mass spectrometry, tandem mass spectrometry experiments could further improve to generate more core peptide sequence data for *de novo* or gene-aided structure elucidation. Remaining biosynthetic questions of plant RiPPs include elucidating the origin of known plant-sourced peptides such as bouvardins and whether non-ribosomal peptide biosynthesis exists within the plant kingdom. In terms of biosynthesis, the several processing proteases of head-to-tail-cyclic peptides are still unknown. For burpitides, structural and mechanistic insight into macrocyclizations by burpitide cyclases is lacking and major biosynthetic questions regarding processing of both split and fused burpitide precursor are unanswered including reactions involved in proteolysis and terminal core-peptide modifications. With the combination of rapidly developing genomic and biosynthetic insights, the future of plant RiPPs holds great promise of the discovery of new bioactive molecules, enzyme reactions, and engineering platforms.

## Figures and Tables

**Table 1:** Plant RiPP biosynthetic genes. Representative precursor peptides and PTM enzymes of plant RiPP classes are described in source organism, size, Pfam accession, GenBank accession and reference. Abbreviations: TIPTOP – two inhibitor peptide topologies precursor, aa – amino acid

Name	Organism	Length [aa]	RiPP class	Core peptide #	Pfam/InterPro accession^64^	GenBank accession	Reference
**Precursor peptides**							
Oak1	*Oldenlandia affinis*	124	Cyclotide	1	n/a	AAL05477	4
Oak2	*Oldenlandia affinis*	158	Cyclotide	2	n/a	AAL05478	4
Oak3	*Oldenlandia affinis*	111	Cyclotide	1	n/a	AAL05479	4
Oak4	*Oldenlandia affinis*	210	Cyclotide	3	n/a	AAL05480	4
TIPTOP1	*Momordica cochinchinensis*	281	Cyclotide, knottin	5	n/a	AEK70372	5
TIPTOP2	*Momordica cochinchinensis*	331	Cyclotide, knottin	5	n/a	AEK70373	5
TIPTOP3	*Momordica cochinchinensis*	431	Cyclotide, knottin	8	n/a	AEK70374	5
PawS1	*Helianthus annuus*	151	PawS-derived peptide	1	PF00234	ACT34883	52
PawS2	*Helianthus annuus*	137	PawS-derived peptide	1	PF00234	ACS74804	52
CterM precursor	*Clitoria ternatea*	127	Cyclotide	1	PF16720	AEB92229	22
PawL1a	*Senecio pinnatifolius* var. *maritimus*	175	Orbitide	2	PF00234	ARD06052	6
PawL1b	*Senecio pinnatifolius* var. *maritimus*	160	Orbitide	1	PF00234	ARD06067	6
Presegetalin A1	*Gypsophila vaccaria*	32	Orbitide	1	n/a	AEG75782	7
Linusorb A1–A3 precursor protein	*Linum usitatissimum*	219	Orbitide	5	n/a	FAA04139	8
Phytosulfokine precursor protein (PSK)	*Asparagus officinalis*	75	Phyto-sulfokines (linear RiPP)	1	PF06404	Q9FS10	9
CLAVATA3	*Arabidopsis thaliana*	96	CLE family peptides (linear RiPP)	1	n/a	NP_001118398	10
Systemin-like precursor proTOBSYS-A	*Nicotiana tabacum*	165	Systemins (linear RiPP)	2	n/a	AAK52096	11
Metallocarboxypeptidase inhibitor IIa precursor	*Solanum tuberosum*	102	CRP (knottin)	1	PF02977	NP_001275048	12
AcAMP-2 precursor	*Amaranthus caudatus*	86	CRP (6C hevein)	1	PF00187	P27275	13
Ginsentide precursor	*Panax ginseng*	121	CRP (8C hevein)	1	PF18687	AAX40471.1	65
Hevein precursor	*Hevea brasiliensis*	204	CRP (8C hevein)	1	PF00187, PF00967	AAA33357	14
EeCBP precursor	*Euonymus europaeus*	305	CRP (10C hevein)	1	PF00187, PF00182	AAP35270	16
WAMP-1a precursor	*Triticum kiharae*	116	CRP (10C hevein)	1	PF00187	P85966	66
EAFP1 precursor	*Eucommia ulmoides*	307	CRP (10C hevein)	1	PF00187, PF00182	WED30098	54
Defensin-like protein 1	*Triticum aestivum*	77	CRP (8C defensin)	1	PF00304	XP_044389906	67
Petunia defensin precursor	*Petunia × hybrida*	103	CRP (10C defensin)	1	PF00304	ADV59771	68
Crambin precursor	*Crambe hispanica* subsp. *abyssinica*	125	CRP (6C thionin)	1	PF00321	S52550	69
α1 purothionin precursor	*Triticum aestivum*	136	CRP (8C thionin)	1	PF00321	BAA12336	70
Jasmintide S1 precursor	*Jasminum sambac*	100	CRP (jasmintide)	1	n/a	ALO52196	71
Potentide pA1 precursor	*Potentilla anserina*	285	CRP (potentide)	4	n/a	XP_050382506	40
α-hairpinin EcAMP2 precursor	*Echinochloa crus-galli*	361	CRP (α-hairpinin)	3	PF14861	B3EWR6	41
IbAMP precursor	*Impatiens balsamina*	333	CRP (AMP)	6	n/a	O24006	42
Nodule-specific cysteine-rich peptide 169	*Medicago truncatula*	61	CRP (NCR)	1	PF07127	ABS31414	72
Snakin-1 precursor	*Solanum tuberosum*	88	CRP (snakin)	1	PF02704	CAC44032	37
RALF precursor	*Nicotiana tabacum*	115	CRP (RALF)	1	PF05498	AAL26478	73
LbaLycA	*Lycium barbarum*	543	Lyciumin-type RiPP (lyciumin)	12	PF03181	AYN06992	17
KjaBURP	*Kerria japonica*	380	Stephanotic acid-type RiPP (moroidin)	4	PF03181	QIG55799	19
SkrBURP	*Selaginella kraussiana*	525	Cyclopeptide alkaloid	8	PF03181	QXY82431	18
CcaBURP1	*Cercis canadensis*	630	Lyciumin-type RiPP (cercic acid)	16	PF03181	QXY82432	18
CcaBURP2	*Cercis canadensis*	458	Stephanotic acid-type RiPP (stephanotic acid)	8	PF03181	QXY82433	18
ElaBURP	*Elaeagnus pungens*	505	Cyclopeptide alkaloid	6	PF03181	OR257605	21
ArbA2	*Coffea arabica*	162	Cyclopeptide alkaloid	3	PF10950	XP_027066141	20
**PTM & processing enzymes**							
Butelase (asparaginyl endopeptidase/peptide macrocyclase)	*Clitoria ternatea*	482	Cyclotide	n/a	PF01650	AIB06797	74
*Oa*AEP1 (asparaginyl endopeptidase/peptide macrocyclase)	*Oldenlandia affinis*	474	Cyclotide	n/a	PF01650	ALG36103	75
*Oa*PDI	*Oldenlandia affinis*	531	Cyclotide	n/a	PF00085	ABS11216	59
*Oa*RD21a	*Oldenlandia affinis*	465	Cyclotide	n/a	PF00112	QBH22534	76
PCY1	*Gypsophila vaccaria*	724	Orbitide	n/a	n/a	AGL51088	77
Tyrosine sulfotransferase	*Arabidopsis thaliana*	500	Linear RiPP	n/a	PF03567	Q3EDG5	78
Hydroxyproline *O*-arabinosyltransferase	*Arabidopsis thaliana*	366	Linear RiPP	n/a	n/a	Q8W4E6	79
ArbB2	*Coffea arabica*	320	Cyclopeptide alkaloid	n/a	PF03181	XP_027066250	20

**Table 2:** Recommendations for burpitide definition and nomenclature. See main text for rationale and examples

Burpitide definition	
The macrocyclic bond derived from a BURP-domain-PTM is the primary class-defining feature of a burpitide	
In multicyclic burpitides, the macrocyclic bond, that is formed first during biosynthesis is the class-defining feature	
Ring size should not be a class-defining feature of a burpitide	
Terminal modifications should not be class-defining features if a macrocyclic BURP-PTM is present in a burpitide	
Burpitide classification	
Class	Class-defining PTM

Lyciumin-type peptides	Crosslink of Trp-indole-N to carbon in another amino acid side chain or peptide backbone
Cyclopeptide alkaloids	Crosslink of Tyr-phenol-O to carbon in another amino acid side chain
Stephanotic acid-type peptides	Crosslink of Trp-indole-C to carbon in another amino acid side chain
Hibispeptin-type peptides (proposed)	Crosslink of Tyr-phenol-C to carbon in another amino acid side chain
Burpitide nomenclature	
BURP-domain-containing proteins involved in a crosslinking step of a burpitide biosynthesis should be called burpitide cyclases	
Fused burpitide cyclases should be named with three letters referring to genus (1 letter) and species (2 letters) and ‘BURP’ (*e.g.* SkrBURP for *Selaginella kraussiana* burpitide cyclase)	
In split pathways, the burpitide cyclase should be designated with a B (*e.g.* ArbB2) and the precursor peptide should be designated with an A (*e.g.* ArbA2)	

## References

[1] Arnison PG, , Ribosomally synthesized and post-translationally modified peptide natural products: overview and recommendations for a universal nomenclature, Nat. Prod. Rep, 2013, 30, 108–160. 23165928 10.1039/c2np20085f PMC3954855

[2] Montalbán-López M, , New developments in RiPP discovery, enzymology and engineering, Nat. Prod. Rep, 2021, 38, 130–239. 32935693 10.1039/d0np00027b PMC7864896

[3] Tan N-H and Zhou J, Plant cyclopeptides, Chem. Rev, 2006, 106, 840–895. 16522011 10.1021/cr040699h

[4] Jennings C, West J, Waine C, Craik D and Anderson M, Biosynthesis and insecticidal properties of plant cyclotides: the cyclic knotted proteins from Oldenlandia affinis, Proc. Natl. Acad. Sci. U. S. A, 2001, 98, 10614–10619. 11535828 10.1073/pnas.191366898 PMC58514

[5] Mylne JS, , Cyclic peptides arising by evolutionary parallelism via asparaginyl-endopeptidase-mediated biosynthesis, Plant Cell, 2012, 24, 2765–2778. 22822203 10.1105/tpc.112.099085 PMC3426113

[6] Jayasena AS, , Stepwise evolution of a buried inhibitor peptide over 45 my, Mol. Biol. Evol, 2017, 34, 1505–1516. 28333296 10.1093/molbev/msx104

[7] Condie JA, , The biosynthesis of Caryophyllaceae-like cyclic peptides in Saponaria vaccaria L. from DNA-encoded precursors, Plant J, 2011, 67, 682–690. 21554452 10.1111/j.1365-313X.2011.04626.x

[8] Song Z, Burbridge C, Schneider DJ, Sharbel TF and Reaney MJT, The flax genome reveals orbitide diversity, BMC Genomics, 2022, 23, 534. 35870878 10.1186/s12864-022-08735-x PMC9308333

[9] Matsubayashi Y and Sakagami Y, Phytosulfokine, sulfated peptides that induce the proliferation of single mesophyll cells of Asparagus officinalis L, Proc. Natl. Acad. Sci. U. S. A, 1996, 93, 7623–7627. 8755525 10.1073/pnas.93.15.7623 PMC38796

[10] Fletcher JC, Brand U, Running MP, Simon R and Meyerowitz EM, Signaling of cell fate decisions by CLAVATA3 in Arabidopsis shoot meristems, Science, 1999, 283, 1911–1914. 10082464 10.1126/science.283.5409.1911

[11] Pearce G, Moura DS, Stratmann J and Ryan CA, Production of multiple plant hormones from a single polyprotein precursor, Nature, 2001, 411, 817–820. 11459063 10.1038/35081107

[12] Villanueva J, , Characterization of the wound-induced metallocarboxypeptidase inhibitor from potato1, FEBS Lett, 1998, 440, 175–182. 9862450 10.1016/s0014-5793(98)01447-1

[13] De Bolle MF, , Cloning and characterization of a cDNA encoding an antimicrobial chitin-binding protein from amaranth, Amaranthus caudatus, Plant Mol. Biol, 1993, 22, 1187–1190. 8400136 10.1007/BF00028991

[14] Broekaert I, Lee HI, Kush A, Chua NH and Raikhel N, Wound-induced accumulation of mRNA containing a hevein sequence in laticifers of rubber tree (Hevea brasiliensis), Proc. Natl. Acad. Sci. U. S. A, 1990, 87, 7633–7637. 2217194 10.1073/pnas.87.19.7633 PMC54802

[15] Kini SG, Wong KH, Tan WL, Xiao T and Tam JP, Morintides: cargo-free chitin-binding peptides from Moringa oleifera, BMC Plant Biol, 2017, 17, 68. 28359256 10.1186/s12870-017-1014-6 PMC5374622

[16] Van den Bergh KPB, , Synergistic antifungal activity of two chitin-binding proteins from spindle tree (Euonymus europaeus L.), Planta, 2004, 219, 221–232. 15048569 10.1007/s00425-004-1238-1

[17] Kersten RD and Weng J-K, Gene-guided discovery and engineering of branched cyclic peptides in plants, Proc. Natl. Acad. Sci. U. S. A, 2018, 115, E10961–E10969. 30373830 10.1073/pnas.1813993115 PMC6243238

[18] Chigumba DN, , Discovery and biosynthesis of cyclic plant peptides via autocatalytic cyclases, Nat. Chem. Biol, 2022, 18, 18–28. 34811516 10.1038/s41589-021-00892-6

[19] Kersten RD, , Gene-Guided Discovery and Ribosomal Biosynthesis of Moroidin Peptides, J. Am. Chem. Soc, 2022, 144, 7686–7692. 35438481 10.1021/jacs.2c00014

[20] Lima ST, , A widely distributed biosynthetic cassette is responsible for diverse plant side chain cross-linked cyclopeptides, Angew Chem. Int. Ed. Engl, 2023, 62, e202218082. 36529706 10.1002/anie.202218082 PMC10107690

[21] Lee Y-Y, , HypoRiPPAtlas as an Atlas of hypothetical natural products for mass spectrometry database search, Nat. Commun, 2023, 14, 4219. 37452020 10.1038/s41467-023-39905-4 PMC10349150

[22] Poth AG, Colgrave ML, Lyons RE, Daly NL and Craik DJ, Discovery of an unusual biosynthetic origin for circular proteins in legumes, Proc. Natl. Acad. Sci. U. S. A, 2011, 108, 10127–10132. 21593408 10.1073/pnas.1103660108 PMC3121837

[23] Oguis GK, Gilding EK, Jackson MA and Craik DJ, Butterfly pea (Clitoria ternatea), a cyclotide-bearing plant with applications in agriculture and medicine, Front. Plant Sci, 2019, 10, 645. 31191573 10.3389/fpls.2019.00645 PMC6546959

[24] Gründemann C, Stenberg KG and Gruber CW, T20K: An immunomodulatory cyclotide on its way to the clinic, Int J. Pept. Res. Ther, 2019, 25, 9–13.

[25] Cheng S, , 10KP: A phylodiverse genome sequencing plan, Gigascience, 2018, 7, 1–9. 10.1093/gigascience/giy013 PMC5869286 29618049

[26] Wang CKL, Kaas Q, Chiche L and Craik DJ, CyBase: a database of cyclic protein sequences and structures, with applications in protein discovery and engineering, Nucleic Acids Res, 2008, 36, D206–D210. 17986451 10.1093/nar/gkm953 PMC2239000

[27] Hammami R, Ben Hamida J, Vergoten G and Fliss I, PhytAMP: a database dedicated to antimicrobial plant peptides, Nucleic Acids Res, 2009, 37, D963–D968. 18836196 10.1093/nar/gkn655 PMC2686510

[28] Piel J, , Antitumor polyketide biosynthesis by an uncultivated bacterial symbiont of the marine sponge *Theonella swinhoei*, Proc. Natl. Acad. Sci. U. S. A, 2004, 101, 16222–16227. 15520376 10.1073/pnas.0405976101 PMC528957

[29] Tianero MD, Balaich JN and Donia MS, Localized production of defence chemicals by intracellular symbionts of Haliclona sponges, Nat. Microbiol, 2019, 4, 1149–1159. 30936484 10.1038/s41564-019-0415-8 PMC8647704

[30] Xu Y, , Bacterial biosynthesis and maturation of the didemnin anti-cancer agents, J. Am. Chem. Soc, 2012, 134, 8625–8632. 22458477 10.1021/ja301735a PMC3401512

[31] Crüsemann M, , Heterologous Expression, Biosynthetic Studies, and Ecological Function of the Selective Gq-Signaling Inhibitor FR900359, Angew. Chem., Int. Ed, 2018, 57, 836–840. 10.1002/anie.201707996 29194875

[32] Carlier A, , The genome analysis of Candidatus Burkholderia crenata reveals that secondary metabolism may be a key function of the Ardisia crenata leaf nodule symbiosis, Environ. Microbiol, 2016, 18, 2507–2522. 26663534 10.1111/1462-2920.13184

[33] Hermes C, König GM and Crüsemann M, The chromodepsins - chemistry, biology and biosynthesis of a selective Gq inhibitor natural product family, Nat. Prod. Rep, 2021, 38, 2276–2292. 33998635 10.1039/d1np00005e

[34] Hermes C, , Thioesterase-mediated side chain transesterification generates potent Gq signaling inhibitor FR900359, Nat. Commun, 2021, 12, 144. 33420046 10.1038/s41467-020-20418-3 PMC7794379

[35] Schlegel JG, , Macrocyclic Gq Protein Inhibitors FR900359 and/or YM-254890-Fit for Translation?, ACS Pharmacol. Transl. Sci, 2021, 4, 888–897. 33860209 10.1021/acsptsci.1c00021 PMC8033771

[36] Tschesche R, Elgamal M, Miana GA and Eckhardt G, Alkaloids from rhamnaceae—XXVI, Tetrahedron, 1975, 31, 2944–2947.

[37] Segura A, Moreno M, Madueño F, Molina A and García-Olmedo F, Snakin-1, a peptide from potato that is active against plant pathogens, Mol. Plant Microbe Interact, 1999, 12, 16–23. 9885189 10.1094/MPMI.1999.12.1.16

[38] Rubin GM and Ding Y, Recent advances in the biosynthesis of RiPPs from multicore-containing precursor peptides, J. Ind. Microbiol. Biotechnol, 2020, 47, 659–674. 32617877 10.1007/s10295-020-02289-1 PMC7666021

[39] Fisher MF, Zhang J, Berkowitz O, Whelan J and Mylne JS, Cyclic peptides in seed of Annona muricata are ribosomally synthesized, J. Nat. Prod, 2020, 83, 1167–1173. 32239926 10.1021/acs.jnatprod.9b01209

[40] Shen Y, , Potentides: New cysteine-rich peptides with unusual disulfide connectivity from *Potentilla anserina*, Chembiochem, 2019, 20, 1995–2004. 30927482 10.1002/cbic.201900127

[41] Ryazantsev DY, , A novel hairpin-like antimicrobial peptide from barnyard grass (Echinochloa crusgalli L.) seeds: Structure–functional and molecular-genetics characterization, Biochimie, 2014, 99, 63–70. 24275143 10.1016/j.biochi.2013.11.005

[42] Tailor RH, , A novel family of small cysteine-rich antimicrobial peptides from seed of impatiens balsaminaIs derived from a single precursor protein, J. Biol. Chem, 1997, 272, 24480–24487. 9305910 10.1074/jbc.272.39.24480

[43] Ding W, , Biosynthetic investigation of phomopsins reveals a widespread pathway for ribosomal natural products in Ascomycetes, Proc. Natl. Acad. Sci. U. S. A, 2016, 113, 3521–3526. 26979951 10.1073/pnas.1522907113 PMC4822579

[44] Ye Y, , Heterologous production of asperipin-2a: proposal for sequential oxidative macrocyclization by a fungi-specific DUF3328 oxidase, Org. Biomol. Chem, 2018, 17, 39–43. 30516224 10.1039/c8ob02824a

[45] Nagano N, , Class of cyclic ribosomal peptide synthetic genes in filamentous fungi, Fungal Genet. Biol, 2016, 86, 58–70. 26703898 10.1016/j.fgb.2015.12.010

[46] Umemura M, , Characterization of the biosynthetic gene cluster for the ribosomally synthesized cyclic peptide ustiloxin B in Aspergillus flavus, Fungal Genet. Biol, 2014, 68, 23–30. 24841822 10.1016/j.fgb.2014.04.011

[47] Imani AS, Lee AR, Vishwanathan N, de Waal F and Freeman MF, Diverse protein architectures and α-N-methylation patterns define split borosin RiPP biosynthetic gene clusters, ACS Chem. Biol, 2022, 17, 908–917. 35297605 10.1021/acschembio.1c01002 PMC9019853

[48] Quijano MR, , Distinct autocatalytic α- N-methylating precursors expand the borosin RiPP family of peptide natural products, J. Am. Chem. Soc, 2019, 141, 9637–9644. 31117659 10.1021/jacs.9b03690

[49] Schmidt EW, , Patellamide A and C biosynthesis by a microcin-like pathway in Prochloron didemni, the cyanobacterial symbiont of Lissoclinum patella, Proc. Natl. Acad. Sci. U. S. A, 2005, 102, 7315–7320. 15883371 10.1073/pnas.0501424102 PMC1091749

[50] Donia MS, , Natural combinatorial peptide libraries in cyanobacterial symbionts of marine ascidians, Nat. Chem. Biol, 2006, 2, 729–735. 17086177 10.1038/nchembio829

[51] Ziemert N, Ishida K, Liaimer A, Hertweck C and Dittmann E, Ribosomal synthesis of tricyclic depsipeptides in bloom-forming Cyanobacteria, Angew. Chem, 2008, 120, 7870–7873. 10.1002/anie.200802730 18683268

[52] Mylne JS, , Albumins and their processing machinery are hijacked for cyclic peptides in sunflower, Nat. Chem. Biol, 2011, 7, 257–259. 21423169 10.1038/nchembio.542

[53] Franke B, Mylne JS and Rosengren KJ, Buried treasure: biosynthesis, structures and applications of cyclic peptides hidden in seed storage albumins, Nat. Prod. Rep, 2018, 35, 137–146. 29379937 10.1039/c7np00066a

[54] Huang R-H, , Two novel antifungal peptides distinct with a five-disulfide motif from the bark of*Eucommia ulmoides*Oliv, FEBS Lett, 2002, 521, 87–90. 12067732 10.1016/s0014-5793(02)02829-6

[55] James AM, , Evidence for Ancient Origins of Bowman-Birk Inhibitors from Selaginella moellendorffii, Plant Cell, 2017, 29, 461–473. 28298518 10.1105/tpc.16.00831 PMC5385957

[56] Walsh CT, Garneau-Tsodikova S and Gatto GJ Jr, Protein posttranslational modifications: the chemistry of proteome diversifications, Angew Chem. Int. Ed. Engl, 2005, 44, 7342–7372. 16267872 10.1002/anie.200501023

[57] Aboye TL, , Interlocking disulfides in circular proteins: Toward efficient oxidative folding of cyclotides, Antioxid. Redox Signaling, 2011, 14, 77–86. 10.1089/ars.2010.3112 20486762

[58] Čemažar M, Zahariev S, Pongor S and Hore PJ, Oxidative folding of Amaranthus α-amylase inhibitor, J. Biol. Chem, 2004, 279, 16697–16705. 14749333 10.1074/jbc.M312328200

[59] Gruber CW, , A novel plant protein-disulfide isomerase involved in the oxidative folding of cystine knot defense proteins, J. Biol. Chem, 2007, 282, 20435–20446. 17522051 10.1074/jbc.M700018200

[60] Ramm S, , A self-sacrificing N-methyltransferase is the precursor of the fungal natural product omphalotin, Angew Chem. Int. Ed. Engl, 2017, 56, 9994–9997. 28715095 10.1002/anie.201703488

[61] van der Velden NS, , Autocatalytic backbone N-methylation in a family of ribosomal peptide natural products, Nat. Chem. Biol, 2017, 13, 833–835. 28581484 10.1038/nchembio.2393

[62] Gruis D, Schulze J and Jung R, Storage protein accumulation in the absence of the vacuolar processing enzyme family of cysteine proteases, Plant Cell, 2004, 16, 270–290. 14688293 10.1105/tpc.016378 PMC301410

[63] Shimada T, , Vacuolar processing enzymes are essential for proper processing of seed storage proteins in Arabidopsis thaliana, J. Biol. Chem, 2003, 278, 32292–32299. 12799370 10.1074/jbc.M305740200

[64] Paysan-Lafosse T, , InterPro in 2022, Nucleic Acids Res, 2023, 51, D418–D427. 36350672 10.1093/nar/gkac993 PMC9825450

[65] Tam JP, , Ginsentides: Cysteine and Glycine-rich peptides from the ginseng family with unusual disulfide connectivity, Sci. Rep, 2018, 8, 16201. 30385768 10.1038/s41598-018-33894-x PMC6212409

[66] Andreev YA, , Genes encoding hevein-like defense peptides in wheat: Distribution, evolution, and role in stress response, Biochimie, 2012, 94, 1009–1016. 22227377 10.1016/j.biochi.2011.12.023

[67] Colilla FJ, Rocher A and Mendez E, gamma-Purothionins: amino acid sequence of two polypeptides of a new family of thionins from wheat endosperm, FEBS Lett., 1990, 270, 191–194. 2226781 10.1016/0014-5793(90)81265-p

[68] Ghag SB, Shekhawat UKS and Ganapathi TR, Petunia floral defensins with unique prodomains as novel candidates for development of Fusarium wilt resistance in transgenic banana plants, PLoS One, 2012, 7, e39557. 22745785 10.1371/journal.pone.0039557 PMC3382125

[69] Schrader-Fischer G and Apel K, Organ-specific expression of highly divergent thionin variants that are distinct from the seed-specific crambin in the crucifer Crambe abyssinica, Mol. Gen. Genet, 1994, 245, 380–389. 7816048 10.1007/BF00290119

[70] Castagnaro A, Marana C, Carbonero P and Garcia-Olmedo F, CDNA cloning and nucleotide sequences of [alpha]1 and [alpha]2 thionins from hexaploid wheat endosperm, Plant Physiol, 1994, 106, 1221–1222. 7824649 10.1104/pp.106.3.1221 PMC159654

[71] Kumari G, , Cysteine-rich peptide family with unusual disulfide connectivity from *Jasminum sambac*, J. Nat. Prod, 2015, 78, 2791–2799. 26555361 10.1021/acs.jnatprod.5b00762

[72] Alunni B, , Genomic organization and evolutionary insights on *GRP* and *NCR* genes, two large nodule-specific gene families in *Medicago truncatula*, Mol. Plant Microbe Interact, 2007, 20, 1138–1148. 17849716 10.1094/MPMI-20-9-1138

[73] Pearce G, Moura DS, Stratmann J and Ryan CA Jr, RALF, a 5-kDa ubiquitous polypeptide in plants, arrests root growth and development, Proc. Natl. Acad. Sci. U. S. A, 2001, 98, 12843–12847. 11675511 10.1073/pnas.201416998 PMC60141

[74] Nguyen GKT, , Butelase 1 is an Asx-specific ligase enabling peptide macrocyclization and synthesis, Nat. Chem. Biol, 2014, 10, 732–738. 25038786 10.1038/nchembio.1586

[75] Harris KS, , Efficient backbone cyclization of linear peptides by a recombinant asparaginyl endopeptidase, Nat. Commun, 2015, 6, 10199. 26680698 10.1038/ncomms10199 PMC4703859

[76] Rehm FBH, , Papain-like cysteine proteases prepare plant cyclic peptide precursors for cyclization, Proc. Natl. Acad. Sci. U. S. A, 2019, 116, 7831–7836. 30944220 10.1073/pnas.1901807116 PMC6475389

[77] Barber CJS, , The two-step biosynthesis of cyclic peptides from linear precursors in a member of the plant family Caryophyllaceae involves cyclization by a serine protease-like enzyme, J. Biol. Chem, 2013, 288, 12500–12510. 23486480 10.1074/jbc.M112.437947 PMC3642298

[78] Komori R, Amano Y, Ogawa-Ohnishi M and Matsubayashi Y, Identification of tyrosylprotein sulfotransferase in Arabidopsis, Proc. Natl. Acad. Sci. U. S. A, 2009, 106, 15067–15072. 19666544 10.1073/pnas.0902801106 PMC2736448

[79] Ogawa-Ohnishi M, Matsushita W and Matsubayashi Y, Identification of three hydroxyproline O-arabinosyltransferases in Arabidopsis thaliana, Nat. Chem. Biol, 2013, 9, 726–730. 24036508 10.1038/nchembio.1351

[80] Gran L, An oxytocic principle found in Oldenlandia affinis DC, Medd. Nor. Farm. Selsk, 1970, 12, 80.

[81] Gran L, Oxytocic principles of Oldenlandia affinis, Lloydia, 1973, 36, 174–178. 4744554

[82] Grain L, Isolation of oxytocic peptides from Oldenlandia affinis by solvent extraction of tetraphenylborate complexes and chromatography on sephadex LH-20, Lloydia, 1973, 36, 207–208. 4744557

[83] Sletten K and Gran L, Some molecular properties of kalatapeptide B-1. A uterotonic polypeptide isolated from Oldenlandia affinis DC, Medd. Nor. Farm. Selsk, 1973, 7, 69–82.

[84] Mulvenna JP, Wang C and Craik DJ, CyBase: a database of cyclic protein sequence and structure, Nucleic Acids Res, 2006, 34, D192–D194. 16381843 10.1093/nar/gkj005 PMC1347368

[85] Weidmann J and Craik DJ, Discovery, structure, function, and applications of cyclotides: circular proteins from plants, J. Exp. Bot, 2016, 67, 4801–4812. 27222514 10.1093/jxb/erw210

[86] Veer SJ, Kan MW and Craik DJ, Cyclotides: from structure to function, Chem. Rev, 2019, 119, 12375–12421. 31829013 10.1021/acs.chemrev.9b00402

[87] Craik DJ, Daly NL, Bond T and Waine C, Plant cyclotides: A unique family of cyclic and knotted proteins that defines the cyclic cystine knot structural motif, J. Mol. Biol, 1999, 294, 1327–1336. 10600388 10.1006/jmbi.1999.3383

[88] Saether O, , Elucidation of the primary and three-dimensional structure of the uterotonic polypeptide kalata B1, Biochemistry, 1995, 34, 4147–4158. 7703226 10.1021/bi00013a002

[89] Tam JP, Lu YA, Yang JL and Chiu KW, An unusual structural motif of antimicrobial peptides containing end-to-end macrocycle and cystine-knot disulfides, Proc. Natl. Acad. Sci. U. S. A, 1999, 96, 8913–8918. 10430870 10.1073/pnas.96.16.8913 PMC17707

[90] Rees DC and Lipscomb WN, Refined crystal structure of the potato inhibitor complex of carboxypeptidase A at 2.5 Å resolution, J. Mol. Biol, 1982, 160, 475–498. 7154070 10.1016/0022-2836(82)90309-6

[91] Colgrave ML and Craik DJ, Thermal, chemical, and enzymatic stability of the cyclotide kalata B1: the importance of the cyclic cystine knot, Biochemistry, 2004, 43, 5965–5975. 15147180 10.1021/bi049711q

[92] Ravipati AS, , Lysine-rich cyclotides: A new subclass of circular knotted proteins from Violaceae, ACS Chem. Biol, 2015, 10, 2491–2500. 26322745 10.1021/acschembio.5b00454

[93] Dutton JL, , Conserved structural and sequence elements implicated in the processing of gene-encoded circular proteins, J. Biol. Chem, 2004, 279, 46858–46867. 15328347 10.1074/jbc.M407421200

[94] Mulvenna JP, Sando L and Craik DJ, Processing of a 22 kDa precursor protein to produce the circular protein tricyclon A, Structure, 2005, 13, 691–701. 15893660 10.1016/j.str.2005.02.013

[95] Simonsen SM, , A continent of plant defense peptide diversity: cyclotides in Australian Hybanthus (Violaceae), Plant Cell, 2005, 17, 3176–3189. 16199617 10.1105/tpc.105.034678 PMC1276036

[96] Poth AG, , Discovery of cyclotides in the fabaceae plant family provides new insights into the cyclization, evolution, and distribution of circular proteins, ACS Chem. Biol, 2011, 6, 345–355. 21194241 10.1021/cb100388j

[97] Nguyen GKT, , Discovery and characterization of novel cyclotides originated from chimeric precursors consisting of albumin-1 chain a and cyclotide domains in the Fabaceae family,J. Biol. Chem, 2011, 286, 24275–24287. 21596752 10.1074/jbc.M111.229922 PMC3129208

[98] Yang R, , Engineering a catalytically efficient recombinant protein ligase, J. Am. Chem. Soc, 2017, 139, 5351–5358. 28199119 10.1021/jacs.6b12637

[99] Jackson M, , Molecular basis for the production of cyclic peptides by the plant asparaginyl endopeptidases, Nat. Commun, 2018, 1–12. 29925835 10.1038/s41467-018-04669-9 PMC6010433

[100] Du J, , A bifunctional asparaginyl endopeptidase efficiently catalyzes both cleavage and cyclization of cyclic trypsin inhibitors, Nat. Commun, 2020, 11, 1575. 32221295 10.1038/s41467-020-15418-2 PMC7101308

[101] Barbeta BL, Marshall AT, Gillon AD, Craik DJ and Anderson MA, Plant cyclotides disrupt epithelial cells in the midgut of lepidopteran larvae, Proc. Natl. Acad. Sci. U. S. A, 2008, 105, 1221–1225. 18202177 10.1073/pnas.0710338104 PMC2234119

[102] Pinto MFS, , Identification and structural characterization of novel cyclotide with activity against an insect pest of sugar cane, J. Biol. Chem, 2012, 287,134–147. 22074926 10.1074/jbc.M111.294009 PMC3249065

[103] Plan MRR, Saska I, Cagauan AG and Craik DJ, Backbone cyclised peptides from plants show molluscicidal activity against the rice pest Pomacea canaliculata (golden apple snail), J. Agric. Food Chem, 2008, 56, 5237–5241. 18557620 10.1021/jf800302f

[104] Colgrave ML, , Cyclotides: natural, circular plant peptides that possess significant activity against gastrointestinal nematode parasites of sheep, Biochemistry, 2008, 47, 5581–5589. 18426225 10.1021/bi800223y

[105] Colgrave ML, , Anthelmintic activity of cyclotides: In vitro studies with canine and human hookworms, Acta Trop, 2009, 109, 163–166. 19059189 10.1016/j.actatropica.2008.11.003

[106] Lindholm P, , Cyclotides: a novel type of cytotoxic agents, Mol. Cancer Ther, 2002, 1, 365–369. 12477048

[107] Svangård E, , Cytotoxic cyclotides from Viola tricolor, J. Nat. Prod, 2004, 67, 144–147. 14987049 10.1021/np030101l

[108] Herrmann A, , The alpine violet, Viola biflora, is a rich source of cyclotides with potent cytotoxicity, Phytochemistry, 2008, 69, 939–952. 18191970 10.1016/j.phytochem.2007.10.023

[109] Gerlach SL, Burman R, Bohlin L, Mondal D and Göransson U, Isolation, characterization, and bioactivity of cyclotides from the Micronesian plant Psychotria leptothyrsa, J. Nat. Prod, 2010, 73, 1207–1213. 20575512 10.1021/np9007365

[110] Yeshak MY, Burman R, Asres K and Göransson U, Cyclotides from an extreme habitat: characterization of cyclic peptides from Viola abyssinica of the Ethiopian highlands, J. Nat. Prod, 2011, 74, 727–731. 21434649 10.1021/np100790f

[111] Sen Z, Zhan XK, Jing J, Yi Z and Wanqi Z, Chemosensitizing activities of cyclotides from Clitoria ternatea in paclitaxel-resistant lung cancer cells, Oncol. Lett, 2013, 5, 641–644. 23419988 10.3892/ol.2012.1042 PMC3573133

[112] Esmaeili MA, , Viola plant cyclotide vigno 5 induces mitochondria-mediated apoptosis via cytochrome C release and caspases activation in cervical cancer cells, Fitoterapia, 2016, 109, 162–168. 26751970 10.1016/j.fitote.2015.12.021

[113] Pinto MEF, , Inhibition of breast cancer cell migration by cyclotides isolated from Pombalia calceolaria, J. Nat. Prod, 2018, 81, 1203–1208. 29757646 10.1021/acs.jnatprod.7b00969 PMC5974699

[114] Ding X, Bai D and Qian J, Novel cyclotides from Hedyotis biflora inhibit proliferation and migration of pancreatic cancer cell in vitro and in vivo, Med. Chem. Res, 2014, 23, 1406–1413.

[115] Pränting M, Lööv C, Burman R, Göransson U and Andersson DI, The cyclotide cycloviolacin O2 from Viola odorata has potent bactericidal activity against Gramnegative bacteria, J. Antimicrob. Chemother, 2010, 65, 1964–1971. 20558471 10.1093/jac/dkq220

[116] Zarrabi M, Dalirfardouei R, Sepehrizade Z and Kermanshahi RK, Comparison of the antimicrobial effects of semipurified cyclotides from Iranian Viola odorata against some of plant and human pathogenic bacteria, J. Appl. Microbiol, 2013, 115, 367–375. 23800264 10.1111/jam.12251

[117] Narayani M, Chadha A and Srivastava S, Callus and cell suspension culture of Viola odorata as in vitro production platforms of known and novel cyclotides, Plant Cell, Tissue Organ Cult., 2017, 130, 289–299.

[118] Strömstedt AA, Park S, Burman R and Göransson U, Bactericidal activity of cyclotides where phosphatidylethanolamine-lipid selectivity determines antimicrobial spectra, Biochim. Biophys. Acta, Biomembr, 2017, 1859, 1986–2000. 28669767 10.1016/j.bbamem.2017.06.018

[119] Gustafson KR, , Circulins A and B. Novel human immunodeficiency virus (HIV)-inhibitory macrocyclic peptides from the tropical tree Chassalia parvifolia, J. Am. Chem. Soc, 1994, 116, 9337–9338.

[120] Hernandez J-F, , Squash trypsin inhibitors from Momordica cochinchinensis exhibit an atypical macrocyclic structure, Biochemistry, 2000, 39, 5722–5730. 10801322 10.1021/bi9929756

[121] Kaufmann HP and Tobschirbel A, Über ein Oligopeptid aus Leinsamen, Chem. Ber, 1959, 92, 2805–2809.

[122] Fisher MF, , The genetic origin of evolidine, the first cyclopeptide discovered in plants, and related orbitides, J. Biol. Chem, 2020, 295, 14510–14521. 32817170 10.1074/jbc.RA120.014781 PMC7573267

[123] Fisher MF, , A family of small, cyclic peptides buried in preproalbumin since the Eocene epoch, Plant Direct, 2018, 2, e00042. 30417166 10.1002/pld3.42 PMC6223261

[124] Elliott AG, , Evolutionary origins of a bioactive peptide buried within Preproalbumin, Plant Cell, 2014, 26, 981–995. 24681618 10.1105/tpc.114.123620 PMC4001405

[125] Okinyo-Owiti DP, Young L, Burnett P-GG and Reaney MJT, New flaxseed orbitides: Detection, sequencing, and ^15^ N incorporation, Biopolymers, 2014, 102, 168–175. 24408479 10.1002/bip.22459

[126] Belknap WR, , A family of small cyclic amphipathic peptides (SCAmpPs) genes in citrus, BMC Genomics, 2015, 16, 303. 25887227 10.1186/s12864-015-1486-4 PMC4409773

[127] Fisher MF, Payne CD, Rosengren KJ and Mylne JS, An orbitide from Ratibida columnifera seed containing 16 amino acid residues, J. Nat. Prod, 2019, 82, 2152–2158. 31392883 10.1021/acs.jnatprod.9b00111

[128] Zhao Y-R, , Cyclopeptides from Stellaria yunnanensis, Phytochemistry, 1995, 40, 1453–1456. 8534403 10.1016/0031-9422(95)00351-7

[129] Picur B, Lisowski M and Siemion IZ, A new cyclolinopeptide containing nonproteinaceous amino acidN-methyl-4-aminoproline, Lett. Pept. Sci, 1998, 5, 183–187.

[130] Chekan JR, Estrada P, Covello PS and Nair SK, Characterization of the macrocyclase involved in the biosynthesis of RiPP cyclic peptides in plants, Proc. Natl. Acad. Sci. U. S. A, 2017, 114, 6551–6556. 28584123 10.1073/pnas.1620499114 PMC5488928

[131] Ludewig H, , Characterization of the fast and promiscuous macrocyclase from plant PCY1 enables the use of simple substrates, ACS Chem. Biol, 2018, 13, 801–811. 29377663 10.1021/acschembio.8b00050 PMC5859912

[132] Gui B, , Identification and quantification of cyclolinopeptides in five flaxseed cultivars, J. Agric. Food Chem, 2012, 60, 8571–8579. 22897677 10.1021/jf301847u

[133] Burnett P-GG, Jadhav PD, Okinyo-Owiti DP, Poth AG and Reaney MJT, Glycine-containing flaxseed orbitides, J. Nat. Prod, 2015, 78, 681–688. 25781981 10.1021/np5008558

[134] Wieczorek Z, Bengtsson B, Trojnar J and Siemion IZ, Immunosuppressive activity of cyclolinopeptide A, Pept. Res, 1991, 4, 275–283. 1802239

[135] Siemion IZ, Pçdyczak A, Trojnar J, Zimecki M and Wieczorek Z, Immunosuppressive activity of antamanide and some of its analogues, Peptides, 1992, 13, 1233–1237. 1494502 10.1016/0196-9781(92)90034-z

[136] Gaymes TJ, Cebrat M, Siemion IZ and Kay JE, Cyclolinopeptide A (CLA) mediates its immunosuppressive activity through cyclophilin-dependent calcineurin inactivation, FEBS Lett, 1997, 418, 224–227. 9414131 10.1016/s0014-5793(97)01345-8

[137] Cebrat M, Wieczorek Z and Siemion IZ, Immunosuppressive activity of hymenistatin I, Peptides, 1996, 17, 191–196. 8801520 10.1016/0196-9781(95)02123-x

[138] Morita H, , Solution state conformation of animmunosuppressive cyclic dodecapeptide, cycloleonurinin, Tetrahedron, 1997, 53, 7469–7478.

[139] Morita H, Yun YS, Takeya K, Itokawa H and Yamada K, Segetalins B, C and D, three new cyclic peptides from Vaccaria segetalis, Tetrahedron, 1995, 51, 6003–6014.

[140] Itokawa H, Yun Y, Morita H, Takeya K and Yamada K, Estrogen-like activity of cyclic peptides from Vaccaria segetalisextracts^1^, Planta Med, 1995, 61, 561–562. 8824953 10.1055/s-2006-959373

[141] Yun YS, Morita H, Takeya K and Itokawa H, Cyclic peptides from higher plants. 34. Segetalins G and H, structures and estrogen-like activity of cyclic pentapeptides from *Vaccaria segetalis*, J. Nat. Prod, 1997, 60, 216–218. 9157189 10.1021/np960617n

[142] Poojary B and Belagali SL, Synthesis, characterizationand biological evaluation of cyclic peptides: Viscumamide, yunnanin A and evolidine, ChemInform, 2006, 37, 1313–1320.

[143] Chuang P-H, , Cyclopeptides with Anti-inflammatory Activity from Seeds of *Annona montana*, J. Nat. Prod, 2008, 71, 1365–1370. 18687006 10.1021/np8001282

[144] Zou X-G, , Flaxseed orbitides, linusorbs, inhibit LPS-induced THP-1 macrophage inflammation, RSC Adv., 2020, 10, 22622–22630. 35514549 10.1039/c9ra09058d PMC9054600

[145] Wele A, Zhang Y, Dubost L, Pousset J-L and Bodo B, Cyclic peptides from the seeds of Annona glauca and A. cherimola, Chem. Pharm. Bull, 2006, 54, 690–692. 10.1248/cpb.54.690 16651768

[146] Morita H, Kayashita T, Shimomura M, Takeya K and Itokawa H, Cyclic peptides from higher plants. 24. Yunnanin C, a novel cyclic heptapeptide from *Stellaria yunnanensis*, J. Nat. Prod, 1996, 59, 280–282. 8882429 10.1021/np960123q

[147] Zou X-G, , iCellular uptake of [1–9-NαC]-linusorb B2 and [1–9-NαC]-linusorb B3 isolated from flaxseed, and their antitumor activities in human gastric SGC-7901 cells, J. Funct. Foods, 2018, 48, 692–703.

[148] Zou X-G, , [1–9-NαC]-linusorb B2 and [1–9-NαC]-linusorb B3 isolated from flaxseed induce G1 cell cycle arrest on SGC-7901 cells by modulating the AKT/JNK signaling pathway, J. Funct. Foods, 2019, 52, 332–339.

[149] Sung NY, , The anti-cancer effect of linusorb B3 from flaxseed oil through the promotion of apoptosis, inhibition of actin polymerization, and suppression of Src activity in glioblastoma cells, Molecules, 2020, 25, 5881. 33322712 10.3390/molecules25245881 PMC7764463

[150] Morita H, , Structure of a new cyclic nonapeptide, segetalin F, and vasorelaxant activity of segetalins from Vaccaria segetalis, Bioorg. Med. Chem. Lett, 2006, 16, 4458–4461. 16844371 10.1016/j.bmcl.2006.06.083

[151] Pinto MEF, , Ribifolin, an orbitide from *Jatropha ribifolia*, and its potential antimalarial activity, J. Nat. Prod, 2015, 78, 374–380. 25699574 10.1021/np5007668

[152] Luckett S, , High-resolution structure of a potent, cyclic proteinase inhibitor from sunflower seeds, J. Mol. Biol, 1999, 290, 525–533. 10390350 10.1006/jmbi.1999.2891

[153] Jayasena AS, , Next generation sequencing and de novo transcriptomics to study gene evolution, Plant Methods, 2014, 10, 34. 25364374 10.1186/1746-4811-10-34 PMC4216380

[154] Veer SJ, White AM and Craik DJ, Sunflower trypsin inhibitor-1 (SFTI-1): Sowing seeds in the fields of chemistry and biology, Angew Chem. Int. Ed. Engl, 2021, 60, 8050–8071. 32621554 10.1002/anie.202006919

[155] Gitlin-Domagalska A, Maciejewska A and Dębowski D, Bowman-Birk inhibitors: Insights into family of multifunctional proteins and peptides with potential therapeutical applications, Pharmaceuticals, 2020, 13, 421. 33255583 10.3390/ph13120421 PMC7760496

[156] Franke B, , Two proteins for the price of one: Structural studies of the dual-destiny protein preproalbumin with sunflower trypsin inhibitor-1, J. Biol. Chem, 2017, 292, 12398–12411. 28536266 10.1074/jbc.M117.776955 PMC5535016

[157] Shewry PR and Pandya MJ, The 2S albumin storage proteins, in Seed Proteins, Springer, Netherlands, 1999, pp. 563–586.

[158] Mylne JS, Hara-Nishimura I and Rosengren KJ, Seed storage albumins: biosynthesis, trafficking and structures, Funct. Plant Biol, 2014, 41, 671. 32481022 10.1071/FP14035

[159] Bernath-Levin K, , Peptide macrocyclization by a bifunctional endoprotease, Chem. Biol, 2015, 22, 571–582. 25960260 10.1016/j.chembiol.2015.04.010

[160] Haywood J, , Structural basis of ribosomal peptide macrocyclization in plants, Elife, 2018, 7, e32955. 29384475 10.7554/eLife.32955 PMC5834244

[161] Marx UC, , Enzymatic cyclization of a potent bowman-Birk protease inhibitor, sunflower trypsin inhibitor-1, and solution structure of an acyclic precursor peptide, J. Biol. Chem, 2003, 278, 21782–21789. 12621047 10.1074/jbc.M212996200

[162] Broekaert WF, , Antimicrobial peptides from plants, CRC Crit. Rev. Plant Sci, 1997, 16, 297–323.

[163] Slavokhotova AA, Shelenkov AA, Andreev YA and Odintsova TI, Hevein-like antimicrobial peptides of plants, Biochemistry, 2017, 82, 1659–1674. 29523064 10.1134/S0006297917130065

[164] Kintzing JR and Cochran JR, Engineered knottin peptides as diagnostics, therapeutics, and drug delivery vehicles, Curr. Opin. Chem. Biol, 2016, 34, 143–150. 27642714 10.1016/j.cbpa.2016.08.022

[165] Clore GM, Gronenborn AM, Nilges M and Ryan CA, Three-dimensional structure of potato carboxypeptidase inhibitor in solution. A study using nuclear magnetic resonance, distance geometry, and restrained molecular dynamics, Biochemistry, 1987, 26, 8012–8023. 3427120 10.1021/bi00398a069

[166] Le Nguyen D, , Molecular recognition between serine proteases and new bioactive microproteins with a knotted structure, Biochimie, 1990, 72, 431–435. 2124146 10.1016/0300-9084(90)90067-q

[167] Clore GM, , The three-dimensional structure of α1-purothionin in solution: combined use of nuclear magnetic resonance, distance geometry and restrained molecular dynamics, EMBO J, 1986, 5, 2729–2735. 16453716 10.1002/j.1460-2075.1986.tb04557.x PMC1167175

[168] Bruix M, et al., Solution structure of .gamma.1-H and .gamma.1-P thionins from barley and wheat endosperm determined by proton NMR: a structural motif common to toxic arthropod proteins, Biochemistry, 1993, 32, 715–724. 8380707 10.1021/bi00053a041

[169] Yeung H, , Radiation damage and racemic protein crystallography reveal the unique structure of the GASA/snakin protein superfamily, Angew Chem. Int. Ed. Engl, 2016, 55, 7930–7933. 27145301 10.1002/anie.201602719

[170] Van Parijs J, Broekaert WF, Goldstein IJ and Peumans WJ, Hevein: an antifungal protein from rubber-tree (Hevea brasiliensis) latex, Planta, 1991, 183, 258–264. 24193629 10.1007/BF00197797

[171] Rodríguez-Romero A, Ravichandran KG and Soriano-García M, Crystal structure of hevein at 2.8 Å resolution, FEBS Lett, 1991, 291, 307–309. 1936279 10.1016/0014-5793(91)81308-u

[172] Nolde SB, , Disulfide-stabilized helical hairpin structure and activity of a novel antifungal peptide EcAMP1 from seeds of barnyard grass (Echinochloa crusgalli), J. Biol. Chem, 2011, 286, 25145–25153. 21561864 10.1074/jbc.M110.200378 PMC3137087

[173] Wong KH, , Ginkgotides: Proline-Rich Hevein-Like Peptides from Gymnosperm Ginkgo biloba, Front. Plant Sci, 2016, 7, 1639. 27857717 10.3389/fpls.2016.01639 PMC5093130

[174] Kumari G, , Molecular diversity and function of jasmintides from Jasminum sambac, BMC Plant Biol., 2018, 18, 144. 29996766 10.1186/s12870-018-1361-y PMC6042386

[175] Tan WL, , Lybatides from Lycium barbarum contain an unusual cystine-stapled helical peptide scaffold, Sci. Rep, 2017, 7, 5194. 28701689 10.1038/s41598-017-05037-1 PMC5507927

[176] Isozumi N, , Structure and antimicrobial activity of NCR169, a nodule-specific cysteine-rich peptide of Medicago truncatula, Sci. Rep, 2021, 11, 9923. 33972675 10.1038/s41598-021-89485-w PMC8110993

[177] Kader J-C, Lipid-transfer proteins in plants, Annu. Rev. Plant Physiol. Plant Mol. Biol, 1996, 47, 627–654. 15012303 10.1146/annurev.arplant.47.1.627

[178] Pallaghy PK, Nielsen KJ, Craik DJ and Norton RS, A common structural motif incorporating a cystine knot and a triple-stranded beta-sheet in toxic and inhibitory polypeptides, Protein Sci, 1994, 3, 1833–1839. 7849598 10.1002/pro.5560031022 PMC2142598

[179] Ryan CA, Inhibition of carboxypeptidase A by a naturally occurring polypeptide from potatoes, Biochem. Biophys. Res. Commun, 1971, 44, 1265–1270. 5160409 10.1016/s0006-291x(71)80222-x

[180] Krätzner R, , Structure of Ecballium elaterium trypsin inhibitor II (EETI-II): a rigid molecular scaffold, Acta Crystallogr., Sect. D: Biol. Crystallogr, 2005, 61, 1255–1262. 16131759 10.1107/S0907444905021207

[181] Jouvensal L, , PA1b, an insecticidal protein extracted from pea seeds (*Pisum sativum*): ^1^H-2-D NMR study and molecular modeling, Biochemistry, 2003, 42, 11915–11923. 14556622 10.1021/bi034803l

[182] Poth AG, , Cyclotides associate with leaf vasculature and are the products of a novel precursor in petunia (Solanaceae), J. Biol. Chem, 2012, 287, 27033–27046. 22700981 10.1074/jbc.M112.370841 PMC3411041

[183] Watanabe Y, , A peptide that stimulates phosphorylation of the plant insulin-binding protein. Isolation, primary structure and cDNA cloning, Eur. J. Biochem, 1994, 224, 167–172. 8076638 10.1111/j.1432-1033.1994.tb20008.x

[184] Huang J, , Astratides: Insulin-Modulating, Insecticidal, and Antifungal Cysteine-Rich Peptides from Astragalus membranaceus, J. Nat. Prod, 2019, 82,194–204. 30758201 10.1021/acs.jnatprod.8b00521

[185] Nguyen PQT, , Antiviral Cystine Knot α-Amylase Inhibitors from Alstonia scholaris, J. Biol. Chem, 2015, 290, 31138–31150. 26546678 10.1074/jbc.M115.654855 PMC4692237

[186] Nguyen GKT, Lim WH, Nguyen PQT and Tam JP, Novel cyclotides and uncyclotides with highly shortened precursors from Chassalia chartacea and effects of methionine oxidation on bioactivities, J. Biol. Chem, 2012, 287, 17598–17607. 22467870 10.1074/jbc.M111.338970 PMC3366795

[187] Nguyen GKT, , Discovery of a linear cyclotide from the bracelet subfamily and its disulfide mapping by top-down mass spectrometry, J. Biol. Chem, 2011, 286, 44833–44844. 21979955 10.1074/jbc.M111.290296 PMC3247958

[188] Nguyen GKT, , Discovery of linear cyclotides in monocot plant Panicum laxum of Poaceae family provides new insights into evolution and distribution of cyclotides in plants, J. Biol. Chem, 2013, 288, 3370–3380. 23195955 10.1074/jbc.M112.415356 PMC3561556

[189] Bohlmann H and Apel K, Thionins, Annu. Rev. Plant Physiol. Plant Mol. Biol, 1991, 42, 227–240.

[190] Höng K, Austerlitz T, Bohlmann T and Bohlmann H, The thionin family of antimicrobial peptides, PLoS One, 2021, 16, e0254549. 34260649 10.1371/journal.pone.0254549 PMC8279376

[191] Castagnaro A, Maraña C, Carbonero P and García-Olmedo F, Extreme divergence of a novel wheat thionin generated by a mutational burst specifically affecting the mature protein domain of the precursor, J. Mol. Biol, 1992, 224, 1003–1009. 1569564 10.1016/0022-2836(92)90465-v

[192] Balls AK, A crystalline sulphur-protein from wheat, J. Wash. Acad. Sci, 1942, 32, 132–137.

[193] Rao U, Stec B and Teeter MM, Refinement of purothionins reveals solute particles important for lattice formation and toxicity. Part 1: α_1_-purothionin revisited, Acta Crystallogr., Sect. D: Biol. Crystallogr, 1995, 51, 904–913. 15299760 10.1107/S0907444995002964

[194] Vanetten CH, Nielsen HC and Peters JE, A crystalline polypeptide from the seed of Crambe abyssinica, Phytochemistry, 1965, 4, 467–473.

[195] Teeter MM, Mazer JA and L’Italien JJ, Primary structure of the hydrophobic plant protein crambin, Biochemistry, 1981, 20, 5437–5443. 6895315 10.1021/bi00522a013

[196] Yamano A, Heo N-H and Teeter MM, Crystal structure of Ser-22/ile-25 form crambin confirms solvent, side chain substate correlations, J. Biol. Chem, 1997, 272, 9597–9600. 9092482 10.1074/jbc.272.15.9597

[197] Fujimura M, Ideguchi M, Minami Y, Watanabe K and Tadera K, Purification, characterization, and sequencing of novel antimicrobial peptides, *Tu*-AMP 1 and*Tu*-AMP 2, from bulbs of tulip (*Tulipa gesnerianaL*.), Biosci., Biotechnol., Biochem, 2004, 68, 571–577. 15056889 10.1271/bbb.68.571

[198] Milbradt AG, Kerek F, Moroder L and Renner C, Structural characterization of hellethionins from *Helleborus purpurascens*, Biochemistry, 2003, 42, 2404–2411. 12600207 10.1021/bi020628h

[199] Samuelsson G, Seger L and Olson T, The amino acid sequence of oxidized viscotoxin A3 from the European mistletoe (Viscum album L, Loranthaceae), Acta Chem. Scand, 1968, 22, 2624–2642. 5719166 10.3891/acta.chem.scand.22-2624

[200] Schrader G and Apel K, Isolation and characterization of cDNAs encoding viscotoxins of mistletoe (Viscum album), Eur. J. Biochem, 1991, 198, 549–553. 1710983 10.1111/j.1432-1033.1991.tb16049.x

[201] Mellstrand ST and Samuelsson G, Phoratoxin, a Toxic Protein from the Mistletoe Phoradendron tomentosum subsp. macrophyllum (Loranthaceae). Improvements in the Isolation Procedure and Further Studies on the Properties, Eur. J. Biochem, 1973, 32, 143–147. 4687388 10.1111/j.1432-1033.1973.tb02590.x

[202] Li S-S, , Ligatoxin B, a new cytotoxic protein with a novel helix–turn-helix DNA-binding domain from the mistletoe Phoradendron liga, Biochem. J, 2002, 366, 405–413. 12049612 10.1042/BJ20020221 PMC1222806

[203] Shafee T and Anderson MA, A quantitative map of protein sequence space for the cis-defensin superfamily, Bioinformatics, 2019, 35, 743–752. 30102339 10.1093/bioinformatics/bty697

[204] Ming D and Hellekant G, Brazzein, a new high-potency thermostable sweet protein from Pentadiplandra brazzeana B, FEBS Lett., 1994, 355, 106–108. 7957951 10.1016/0014-5793(94)01184-2

[205] De-Paula VS, , Evolutionary relationship between defensins in the Poaceae family strengthened by the characterization of new sugarcane defensins, Plant Mol. Biol, 2008, 68, 321–335. 18618271 10.1007/s11103-008-9372-y

[206] Dias R. d. O. and Franco OL, Cysteine-stabilized αβ defensins: From a common fold to antibacterial activity, Peptides, 2015, 72, 64–72. 25929172 10.1016/j.peptides.2015.04.017

[207] Thevissen K, , DmAMP1, an antifungal plant defensin from dahlia (*Dahlia merckii*), interacts with sphingolipids from*Saccharomyces cerevisiae*, FEMS Microbiol. Lett, 2003, 226, 169–173. 13129623 10.1016/S0378-1097(03)00590-1

[208] Fant F, Vranken W, Broekaert W and Borremans F, Determination of the three-dimensional solution structure of Raphanus sativus Antifungal Protein 1 by 1 H NMR 1 1Edited by P. E. Wright, J. Mol. Biol, 1998, 279, 257–270. 9636715 10.1006/jmbi.1998.1767

[209] Terras FR, , Analysis of two novel classes of plant antifungal proteins from radish (Raphanus sativus L.) seeds, J. Biol. Chem, 1992, 267, 15301–15309. 1639777

[210] Zhao Q, Chae YK and Markley JL, NMR solution structure of ATT_p_, an *Arabidopsis thaliana* trypsin inhibitor, Biochemistry, 2002, 41, 12284–12296. 12369816 10.1021/bi025702a

[211] Vriens K, , The antifungal plant defensin AtPDF2.3 from Arabidopsis thaliana blocks potassium channels, Sci. Rep, 2016, 6, 32121. 27573545 10.1038/srep32121 PMC5004176

[212] de Beer A and Vivier MA, Four plant defensins from an indigenous South African Brassicaceae species display divergent activities against two test pathogens despite high sequence similarity in the encoding genes, BMC Res. Notes, 2011, 4, 459. 22032337 10.1186/1756-0500-4-459 PMC3213222

[213] Slavokhotova AA, , Isolation, molecular cloning and antimicrobial activity of novel defensins from common chickweed (Stellaria media L.) seeds, Biochimie, 2011, 93, 450–456. 21056078 10.1016/j.biochi.2010.10.019

[214] Liu Y-J, , Solution structure of the plant defensin VrD1 from mung bean and its possible role in insecticidal activity against bruchids, Proteins, 2006, 63, 777–786. 16544327 10.1002/prot.20962

[215] Pinheiro-Aguiar R, do Amaral VSG, Pereira IB, Kurtenbach E and Almeida FCL, Nuclear magnetic resonance solution structure of *Pisum sativum* defensin 2 provides evidence for the presence of hydrophobic surface-clusters, Proteins, 2020, 88, 242–246. 31294889 10.1002/prot.25783

[216] Shenkarev ZO, , Heterologous expression and solution structure of defensin from lentil Lens culinaris, Biochem. Biophys. Res. Commun, 2014, 451, 252–257. 25086358 10.1016/j.bbrc.2014.07.104

[217] Sagaram US, , Structural and functional studies of a phosphatidic acid-binding antifungal plant defensin MtDef4: Identification of an RGFRRR motif governing fungal cell entry, PLoS One, 2013, 8, e82485. 24324798 10.1371/journal.pone.0082485 PMC3853197

[218] Almeida MS, Cabral KMS, Kurtenbach E, Almeida FCL and Valente AP, Solution structure of Pisum sativum defensin 1 by high resolution NMR: plant defensins, identical backbone with different mechanisms of action 1 1Edited by M. F. Summers, J. Mol. Biol, 2002, 315, 749–757. 11812144 10.1006/jmbi.2001.5252

[219] Li H, Velivelli SLS and Shah DM, Antifungal potency and modes of action of a novel Olive tree defensin against closely related Ascomycete fungal pathogens, Mol. Plant Microbe Interact, 2019, 32, 1649–1664. 31425003 10.1094/MPMI-08-19-0224-R

[220] Kovaleva V, Krynytskyy H, Gout I and Gout R, Recombinant expression, affinity purification and functional characterization of Scots pine defensin 1, Appl. Microbiol. Biotechnol, 2011, 89, 1093–1101. 20957359 10.1007/s00253-010-2935-2

[221] Bloch C and Richardson M, A new family of small (5 kDa) protein inhibitors of insect α-amylases from seeds or sorghum (*Sorghum bicolor*(L) Moench) have sequence homologies with wheat γ-purothionins, FEBS Lett, 1991, 279, 101–104. 1995329 10.1016/0014-5793(91)80261-z

[222] Odintsova TI, , Seed defensins of barnyard grass Echinochloa crusgalli (L.) Beauv, Biochimie, 2008, 90, 1667–1673. 18625284 10.1016/j.biochi.2008.06.007

[223] Tantong S, , Two novel antimicrobial defensins from rice identified by gene coexpression network analyses, Peptides, 2016, 84, 7–16. 27527801 10.1016/j.peptides.2016.07.005

[224] Rogozhin EA, , Novel antifungal defensins from Nigella sativa L. seeds, Plant Physiol. Biochem, 2011, 49, 131–137. 21144761 10.1016/j.plaphy.2010.10.008

[225] Fant F, Vranken WF and Borremans FAM, The three-dimensional solution structure of Aesculus hippocastanum antimicrobial protein 1 determined by 1H nuclear magnetic resonance, Proteins, 1999, 37, 388–403. 10591099 10.1002/(sici)1097-0134(19991115)37:3<388::aid-prot7>3.3.co;2-6

[226] Aerts AM, , The Antifungal Plant Defensin HsAFP1 from Heuchera Sanguinea Induces Apoptosis in Candida Albicans, Front. Microbiol, 2011, 2, 47. 21993350 10.3389/fmicb.2011.00047 PMC3128936

[227] Lay FT, Schirra HJ, Scanlon MJ, Anderson MA and Craik DJ, The three-dimensional solution structure of NaD1, a new floral defensin from Nicotiana alata and its application to a homology model of the crop defense protein alfAFP, J. Mol. Biol, 2003, 325, 175–188. 12473460 10.1016/s0022-2836(02)01103-8

[228] Janssen BJC, Schirra HJ, Lay FT, Anderson MA and Craik DJ, Structure of petunia hybrida defensin 1, a novel plant defensin with five disulfide bonds, Biochemistry, 2003, 42, 8214–8222. 12846570 10.1021/bi034379o

[229] de Beer A and Vivier MA, Vv-AMP1, a ripening induced peptide from Vitis vinifera shows strong antifungal activity, BMC Plant Biol., 2008, 8, 75. 18611251 10.1186/1471-2229-8-75 PMC2492866

[230] Nielsen KK, Nielsen JE, Madrid SM and Mikkelsen JD, Characterization of a new antifungal chitin-binding peptide from sugar beet leaves, Plant Physiol., 1997, 113, 83–91. 9008390 10.1104/pp.113.1.83 PMC158118

[231] Loo S, , Anti-fungal hevein-like peptides biosynthesized from quinoa cleavable hololectins, Molecules, 2021, 26, 5909. 34641455 10.3390/molecules26195909 PMC8512870

[232] Kini SG, , Studies on the chitin binding property of novel cysteine-rich peptides from *Alternanthera sessilis*, Biochemistry, 2015, 54, 6639–6649. 26467613 10.1021/acs.biochem.5b00872

[233] Martins JC, , 1H NMR study of the solution structure of ac-AMP2, a sugar binding antimicrobial protein isolated fromAmaranthus caudatus, J. Mol. Biol, 1996, 258, 322–333. 8627629 10.1006/jmbi.1996.0253

[234] Broekaert WF, , Antimicrobial peptides from Amaranthus caudatus seeds with sequence homology to the cysteine/glycine-rich domain of chitin-binding proteins, Biochemistry, 1992, 31, 4308–4314. 1567877 10.1021/bi00132a023

[235] Lipkin A, , An antimicrobial peptide Ar-AMP from amaranth (Amaranthus retroflexus L.) seeds, Phytochemistry, 2005, 66, 2426–2431. 16126239 10.1016/j.phytochem.2005.07.015

[236] Astafieva AA, , Discovery of novel antimicrobial peptides with unusual cysteine motifs in dandelion Taraxacum officinale Wigg. flowers, Peptides, 2012, 36, 266–271. 22640720 10.1016/j.peptides.2012.05.009

[237] Loo S, Kam A, Xiao T and Tam JP, Bleogens: Cactus-derived anti-candida cysteine-rich peptides with three different precursor arrangements, Front. Plant Sci, 2017, 8, 2162. 29312404 10.3389/fpls.2017.02162 PMC5743680

[238] Rogozhin EA, , A novel antifungal peptide from leaves of the weed Stellaria media L, Biochimie, 2015, 116, 125–132. 26196691 10.1016/j.biochi.2015.07.014

[239] Wong KH, , Vaccatides: Antifungal Glutamine-Rich Hevein-Like Peptides from Vaccaria hispanica, Front. Plant Sci, 2017, 8, 1100. 28680440 10.3389/fpls.2017.01100 PMC5478723

[240] Van den Bergh KPB, , Five disulfide bridges stabilize a hevein-type antimicrobial peptide from the bark of spindle tree (*Euonymus europaeus*L.), FEBS Lett, 2002, 530, 181–185. 12387889 10.1016/s0014-5793(02)03474-9

[241] Koo JC, , Two hevein homologs isolated from the seed of Pharbitis nil L. exhibit potent antifungal activity, Biochim. Biophys. Acta, 1998, 1382, 80–90. 9507071 10.1016/s0167-4838(97)00148-9

[242] Yokoyama S, , Purification, characterization, and sequencing of antimicrobial peptides, Cy-AMP1, Cy-AMP2, and Cy-AMP3, from the Cycad (Cycas revoluta) seeds, Peptides, 2008, 29, 2110–2117. 18778743 10.1016/j.peptides.2008.08.007

[243] Xiang Y, Huang R-H, Liu X-Z, Zhang Y and Wang D-C, Crystal structure of a novel antifungal protein distinct with five disulfide bridges from Eucommia ulmoides Oliver at an atomic resolution, J. Struct. Biol, 2004, 148, 86–97. 15363789 10.1016/j.jsb.2004.04.002

[244] Loo S, , Identification and characterization of roseltide, a knottin-type neutrophil elastase inhibitor derived from Hibiscus sabdariffa, Sci. Rep, 2016, 6, 39401. 27991569 10.1038/srep39401 PMC5171801

[245] Kam A, , Roseltide rT7 is a disulfide-rich, anionic, and cell-penetrating peptide that inhibits proteasomal degradation, J. Biol. Chem, 2019, 294, 19604–19615. 31727740 10.1074/jbc.RA119.010796 PMC6926453

[246] Cammue BP, , Isolation and characterization of a novel class of plant antimicrobial peptides form Mirabilis jalapa L. seeds, J. Biol. Chem, 1992, 267, 2228–2233. 1733929

[247] Gao G-H, , Solution structure of PAFP-S: A new knottin-type antifungal peptide from the seeds of Phytolacca americana, Biochemistry, 2001, 40, 10973–10978. 11551192 10.1021/bi010167k

[248] Li S-S and Claeson P, Cys/Gly-rich proteins with a putative single chitin-binding domain from oat (Avena sativa) seeds, Phytochemistry, 2003, 63, 249–255. 12737975 10.1016/s0031-9422(03)00116-x

[249] Odintsova TI, , A novel antifungal hevein-type peptide from Triticum kiharae seeds with a unique 10-cysteine motif, FEBS J, 2009, 276, 4266–4275. 19583772 10.1111/j.1742-4658.2009.07135.x

[250] Loo S, Tay SV, Kam A, Lee W and Tam JP, Hololectin interdomain linker determines asparaginyl endopeptidase-mediated maturation of antifungal hevein-like peptides in oats, Front. Plant Sci, 2022, 13, 899740. 35620686 10.3389/fpls.2022.899740 PMC9127739

[251] Fujimura M, Minami Y, Watanabe K and Tadera K, Purification, characterization, and sequencing of a novel type of antimicrobial peptides,*fa*-AMP1 and*fa*-AMP2, from seeds of buckwheat (*Fagopyrum esculentum*Moench.), Biosci., Biotechnol., Biochem, 2003, 67, 1636–1642. 12951494 10.1271/bbb.67.1636

[252] Wong KH, Tan WL, Xiao T and Tam JP, β-Ginkgotides: Hyperdisulfide-constrained peptides from Ginkgo biloba, Sci. Rep, 2017, 7, 6140. 28733600 10.1038/s41598-017-06598-x PMC5522442

[253] He M, , Discovery of a cysteine-rich peptide with glycation modification from Achyranthes bidentata Blume, Fitoterapia, 2022, 163, 105338. 36270560 10.1016/j.fitote.2022.105338

[254] Marcus JP, Green JL, Goulter KC and Manners JM, A family of antimicrobial peptides is produced by processing of a 7S globulin protein in Macadamia integrifolia kernels, Plant J, 1999, 19, 699–710. 10571855 10.1046/j.1365-313x.1999.00569.x

[255] Duvick JP, Rood T, Rao AG and Marshak DR, Purification and characterization of a novel antimicrobial peptide from maize (Zea mays L.) kernels, J. Biol. Chem, 1992, 267, 18814–18820. 1527010

[256] Campbell L and Turner SR, A comprehensive analysis of RALF proteins in green plants suggests there are two distinct functional groups, Front. Plant Sci, 2017, 8, 37. 28174582 10.3389/fpls.2017.00037 PMC5258720

[257] Svensson B, , Primary structure of barwin: a barley seed protein closely related to the C-terminal domain of proteins encoded by wound-induced plant genes, Biochemistry, 1992, 31, 8767–8770. 1390663 10.1021/bi00152a012

[258] Arolas JL, Aviles FX, Chang J-Y and Ventura S, Folding of small disulfide-rich proteins: clarifying the puzzle, Trends Biochem. Sci, 2006, 31, 292–301. 16600598 10.1016/j.tibs.2006.03.005

[259] Venhudová G, Canals F, Querol E and Aviles FX, Mutations in the N- and C-terminal tails of potato carboxypeptidase inhibitor influence its oxidative refolding process at the reshuffling stage, J. Biol. Chem, 2001, 276, 11683–11690. 11152676 10.1074/jbc.M007927200

[260] Le-Nguyen D, Heitz A, Chiche L, Hajji ME and Castro B, Characterization and 2D NMR study of the stable [9-21, 15-27] 2 disulfide intermediate in the folding of the 3 disulfide trypsin inhibitor EETI II, Protein Sci, 1993, 2, 165–174. 8443596 10.1002/pro.5560020205 PMC2142350

[261] He W-J, , Novel inhibitor cystine knot peptides from Momordica charantia, PLoS One, 2013, 8, e75334. 24116036 10.1371/journal.pone.0075334 PMC3792974

[262] Laps S, , Insight on the order of regioselective ultrafast formation of disulfide bonds in (antimicrobial) peptides and miniproteins, Angew Chem. Int. Ed. Engl, 2021, 60, 24137–24143. 34524726 10.1002/anie.202107861

[263] Romero A, Alamillo JM and García-Olmedo F, Processing of thionin precursors in barley leaves by a vacuolar proteinase, Eur. J. Biochem, 1997, 243, 202–208. 9030740 10.1111/j.1432-1033.1997.0202a.x

[264] Plattner S, , Isolation and characterization of a thionin proprotein-processing enzyme from barley, J. Biol. Chem, 2015, 290, 18056–18067. 26013828 10.1074/jbc.M115.647859 PMC4505051

[265] Reimann-Philipp U, Schrader G, Martinoia E, Barkholt V and Apel K, Intracellular thionins of barley. A second group of leaf thionins closely related to but distinct from cell wall-bound thionins, J. Biol. Chem, 1989, 264, 8978–8984. 2722812

[266] Bohlmann H, , Leaf-specific thionins of barley-a novel class of cell wall proteins toxic to plant-pathogenic fungi and possibly involved in the defence mechanism of plants, EMBO J, 1988, 7, 1559–1565. 16453847 10.1002/j.1460-2075.1988.tb02980.x PMC457137

[267] Quilis J, , A potato carboxypeptidase inhibitor gene provides pathogen resistance in transgenic rice, Plant Biotechnol. J, 2007, 5, 537–553. 17547659 10.1111/j.1467-7652.2007.00264.x

[268] Chagolla-Lopez A, Blanco-Labra A, Patthy A, Sánchez R and Pongor S, A novel alpha-amylase inhibitor from amaranth (Amaranthus hypocondriacus) seeds, J. Biol. Chem, 1994, 269, 23675–23680. 8089137

[269] Lu S, , Solution structure of the major alpha-amylase inhibitor of the crop plant amaranth, J. Biol. Chem, 1999, 274, 20473–20478. 10400675 10.1074/jbc.274.29.20473

[270] Pereira PJB, , Specific inhibition of insect α-amylases: yellow meal worm α-amylase in complex with the Amaranth α-amylase inhibitor at 2.0 Å resolution, Structure, 1999, 7, 1079–1088. 10508777 10.1016/s0969-2126(99)80175-0

[271] Slavokhotova AA, , Novel mode of action of plant defense peptides - hevein-like antimicrobial peptides from wheat inhibit fungal metalloproteases, FEBS J, 2014, 281, 4754–4764. 25154438 10.1111/febs.13015

[272] Florack DEA, Visser B, Vries PM, Vuurde JWL and Stiekema WJ, Analysis of the toxicity of purothionins and hordothionins for plant pathogenic bacteria, Neth. J. Plant Pathol, 1993, 99, 259–268.

[273] Molina A, Goy PA, Fraile A, Sánchez-Monge R and García-Olmedo F, Inhibition of bacterial and fungal plant pathogens by thionins of types I and II, Plant Sci, 1993, 92, 169–177.

[274] Ohtani S, Okada T, Yoshizumi H and Kagamiyama H, Complete primary structures of two subunits of purothionin A, a lethal protein for brewer’s yeast from wheat flour, J. Biochem, 1977, 82, 753–767. 914810 10.1093/oxfordjournals.jbchem.a131752

[275] Stec B, , Proposal for molecular mechanism of thionins deduced from physico-chemical studies of plant toxins, J. Pept. Res, 2004, 64, 210–224. 15613085 10.1111/j.1399-3011.2004.00187.x

[276] Gao AG, , Fungal pathogen protection in potato by expression of a plant defensin peptide, Nat. Biotechnol, 2000, 18, 1307–1310. 11101813 10.1038/82436

[277] Vijayan S, Singh NK, Shukla P and Kirti PB, Defensin (TvD1) from Tephrosia villosa exhibited strong anti-insect and anti-fungal activities in transgenic tobacco plants, J. Pest Sci, 2013, 86, 337–344.

[278] Velivelli SLS, Islam KT, Hobson E and Shah DM, Modes of action of a bi-domain plant defensin MtDef5 against a bacterial pathogen Xanthomonas campestris, Front. Microbiol, 2018, 9, 934. 29867843 10.3389/fmicb.2018.00934 PMC5964164

[279] Pelegrini PB, Lay FT, Murad AM, Anderson MA and Franco OL, Novel insights on the mechanism of action of α-amylase inhibitors from the plant defensin family, Proteins, 2008, 73, 719–729. 18498107 10.1002/prot.22086

[280] Berrocal-Lobo M, , Snakin-2, an antimicrobial peptide from potato whose gene is locally induced by wounding and responds to pathogen infection, Plant Physiol, 2002, 128, 951–961. 11891250 10.1104/pp.010685 PMC152207

[281] Herbel V and Wink M, Mode of action and membrane specificity of the antimicrobial peptide snakin-2, PeerJ, 2016, 4, e1987. 27190708 10.7717/peerj.1987 PMC4867707

[282] Herbel V, Schäfer H and Wink M, Recombinant production of snakin-2 (an antimicrobial peptide from tomato) in E. coli and analysis of its bioactivity, Molecules, 2015, 20, 14889–14901. 26287145 10.3390/molecules200814889 PMC6332222

[283] Horváth B, , Loss of the nodule-specific cysteine rich peptide, NCR169, abolishes symbiotic nitrogen fixation in the Medicago truncatula dnf7 mutant, Proc. Natl. Acad. Sci. U. S. A, 2015, 112, 15232–15237. 26401023 10.1073/pnas.1500777112 PMC4679056

[284] Zhang R, , Nodule-specific cysteine-rich peptide 343 is required for symbiotic nitrogen fixation in *Medicago truncatula*, Plant Physiol, 2023, 193, 1897–1912. 37555448 10.1093/plphys/kiad454

[285] Alunni B and Gourion B, Terminal bacteroid differentiation in the legume–rhizobium symbiosis: nodule-specific cysteine-rich peptides and beyond, New Phytol, 2016, 211, 411–417. 27241115 10.1111/nph.14025

[286] Sankari S, , A haem-sequestering plant peptide promotes iron uptake in symbiotic bacteria, Nat. Microbiol, 2022, 7, 1453–1465. 35953657 10.1038/s41564-022-01192-y PMC9420810

[287] Lee OS, , Pn-AMPs, the hevein-like proteins from Pharbitis nil confers disease resistance against phytopathogenic fungi in tomato, Lycopersicum esculentum, Phytochemistry, 2003, 62, 1073–1079. 12591259 10.1016/s0031-9422(02)00668-4

[288] Choon Koo J, , Plant Mol. Biol, 2002, 50, 441–452. 12369620 10.1023/a:1019864222515

[289] Kanrar S, Venkateswari JC, Kirti PB and Chopra VL, Transgenic expression of hevein, the rubber tree lectin, in Indian mustard confers protection against Alternaria brassicae, Plant Sci, 2002, 162, 441–448.

[290] Das K, Datta K, Sarkar SN and Datta SK, Expression of antimicrobial peptide snakin-1 confers effective protection in rice against sheath blight pathogen, Rhizoctonia solani, Plant Biotechnol. Rep, 2021, 15, 39–54.

[291] Cox N, Kintzing JR, Smith M, Grant GA and Cochran JR, Integrin-targeting knottin peptide-drug conjugates are potent inhibitors of tumor cell proliferation, Angew Chem. Int. Ed. Engl, 2016, 55, 9894–9897. 27304709 10.1002/anie.201603488 PMC6231717

[292] Glotzbach B, , Combinatorial optimization of cystine-knot peptides towards high-affinity inhibitors of human matriptase-1, PLoS One, 2013, 8, e76956. 24146945 10.1371/journal.pone.0076956 PMC3795654

[293] Gilding EK, , Neurotoxic peptides from the venom of the giant Australian stinging tree, Sci. Adv, 2020, 6, eabb8828. 32938666 10.1126/sciadv.abb8828 PMC7494335

[294] Jami S, , Pain-causing stinging nettle toxins target TMEM233 to modulate NaV1.7 function, Nat. Commun, 2023, 14, 2442. 37117223 10.1038/s41467-023-37963-2 PMC10147923

[295] Miao Z, , An engineered knottin peptide labeled with 18F for PET imaging of integrin expression, Bioconjugate Chem, 2009, 20, 2342–2347. 10.1021/bc900361g PMC2804269 19908826

[296] Kaufmann C and Sauter M, Sulfated plant peptide hormones, J. Exp. Bot, 2019, 70, 4267–4277. 31231771 10.1093/jxb/erz292 PMC6698702

[297] Petersen BL, MacAlister CA and Ulvskov P, Plant protein O-arabinosylation, Front. Plant Sci, 2021, 12, 645219. 33815452 10.3389/fpls.2021.645219 PMC8012813

[298] Matsubayashi Y, Posttranslationally modified small-peptide signals in plants, Annu. Rev. Plant Biol, 2014, 65, 385–413. 24779997 10.1146/annurev-arplant-050312-120122

[299] Kim JS, Jeon BW and Kim J, Signaling peptides regulating abiotic stress responses in plants, Front. Plant Sci, 2021, 12, 704490. 34349774 10.3389/fpls.2021.704490 PMC8326967

[300] Matsubayashi Y, Takagi L and Sakagami Y, Phytosulfokine-α, a sulfated pentapeptide, stimulates the proliferation of rice cells by means of specific high- and low-affinity binding sites, Proc. Natl. Acad. Sci. U. S. A, 1997, 94, 13357–13362. 9371850 10.1073/pnas.94.24.13357 PMC24313

[301] Yang H, Matsubayashi Y, Nakamura K and Sakagami Y, Oryza sativa PSK gene encodes a precursor of phytosulfokine-alpha, a sulfated peptide growth factor found in plants, Proc. Natl. Acad. Sci. U. S. A, 1999, 96, 13560–13565. 10557360 10.1073/pnas.96.23.13560 PMC23987

[302] Nakayama T, , A peptide hormone required for Casparian strip diffusion barrier formation in Arabidopsis roots, Science, 2017, 355, 284–286. 28104889 10.1126/science.aai9057

[303] Doblas VG, , Root diffusion barrier control by a vasculature-derived peptide binding to the SGN3 receptor, Science, 2017, 355, 280–284. 28104888 10.1126/science.aaj1562

[304] Matsuzaki Y, Ogawa-Ohnishi M, Mori A and Matsubayashi Y, Secreted peptide signals required for maintenance of root stem cell niche in Arabidopsis, Science, 2010, 329, 1065–1067. 20798316 10.1126/science.1191132

[305] Amano Y, Tsubouchi H, Shinohara H, Ogawa M and Matsubayashi Y, Tyrosine-sulfated glycopeptide involved in cellular proliferation and expansion in Arabidopsis, Proc. Natl. Acad. Sci. U. S. A, 2007, 104, 18333–18338. 17989228 10.1073/pnas.0706403104 PMC2084343

[306] Ito Y, , Dodeca-CLE peptides as suppressors of plant stem cell differentiation, Science, 2006, 313, 842–845. 16902140 10.1126/science.1128436

[307] Chrispeels MJ, Prolyl hydroxylase in plants, in Methods in Enzymology, Academic Press, 1984, vol. 107, pp. 361–369.

[308] Stührwohldt N, Ehinger A, Thellmann K and Schaller A, Processing and formation of bioactive CLE40 peptide are controlled by posttranslational proline hydroxylation, Plant Physiol, 2020, 184, 1573–1584. 32907884 10.1104/pp.20.00528 PMC7608152

[309] Takahashi F, , A small peptide modulates stomatal control via abscisic acid in long-distance signalling, Nature, 2018, 556, 235–238. 29618812 10.1038/s41586-018-0009-2

[310] Stührwohldt N, Bühler E, Sauter M and Schaller A, Phytosulfokine (PSK) precursor processing by subtilase SBT3.8 and PSK signaling improve drought stress tolerance in Arabidopsis, J. Exp. Bot, 2021, 72, 3427–3440. 33471900 10.1093/jxb/erab017

[311] Tabata R, , Perception of root-derived peptides by shoot LRR-RKs mediates systemic N-demand signaling, Science, 2014, 346, 343–346. 25324386 10.1126/science.1257800

[312] Luu DD, , Biosynthesis and secretion of the microbial sulfated peptide RaxX and binding to the rice XA21 immune receptor, Proc. Natl. Acad. Sci. U. S. A, 2019, 116, 8525–8534. 30948631 10.1073/pnas.1818275116 PMC6486716

[313] Hattori J, Boutilier KA, van Lookeren Campagne MM and Miki BL, A conserved BURP domain defines a novel group of plant proteins with unusual primary structures, Mol. Gen. Genet, 1998, 259, 424–428. 9790599 10.1007/s004380050832

[314] Treacy BK, , Bnm1, a Brassica pollen-specific gene, Plant Mol. Biol, 1997, 34, 603–611. 9247542 10.1023/a:1005851801107

[315] Van Son L, , The BURP domain protein AtUSPL1 of Arabidopsis thaliana is destined to the protein storage vacuoles and overexpression of the cognate gene distorts seed development, Plant Mol. Biol, 2009, 71, 319–329. 19639386 10.1007/s11103-009-9526-6

[316] Yamaguchi-Shinozaki K and Shinozaki K, The plant hormone abscisic acid mediates the drought-induced expression but not the seed-specific expression of rd22, a gene responsive to dehydration stress in Arabidopsis thaliana, Mol. Gen. Genet, 1993, 238, 17–25. 8479424 10.1007/BF00279525

[317] Zheng L, Heupel RC and DellaPenna D, The beta subunit of tomato fruit polygalacturonase isoenzyme 1: isolation, characterization, and identification of unique structural features, Plant Cell, 1992, 4, 1147–1156. 1392611 10.1105/tpc.4.9.1147 PMC160205

[318] Mydy LS, Chigumba DN and Kersten RD, Plant copper metalloenzymes as prospects for new metabolism involving aromatic compounds, Front. Plant Sci, 2021, 12, 692108. 34925392 10.3389/fpls.2021.692108 PMC8672867

[319] Ragland M and Soliman KM, Sali5-4a and Sali3-2. Two genes induced by aluminum in soybean roots, Plant Physiol, 1997, 114, 395.9159957

[320] Xu H, , Genome-scale identification of soybean BURP domain-containing genes and their expression under stress treatments, BMC Plant Biol, 2010, 10, 197. 20836857 10.1186/1471-2229-10-197 PMC2956546

[321] Xun H, , Over-expression of GmKR3, a TIR-NBS-LRR type R gene, confers resistance to multiple viruses in soybean, Plant Mol. Biol, 2019, 99, 95–111. 30535849 10.1007/s11103-018-0804-z

[322] Chen T, , Construction of high-density genetic map and identification of a bruchid resistance locus in mung bean (Vigna radiata L.), Front. Genet, 2022, 13, 903267. 35873485 10.3389/fgene.2022.903267 PMC9305327

[323] Yahara S, , Cyclic Peptides, Acyclic Diterpene Glycosides and Other Compounds from Lycium chinense MILL, Chem. Pharm. Bull, 1993, 41, 703–709, DOI: 10.1248/cpb.41.703. 8508472

[324] Morita H, Yoshida N, Takeya K, Itokawa H and Shirota O, Configurational and conformational analyses of a cyclic octapeptide, lyciumin A, from Lycium chinense Mill, Tetrahedron, 1996, 52, 2795–2802.

[325] Morita H, Suzuki H and Kobayashi J, Celogenamide A, a new cyclic peptide from the seeds of *Celosia argentea*, J. Nat. Prod, 2004, 67, 1628–1630. 15387679 10.1021/np049858i

[326] Tang Y, , Expression of a vacuole-localized BURP-domain protein from soybean (SALI3-2) enhances tolerance to cadmium and copper stresses, PLoS One, 2014, 9, e98830. 24901737 10.1371/journal.pone.0098830 PMC4047006

[327] Mydy LS, , An intramolecular macrocyclase in plant ribosomal peptide biosynthesis, Nat. Chem. Biol, 2024, DOI: 10.1038/s41589-024-01552-1. PMC11049724 38355722

[328] Messerschmidt A, 5.11 – Copper Metalloenzymes, in *Comprehensive Natural Products III*, ed. Liu H-W and Begley TP, Elsevier, 2010, pp. 251–297.

[329] Song H, , A molecular mechanism for the enzymatic methylation of nitrogen atoms within peptide bonds, Sci. Adv, 2018, 4, eaat2720. 30151425 10.1126/sciadv.aat2720 PMC6108569

[330] Tschesche R and Kaußmann EU, Chapter 4 the cyclopeptide alkaloids, in Chemistry and Physiology, Elsevier, 1975, pp. 165–205.

[331] Tuenter E, Exarchou V, Apers S and Pieters L, Cyclopeptide alkaloids, Phytochem. Rev, 2017, 16, 623–637.

[332] Gournelis DC, Laskaris GG and Verpoorte R, Cyclopeptide alkaloids, Nat. Prod. Rep, 1997, 14, 75–82. 9121730 10.1039/np9971400075

[333] Kang KB, , Jubanines F–J, cyclopeptide alkaloids from the roots of Ziziphus jujuba, Phytochemistry, 2015, 119, 90–95. 26361730 10.1016/j.phytochem.2015.09.001 PMC7111685

[334] Tschesche R and Last H, Alkaloide aus rhamnaceen, V franganin und frangufolin, zwei weitere peptid-alkaloide aus L, Tetrahedron Lett, 1968, 9, 2993–2998. 10.1016/s0040-4039(00)89630-6 5651010

[335] Tschesche R, , Alkaloide aus Rhamnaceen, XIX1) Mucronin-E,-F,-G und-H sowie Abyssenin-A,-B und-C, weitere 15 gliedrige Cyclopeptidalkaloide, Justus Liebigs Ann. Chem, 1974, 1915–1928.

[336] Retailleau P, Numbi Wa Ilunga E, Fontaine V, Gallard J-F and Le Pogam P, Clarifying the configuration of pandamine by an extensive spectroscopic reinvestigation of the authentic 1964 sample, Metabolites, 2023, 13, 470. 37110129 10.3390/metabo13040470 PMC10147048

[337] Lin H-Y, Chen C-H, Chen Liu KCS and Lee S-S, 14-Membered Cyclopeptides fromPaliurus ramosissimus andP. hemsleyanus, Helv. Chim. Acta, 2003, 86, 127–138.

[338] Giacomelli SR, , Cyclic peptide alkaloids from the bark of Discaria americana, Phytochemistry, 2004, 65, 933–937. 15081298 10.1016/j.phytochem.2004.02.006

[339] Han J, , Cyclopeptide alkaloids from Ziziphus apetala, J. Nat. Prod, 2011, 74, 2571–2575. 22148241 10.1021/np200755t

[340] Shabani S, White JM and Hutton CA, Total synthesis of the putative structure of asperipin-2a and stereochemical reassignment, Org. Lett, 2020, 22, 7730–7734. 32960070 10.1021/acs.orglett.0c02884

[341] Zhang Z, , Jujube metabolome selection determined the edible properties acquired during domestication, Plant J, 2022, 109, 1116–1133. 34862996 10.1111/tpj.15617

[342] Ma Y, , Sanjoinine A isolated from zizyphi spinosi semen augments pentobarbital-induced sleeping behaviors through the modification of GABA-ergic systems, Biol. Pharm. Bull, 2007, 30, 1748–1753. 17827733 10.1248/bpb.30.1748

[343] Han H, , Anxiolytic-like effects of sanjoinine A isolated from Zizyphi Spinosi Semen: possible involvement of GABAergic transmission, Pharmacol., Biochem. Behav, 2009, 92, 206–213. 19101585 10.1016/j.pbb.2008.11.012

[344] Trevisan G, , Antinociceptive effects of 14-membered cyclopeptide alkaloids, J. Nat. Prod, 2009, 72, 608–612. 19231884 10.1021/np800377y

[345] Kaleem WA, , Antinociceptive activity of cyclopeptide alkaloids isolated from Ziziphus oxyphylla Edgew (Rhamnaceae), Fitoterapia, 2013, 91, 154–158. 24013037 10.1016/j.fitote.2013.08.024

[346] Hwang KH, Han YN and Han BH, Inhibition of calmodulin-dependent Calcium-ATPase and phosphodiesterase by various cyclopeptides and peptide alkaloids from the zizyphus species, Arch. Pharmacal Res, 2001, 24, 202–206. 10.1007/BF02978257 11440077

[347] Tuenter E, , Antiplasmodial activity, cytotoxicity and structure-activity relationship study of cyclopeptide alkaloids, Molecules, 2017, 22, 224. 28157177 10.3390/molecules22020224 PMC6155665

[348] Tuenter E, , Cyclopeptide alkaloids from *Hymenocardia acida*, J. Nat. Prod, 2016, 79, 1746–1751. 27351950 10.1021/acs.jnatprod.6b00131

[349] Sugawara F, , Insecticidal Peptide from Mungbean: A Resistant Factor against Infestation with Azuki Bean Weevil, J. Agric. Food Chem, 1996, 44, 3360–3364.

[350] Kaga A and Ishimoto M, Genetic localization of a bruchid resistance gene and its relationship to insecticidal cyclopeptide alkaloids, the vignatic acids, in mungbean (Vigna radiata L. Wilczek), Mol. Gen. Genet, 1998, 258, 378–384. 9648742 10.1007/s004380050744

[351] Yoshikawa K, Tao S and Arihara S, Stephanotic acid, a novel cyclic pentapeptide from the stem of Stephanotis floribunda, J. Nat. Prod, 2000, 63, 540–542. 10785436 10.1021/np990532x

[352] Xu X, , Moroidin, a cyclopeptide from the seeds of Celosia cristata that induces apoptosis in A549 human lung cancer cells, J. Nat. Prod, 2022, 85, 1918–1927. 35951980 10.1021/acs.jnatprod.1c01215

[353] Suzuki H, Morita H, Shiro M and Kobayashi J, Celogentin K, a new cyclic peptide from the seeds of Celosia argentea and X-ray structure of moroidin, Tetrahedron, 2004, 60, 2489–2495.

[354] Kobayashi J, Suzuki H, Shimbo K, Takeya K and Morita H, Celogentins A-C, new antimitotic bicyclic peptides from the seeds of Celosia argentea, J. Org. Chem, 2001, 66, 6626–6633. 11578213 10.1021/jo0103423

[355] Suzuki H, Morita H, Iwasaki S and Kobayashi J, New antimitotic bicyclic peptides, celogentins D–H, and J, from the seeds of Celosia argentea, Tetrahedron, 2003, 59, 5307–5315. 10.1021/jo0103423 11578213

[356] Christina Leung T-W, Williams DH, C J Barna J, Foti S and Oelrichs BP, Structural studies on the peptide moroidin from laportea moroides, Tetrahedron, 1986, 42, 3333–3348.

[357] Yun B-S, Ryoo I-J, Lee I-K and Yoo I-D, Hibispeptin A, a novel cyclic peptide from Hibiscus syriacus, Tetrahedron Lett, 1998, 39, 993–996.

[358] Yun B-S, Ryoo I-J, Lee I-K and Yoo I-D, Hibispeptin B, a novel cyclic peptide from Hibiscus syriacus, Tetrahedron, 1998, 54, 15155–15160.

[359] Toumi M, , A general route to cyclopeptide alkaloids: Total syntheses and biological evaluation of paliurines E and F, ziziphines N and Q, abyssenine A, mucronine E, and analogues, Eur. J. Org. Chem, 2009, 3368–3386.

[360] Abdalla MA and Matasyoh JC, Endophytes as producers of peptides: An overview about the recently discovered peptides from endophytic microbes, Nat. Prod. Bioprospect, 2014, 4, 257–270. 25205333 10.1007/s13659-014-0038-y PMC4199945

[361] Schafhauser T, , Antitumor astins originate from the fungal endophyte *Cyanodermella asteris* living within the medicinal plant *Aster tataricus*, Proc. Natl. Acad. Sci. U. S. A, 2019, 116, 26909–26917. 31811021 10.1073/pnas.1910527116 PMC6936678

[362] Jolad SD, , Bouvardin and deoxybouvardin, antitumor cyclic hexapeptides from Bouvardia ternifolia (Rubiaceae), J. Am. Chem. Soc, 1977, 99, 8040–8044. 591683 10.1021/ja00466a043

[363] Itokawa H, , Studies on antitumor cyclic hexapeptides RA obtained from Rubiae Radix, Rubiaceae. VI Minor antitumor constituents, Chem. Pharm. Bull, 1986, 34, 3762–3768. 10.1248/cpb.34.3762 3815597

[364] Itokawa H, , Studies on the antitumor cyclic hexapeptides obtained from Rubiae radix, Chem. Pharm. Bull, 1983, 31, 1424–1427. 10.1248/cpb.31.1424 6627519

[365] Fan J-T, , Rubiyunnanins A and B, two novel cyclic hexapeptides from Rubia yunnanensis, Tetrahedron Lett, 2010, 51, 6810–6813.

[366] Fan J-T, , Rubiyunnanins C–H, cytotoxic cyclic hexapeptides from Rubia yunnanensis inhibiting nitric oxide production and NF-κB activation, Bioorg. Med. Chem, 2010, 18, 8226–8234. 21044847 10.1016/j.bmc.2010.10.019

[367] Chen X-Q, , Rubicordins A–C, new cyclopeptides from Rubia cordifolia with cytotoxicity and inhibiting NF-κB signaling pathway, Tetrahedron, 2015, 71, 9673–9678.

[368] Feng L, , Diversity of cultivable endophytic fungi in two *Rubia* plants and their potential for production of anti-tumour Rubiaceae-type cyclopeptides, Lett. Appl. Microbiol, 2021, 73, 759–769. 34591984 10.1111/lam.13571

[369] Zalacaín M, Zaera E, Vázquez D and Jiménez A, The mode of action of the antitumor drug bouvardin, an inhibitor of protein synthesis in eukaryotic cells, FEBS Lett, 1982, 148, 95–97. 6924616 10.1016/0014-5793(82)81250-7

[370] Stickel SA, Gomes NP, Frederick B, Raben D and Su TT, Bouvardin is a radiation modulator with a novel mechanism of action, Radiat. Res, 2015, 184, 392. 26414509 10.1667/RR14068.1 PMC4643058

[371] Itokawa H, , Isolation and antitumor activity of cyclic hexapeptides isolated from Rubiae radix, Chem. Pharm. Bull, 1984, 32, 284–290. 10.1248/cpb.32.284 6722954

[372] Fang X-Y, , Plant cyclopeptide RA-V kills human breast cancer cells by inducing mitochondria-mediated apoptosis through blocking PDK1–AKT interaction, Toxicol. Appl. Pharmacol, 2013, 267, 95–103. 23274515 10.1016/j.taap.2012.12.010

[373] Leung H-W, , RA-XII inhibits tumour growth and metastasis in breast tumour-bearing mice via reducing cell adhesion and invasion and promoting matrix degradation, Sci. Rep, 2015, 5, 16985. 26592552 10.1038/srep16985 PMC4655310

[374] Yue GGL, , Cyclopeptide RA-V inhibits angiogenesis by down-regulating ERK1/2 phosphorylation in HUVEC and HMEC-1 endothelial cells, Br. J. Pharmacol, 2011, 164, 1883–1898. 21518338 10.1111/j.1476-5381.2011.01458.x PMC3246713

[375] An X and Shang F, RA-XII exerts anti-oxidant and anti-inflammatory activities on lipopolysaccharide-induced acute renal injury by suppressing NF-κB and MAPKs regulated by HO-1/Nrf2 pathway, Biochem. Biophys. Res. Commun, 2018, 495, 2317–2323. 29277609 10.1016/j.bbrc.2017.12.131

[376] Yu G, Smith DK, Zhu H, Guan Y and Lam TT-Y, ggtree: An r package for visualization and annotation of phylogenetic trees with their covariates and other associated data, Methods Ecol. Evol, 2017, 8, 28–36.

[377] Kirkpatrick CL, , The “PepSAVI-MS” pipeline for natural product bioactive peptide discovery, Anal. Chem, 2017, 89, 1194–1201. 27991763 10.1021/acs.analchem.6b03625 PMC8609470

[378] Parsley NC, , PepSAVI-MS reveals anticancer and antifungal cycloviolacins in Viola odorata, Phytochemistry, 2018, 152, 61–70. 29734037 10.1016/j.phytochem.2018.04.014 PMC6003877

[379] Moyer TB, , PepSAVI-MS Reveals a Proline-rich Antimicrobial Peptide in *Amaranthus tricolor*, J. Nat. Prod, 2019, 82, 2744–2753. 31557021 10.1021/acs.jnatprod.9b00352 PMC6874829

[380] Watrous J, , Mass spectral molecular networking of living microbial colonies, Proc. Natl. Acad. Sci. U. S. A, 2012, 109, E1743–E1752. 22586093 10.1073/pnas.1203689109 PMC3387089

[381] Mohimani H, , Dereplication of microbial metabolites through database search of mass spectra, Nat. Commun, 2018, 9, 4035. 30279420 10.1038/s41467-018-06082-8 PMC6168521

[382] Yang JY, , Molecular networking as a dereplication strategy, J. Nat. Prod, 2013, 76, 1686–1699. 24025162 10.1021/np400413s PMC3936340

[383] Nothias L-F, , Bioactivity-based molecular networking for the discovery of drug leads in natural product bioassay-guided fractionation, J. Nat. Prod, 2018, 81, 758–767. 29498278 10.1021/acs.jnatprod.7b00737

[384] Wang M, , Sharing and community curation of mass spectrometry data with Global Natural Products Social Molecular Networking, Nat. Biotechnol, 2016, 34, 828–837. 27504778 10.1038/nbt.3597 PMC5321674

[385] Wang M, , Mass spectrometry searches using MASST, Nat. Biotechnol, 2020, 38, 23–26. 31894142 10.1038/s41587-019-0375-9 PMC7236533

[386] Dührkop K, , SIRIUS 4: a rapid tool for turning tandem mass spectra into metabolite structure information, Nat. Methods, 2019, 16, 299–302. 30886413 10.1038/s41592-019-0344-8

[387] Dührkop K, , Systematic classification of unknown metabolites using high-resolution fragmentation mass spectra, Nat. Biotechnol, 2020, 39, 462–471. 33230292 10.1038/s41587-020-0740-8

[388] Archer BL, The proteins of Hevea brasiliensis Latex. 4. Isolation and characterization of crystalline hevein, Biochem. J, 1960, 75, 236–240. 13794068 10.1042/bj0750236 PMC1204415

[389] Nguyen GKT, , Discovery and characterization of novel cyclotides originated from chimeric precursors consisting of albumin-1 chain a and cyclotide domains in the Fabaceae family, J. Biol. Chem, 2011, 286, 24275–24287. 21596752 10.1074/jbc.M111.229922 PMC3129208

[390] Ireland DC, Clark RJ, Daly NL and Craik DJ, Isolation, sequencing, and Structure–Activity relationships of cyclotides, J. Nat. Prod, 2010, 73, 1610–1622. 20718473 10.1021/np1000413

[391] Moyer TB, Parsley NC, Sadecki PW, Schug WJ and Hicks LM, Leveraging orthogonal mass spectrometry based strategies for comprehensive sequencing and characterization of ribosomal antimicrobial peptide natural products, Nat. Prod. Rep, 2021, 38, 489–509. 32929442 10.1039/d0np00046a PMC7956910

[392] Shevchenko A, Wilm M, Vorm O and Mann M, Mass spectrometric sequencing of proteins from silver-stained polyacrylamide gels, Anal. Chem, 1996, 68, 850–858. 8779443 10.1021/ac950914h

[393] Parsley NC, Williams OL and Hicks LM, Exploring the diversity of cysteine-rich natural product peptides via MS/MS fingerprint ions, J. Am. Soc. Mass Spectrom, 2020, 31, 1833–1843. 32872784 10.1021/jasms.0c00078 PMC7816094

[394] Jarmusch AK, , A universal language for finding mass spectrometry data patterns, bioRxiv, 2022, preprint, DOI: 10.1101/2022.08.06.503000. PMC12334354 40355727

[395] Horai H, , MassBank: a public repository for sharing mass spectral data for life sciences, J. Mass Spectrom, 2010, 45, 703–714. 20623627 10.1002/jms.1777

[396] Smith CA, , METLIN: a metabolite mass spectral database, Ther. Drug Monit, 2005, 27, 747–751. 16404815 10.1097/01.ftd.0000179845.53213.39

[397] Salek RM, , The MetaboLights repository: curation challenges in metabolomics, Database, 2013, 2013, bat029. 23630246 10.1093/database/bat029 PMC3638156

[398] Behsaz B, , De Novo Peptide Sequencing Reveals Many Cyclopeptides in the Human Gut and Other Environments, Cell Syst., 2020, 10, 99–108. 31864964 10.1016/j.cels.2019.11.007

[399] Matasci N, , Data access for the 1,000 Plants (1KP) project, Gigascience, 2014, 3, 17. 25625010 10.1186/2047-217X-3-17 PMC4306014

[400] One Thousand Plant Transcriptomes Initiative, One thousand plant transcriptomes and the phylogenomics of green plants, Nature, 2019, 574, 679–685. 31645766 10.1038/s41586-019-1693-2 PMC6872490

[401] Goodstein DM, , Phytozome: a comparative platform for green plant genomics, Nucleic Acids Res, 2012, 40, D1178–D1186. 22110026 10.1093/nar/gkr944 PMC3245001

[402] Shelenkov A, Slavokhotova A and Odintsova T, Predicting antimicrobial and other cysteine-rich peptides in 1267 plant transcriptomes, Antibiotics, 2020, 9, 60. 32032999 10.3390/antibiotics9020060 PMC7168108

[403] Teufel F, , SignalP 6.0 predicts all five types of signal peptides using protein language models, Nat. Biotechnol, 2022, 40, 1023–1025. 34980915 10.1038/s41587-021-01156-3 PMC9287161

[404] Lau W and Sattely ES, Six enzymes from mayapple that complete the biosynthetic pathway to the etoposide aglycone, Science, 2015, 349, 1224–1228. 26359402 10.1126/science.aac7202 PMC6861171

[405] Schorn MA, , A community resource for paired genomic and metabolomic data mining, Nat. Chem. Biol, 2021, 17, 363–368. 33589842 10.1038/s41589-020-00724-z PMC7987574

